# Integrating single-cell sequencing data with GWAS summary statistics reveals CD16+monocytes and memory CD8+T cells involved in severe COVID-19

**DOI:** 10.1186/s13073-022-01021-1

**Published:** 2022-02-17

**Authors:** Yunlong Ma, Fei Qiu, Chunyu Deng, Jingjing Li, Yukuan Huang, Zeyi Wu, Yijun Zhou, Yaru Zhang, Yichun Xiong, Yinghao Yao, Yigang Zhong, Jia Qu, Jianzhong Su

**Affiliations:** 1 Institute of Biomedical Big Data, School of Ophthalmology & Optometry and Eye Hospital, School of Biomedical Engineering, Wenzhou Medical University, Wenzhou, 325027 China; 2 State Key Laboratory for Diagnosis and Treatment of Infectious Diseases, the First Affiliated Hospital, Collaborative Innovation Center for Diagnosis and Treatment of Infectious Diseases, Zhejiang University School of Medicine, Zhejiang, 310003 Hangzhou China; 3 Wenzhou Institute, University of Chinese Academy of Sciences, Wenzhou, 325011 China; 4 Department of Cardiology, Affiliated Hangzhou First People’s Hospital, Zhejiang University School of Medicine, Hangzhou, China

**Keywords:** Single-cell sequencing, GWAS, Immune cells, Inflammatory storm, COVID-19

## Abstract

**Background:**

Understanding the host genetic architecture and viral immunity contributes to the development of effective vaccines and therapeutics for controlling the COVID-19 pandemic. Alterations of immune responses in peripheral blood mononuclear cells play a crucial role in the detrimental progression of COVID-19. However, the effects of host genetic factors on immune responses for severe COVID-19 remain largely unknown.

**Methods:**

We constructed a computational framework to characterize the host genetics that influence immune cell subpopulations for severe COVID-19 by integrating GWAS summary statistics (*N* = 969,689 samples) with four independent scRNA-seq datasets containing healthy controls and patients with mild, moderate, and severe symptom (*N* = 606,534 cells). We collected 10 predefined gene sets including inflammatory and cytokine genes to calculate cell state score for evaluating the immunological features of individual immune cells.

**Results:**

We found that 34 risk genes were significantly associated with severe COVID-19, and the number of highly expressed genes increased with the severity of COVID-19. Three cell subtypes that are CD16+monocytes, megakaryocytes, and memory CD8+T cells were significantly enriched by COVID-19-related genetic association signals. Notably, three causal risk genes of *CCR1*, *CXCR6*, and *ABO* were highly expressed in these three cell types, respectively. *CCR1*^+^CD16+monocytes and *ABO*^+^ megakaryocytes with significantly up-regulated genes, including *S100A12*, *S100A8*, *S100A9*, and *IFITM1*, confer higher risk to the dysregulated immune response among severe patients. *CXCR6*^+^ memory CD8+ T cells exhibit a notable polyfunctionality including elevation of proliferation, migration, and chemotaxis. Moreover, we observed an increase in cell-cell interactions of both *CCR1*^+^ CD16+monocytes and *CXCR6*^+^ memory CD8+T cells in severe patients compared to normal controls among both PBMCs and lung tissues. The enhanced interactions of *CXCR6*^*+*^ memory CD8+T cells with epithelial cells facilitate the recruitment of this specific population of T cells to airways, promoting CD8+T cell-mediated immunity against COVID-19 infection.

**Conclusions:**

We uncover a major genetics-modulated immunological shift between mild and severe infection, including an elevated expression of genetics-risk genes, increase in inflammatory cytokines, and of functional immune cell subsets aggravating disease severity, which provides novel insights into parsing the host genetic determinants that influence peripheral immune cells in severe COVID-19.

**Supplementary Information:**

The online version contains supplementary material available at 10.1186/s13073-022-01021-1.

## Background

The coronavirus disease 2019 (COVID-19) outbreak, caused by severe acute respiratory syndrome coronavirus 2 (SARS-CoV-2), has widely and severely jeopardized the health and economy systems of most countries worldwide. As of January 26, 2022, there were more than 359.1 million confirmed patients with more than 5.63 million deaths in the whole world [1]. COVID-19 has distinct clinical manifestations ranging from asymptomatic to severe respiratory failure [2]. Mortalities of COVID-19 are largely derived from severe patients with interstitial pneumonia in both lungs and acute respiratory distress syndrome [3]. Many earlier studies [4–6] have shown that the number of severe COVID-19 patients who are elders and have comorbidities, such as diabetes and hypertension, has increased. In this connection, understanding the immunologic mechanism of severe COVID-19 and identifying novel vaccine targets to control the pandemic are of considerable interest.

Accumulating evidence has suggested that alterations of immune responses in peripheral blood mononuclear cells (PBMCs) and bronchoalveolar lavage fluid (BALF) play a crucial role in the detrimental progression of COVID-19 [7, 8]. There has been evidence that cytokine storm, usually found in severe COVID-19 patients, causes the adverse progression of COVID-19 [7]. Increased circulating levels of proinflammatory cytokine, including IL-10, IL-6, and TNF-α, have been reported to be associated with severe COVID-19 [7, 9]. Single-cell RNA sequencing (scRNA-seq) has been extensively utilized to reveal the immune responses of COVID-19 patients in both lung and peripheral blood [10–18]. Megakaryocytes and monocytes [11, 12], T cell exhaustion [14], lymphopenia [19], and increased levels of cytokines [20] may cause aberrant peripheral immune activities in severe COVID-19 patients. Based on large-scale samples, previous studies identified that dysregulation of the mTOR signaling pathway in dendritic cells [21] and aberrant myeloid cell subpopulations [16, 17] implicated in severe COVID-19. Su et al. [10] revealed an increase in inflammation and a sharp drop in blood nutrients between mild and moderate-to-severe COVID-19, and new subsets of immune cells emerged in moderate COVID-19 patients.

Genome-wide association study (GWAS) has emerged as a powerful approach to identify risk genes and genetic variants for complex diseases. By gathering population-based GWAS data worldwide, the COVID-19 Host Genetic Consortium has launched the “COVID-19 Host Genetics Initiative” project to facilitate COVID-19 host genetic research and identify genetic determinants of COVID-19 [22]. Subsequently, a growing number of GWASs have identified numerous significant genetic variants associated with COVID-19 susceptibility and severity [23–28]. Ellinghaus et al. [27] performed a meta-analysis of two independent GWAS datasets with 1610 severe COVID-19 patients and 2205 matched controls at seven hospitals in the Italian and Spanish epicenters and identified two susceptibility loci of 3p21.31 and 9q34.2 to be significantly associated with severe COVID-19 at the genome-wide level. Based on a large-scale meta-analysis (*N* = 680,128), our group found that the *IFNAR2-IL10RB* gene cluster was significantly associated with COVID-19 susceptibility, and suggested that *IFNAR2* and *IL10RB* might have regulatory roles in the pulmonary immune response based on scRNA-seq data [25]. Consistently, Pairo-Gastineira et al. [24] conducted a GWAS study based on 2244 critically ill COVID-19 patients and highlighted that several genes including *IFNAR2*, *DPP9*, and *OAS1* were significantly associated with severe COVID-19 at a genome-wide significance.

Two primary hypotheses were proposed for the involvement of immune genes in severe COVID-19 susceptibility: whether the severe COVID-19-related risk genes associated with defective innate immune responses would induce persistent viral replication and resultant high viral loads, and whether an exaggerated genetically mediated cytokine production contributes to the hyper-inflammation and poor outcome among severe COVID-19. However, the effects of these genetic determinants on the peripheral immune cells for severe COVID-19 remain largely unknown. In view of a purely genetic study or single-cell sequencing study cannot address this critical question, we here leveraged comprehensive computational methods to combine a large-scale GWAS summary dataset with scRNA-seq data for identifying host genetics that influence immune cell subpopulations involved in the etiology of severe COVID-19.

## Methods

### Single-cell RNA-seq data on severe COVID-19

In this study, we downloaded four independent scRNA-seq datasets on COVID-19 in PBMC and BALF from the ArrayExpress database (dataset #1, the accession number is E-MTAB-9357 from Su et al. study [10]) and the Gene Expression Omnibus (GEO) database (dataset #2, the accession number is GSE149689 from Lee et al. study [18]; dataset #3, the accession number is GSE150861 from Guo et al. study [11]; and dataset #4, the accession number is GSE158055 [12]). The first dataset contained 270 peripheral blood samples including 254 samples with different COVID-19 severity (i.e., mild *N* = 109, moderate *N* = 102, and severe *N* = 50) and 16 healthy controls for scRNA-seq analysis. There were eight patients in dataset #2 with COVID-19 of varying clinical severity, including asymptomatic, mild, and severe, and four healthy controls with PBMCs. Dataset #3 included five peripheral blood samples collected from two severe COVID-19 patients at three different time points during tocilizumab treatment, containing two different stages: severe stage and remission stage. Within dataset #4, 12 BALF samples were collected from lung tissues, including three moderate and nine severe patients. For all the datasets, the sample collection process was reviewed and approved by Institutional Review Boards at the institutions where samples were originally collected. As performed in the original studies [10–12, 18], the COVID-19 severity was evaluated by using the World Health Organization (WHO) ordinal scale (WOS), the National Early Warning Score (NEWS), or the Diagnosis and Treatment of COVID-19 (Trial Version 6). Single-cell transcriptomes for these four datasets were gathered using the 10× Genomics scRNA-seq platform [29]. A total of 606,534 cells with 563,856 PBMC cells and 42,678 BALF cells were yielded from 300 samples based on the four independent scRNA-seq datasets. To allow comparison across samples and datasets, we used a common dictionary of gene symbols to annotate genes and these unrecognized symbols were removed.

### Single-cell RNA sequencing data processing

We performed normalization, clustering, and dimensionality reduction; differential expression gene (DEG) analysis; and visualization on these four independent scRNA-seq datasets with the Seurat R package [30]. The *SCTransform* function was used to scale and transform data, and a linear regression model was applied to omit redundant variations caused by cellular complexity (i.e., cells expressed less than 200 genes or more than 2500 genes were removed) or cellular quality (i.e., cells that had UMIs more than 10,000 and expressed reads of mitochondrial genes greater than 10% were removed). The *CellCycleSoring* function was applied to remove the effects of confounding factors. Principal component analysis (PCA) was carried out to extract principal components (PCs) that could explain most of the datasets via using high variable genes. Top 20 PCs were utilized to conduct uniform manifold approximation and projection (UMAP) to embed the dataset into two dimensions. Subsequently, we constructed a shared nearest-neighbor graph (SNN) using the *FindNeighbors* function based on the top 20 PCs and applied a graph-based modularity-optimization algorithm from the Louvain method [31] on this SNN for clustering the dataset with the cluster resolution set to 0.5. We used the *RunHarmony* function with the PCA reduction method from harmony R package [32] to integrate samples to correct batch effects. The *FindConservedMarkers* function in Seurat was implemented to find differential expressed genes for determining cellular identity. Well-defined markers were used to annotate clusters, and uncharacterized clusters in the first round of clustering were extracted to run the second round of clustering.

### GWAS summary data on hospitalized COVID-19

The meta-GWAS summary data on severe COVID-19 round 4 (B2_ALL, Susceptibility [Hospitalized COVID-19 vs. Population]) were downloaded from the official website of the COVID-19 Host Genetic Consortium [22] (https://www.covid19hg.org/; analyzed file named: “COVID19_HGI_B2_ALL_leave_23andme_20201020.txt.gz”; released date of October 4, 2020). There were 7885 hospitalized COVID-19 patients and 961,804 control participants from 21 independent contributing studies. There was an overwhelming majority of participants in these contributing studies with European ancestry (93%). The meta-GWAS summary statistics contained *P* values, Wald statistic, inverse-variance meta-analyzed log odds ratio (OR), and related standard errors. The 1,000 Genomes Project European Phase 3 [33] was used as a panel for pruning. Results from 23&Me cohort GWAS summary statistics were excluded from our current analysis. Genetic variants without RefSNP number in the Human Genome reference builds 37 were filtered out, giving a total of 9,368,170 genetic variants satisfying the major allele frequency (MAF) over 0.0001 and the imputation score of greater than 0.6. We used the *qqman* R package to figure both Manhattan plot and quantile-quantile (QQ) plot, and the Web-based software of *LocusZoom* (http://locuszoom.sph.umich.edu/) [34] to visualize the regional association plots for significant risk loci (see Additional file 1: Supplementary methods).

### Hierarchical clustering analysis

To examine the similarity of the transcriptome profiles between cell types across different COVID-19 severities, we merged the counts of UMI for each cell type according to normal, mild, moderate, and severe COVID-19. In order to normalize gene expression, we divided the counts of UMI for each gene by the counts of total UMI for all genes in each cell type and then multiplied by 100,000, as refer to the method in a previous study [18]. Based on a median expression value of greater than 0.5, we calculated the relative changes in gene expression divided by the median value for each gene. The Pearson correlation coefficient (PPC) of the relative change in gene expression was used for the current hierarchical clustering analysis.

### Gene-based association analysis

To perform a gene-based genetic association analysis of the meta-GWAS summary statistics on severe COVID-19, we leveraged the updated SNP-wise mean model of MAGMA [35]. In this model, MAGMA computes a test statistic:

$$T={\sum}_i^N{Z}_i^2={\boldsymbol{Z}}^T\boldsymbol{Z}$$

where *N* is the number of SNPs mapped in a gene and ${Z}_i=\mathsf{\varphi}\left({p}_i\right)$. Of note, $\mathsf{\varphi}$ is the cumulative normal distribution function and *p*_*i*_ is the marginal *P* value for a given SNP *i*. SNPs belonging to a specific gene were based on whether located in the gene body or within the ± 20 kb upstream or downstream region of the gene. Furthermore, the model assumes ***Z***~MVN(***0***, ***S***) , where ***S*** is the LD matrix of the SNP genotypes. The LD matrix can be diagonalized and hence written as ***S*** = ***QAQ***^*T*^, where ***Q*** is an orthogonal matrix and ***A*** = diag(*λ*_1_, *λ*_2_, …, *λ*_*N*_) with *λ*_*j*_ being the *j*th eigenvalue of ***S***. The 1,000 Genomes Project Phase 3 European Panel [33] was used for calculating the LD information among SNPs extracted from GWAS summary data on COVID-19.***D***~MVN(***0***, ***I***_*K*_) is a random variable, where ***D*** = ***A***^‐***0.5***^***Q***^***T***^***Z***. Then, the sum of squared SNP *Z*-statistics as the following formula:

$$T={\boldsymbol{Z}}^T\boldsymbol{Z}={\left({\boldsymbol{QA}}^{\boldsymbol{0.5}}\boldsymbol{D}\right)}^T{\boldsymbol{QA}}^{\boldsymbol{0.5}}\boldsymbol{D}={\boldsymbol{D}}^T\boldsymbol{AD}={\sum}_{\boldsymbol{i}}^{\boldsymbol{N}}{\lambda}_{\boldsymbol{i}}{D}_i^2$$

with *D*_*i*_~N(0, 1) and ${D}_i^2\sim {\chi}_1^2$. Namely, *T* follows a mixture distribution of independent ${\chi}_1^2$ random variables. A total of 19,138 genes were included in the current analysis. We used the Benjamini-Hochberg false discovery rate (FDR) method, in which a gene with a FDR ≤ 0.05 (*P* ≤ 6.8 × 10^−5^) was interpreted as significant, to adjust for multiple testing.

### Pathway enrichment analysis

We applied the *built-in* functions of MAGMA [35], using the results from GWAS summary statistics as its input, to examine genome-wide enriched biological pathways for severe COVID-19. We calculated competitive *P* values by examining the results that the combined effect of genes within a pathway is significantly greater than the combined effect of all other genes, and 10,000 permutations were used to adjust competitive *P* values. Additionally, we leveraged the over-representation algorithm of the WebGestalt (http://www.webgestalt.org) [36] along with the significant genes as an input list to conduct a pathway enrichment analysis using the KEGG pathway resource [37]. The number of genes in each pathway was set to between 5 and 2000, and the Benjamini-Hochberg FDR was used for multiple correction. To cluster these identified KEGG pathways, we performed a multidimensional scaling (MDS) analysis based on the Jaccard distance method [38, 39] and constructed a pathway-pathway interaction network for these significantly enriched pathways setting the Jaccard distance > 0.1. For the analyzed codes, please refer to the GitHub repository (https://github.com/mayunlong89/COVID19_scRNA [40]).

### Combining GWAS-based genetic signals with eQTL data

To uncover genetically regulatory expression of genes associated with severe COVID-19, we conducted an integrative genomics analysis by using the S-PrediXcan [41] by combining meta-GWAS summary statistics with expression quantitative trait loci (eQTL) data for 49 tissues from the GTEx Project (version 8) [42]. S-PrediXcan mainly uses two linear regression models to analyze the association between predicted gene expression and severe COVID-19:

$$\boldsymbol{Y}={\boldsymbol{\alpha}}_1+{\boldsymbol{X}}_l{\beta}_l+{\boldsymbol{\varepsilon}}_1$$

$$\boldsymbol{Y}={\boldsymbol{\alpha}}_2+{\boldsymbol{G}}_g{\gamma}_g+{\boldsymbol{\varepsilon}}_2$$

where ***α***_1_ and ***α***_2_ are intercepts, ***ε***_1_ and ***ε***_2_ are independent error terms, ***Y*** is the *n*-dimensional vector for *n* individuals, ***X***_*l*_ is the allelic dosage for SNP *l* in *n* individuals, *β*_*l*_ is the effect size of SNP *l*, G_*g*_ = ∑_*i* ∈ *gene*(*g*)_*ω*_*ig*_X_*i*_ is the predicted expression calculated by *ω*_*lg*_ and ***X***_*l*_, in which *ω*_*lg*_ is derived from the GTEx Project, and *γ*_*g*_ is the effect size of G_*g*_. The *Z*-score (Wald-statistic) of the association between predicted gene expression and severe COVID-19 can be transformed as:

$${Z}_g=\frac{{\hat{\gamma}}_g}{\mathrm{se}\left({\hat{\gamma}}_g\right)}\approx {\sum}_{i\in gene(g)}{\omega}_{ig}\frac{{\hat{\sigma}}_i}{{\hat{\sigma}}_g}\frac{{\hat{\beta}}_i}{\mathrm{se}\left({\hat{\beta}}_i\right)}$$

where ${\hat{\sigma}}_g$ is the standard deviation of G_*g*_ and can be calculated from the 1,000 Genomes Project European Phase 3 Panel, ${\hat{\beta}}_l$ is the effect size from GWAS on COVID-19, and ${\hat{\sigma}}_l$ is the standard deviation of ${\hat{\beta}}_l$. S-PrediXcan was run for each of 49 tissues with 659,158 gene-tissue pairs.

Furthermore, to increase the power to discover significant genes whose expression has similar regulations across multi-tissues, we utilized the S-MultiXcan [43] to meta-analyze these results from the above S-PrediXcan analysis. S-MultiXcan fits a linear regression model of severe COVID-19 on predicted expression from multiple tissue models jointly:

$$\mathrm{Y}=\sum \limits_{j=1}^p{\mathrm{T}}_j{g}_j+\mathrm{e}=\mathrm{Tg}+\mathrm{e}$$

where ${\tilde{\mathrm{T}}}_j={\sum}_{i\in gene(j)}{\omega}_i{\mathrm{X}}_i$ is the predicted expression of tissue *j* and T_*j*_ is the standardization of ${\tilde{\mathrm{T}}}_j$ to mean = 0 and standard deviation = 1. *g*_*j*_ is the effect size for the predicted gene expression in tissue *j*, e is an error term with variance ${\sigma}_e^2$, and *p* is the number of included tissues. There were 22,326 genes across 49 GTEx tissues with integrated convergent evidence in S-MultiXcan, and a gene with a value of FDR ≤ 0.05 (*P* ≤ 3.8×10^−5^) is considered to be significant.

### In silico permutation analysis

To explore the concordance of results from both MAGMA analysis (gene set #1: *N* = 944, *P* ≤ 0.05) and S-MultiXcan analysis (gene set #2: *N* =1274, *P* ≤ 0.05), we performed an in silico permutation analysis which consisted 100,000 times (*N*_Total_) random selections [44, 45]. We first calculated the number of overlapped genes between gene sets #1 and #2 (*N*_Observation_ = 302), then employed the total number of genes in S-MultiXcan analysis as background genes (*N*_Background_ = 22,326). By randomly selecting the same number of genes as gene set #2 (*N* = 1274) from the background genes, and after repeating it 100,000 times, we calculated the number of overlapped genes between gene set #1 and the sample we selected each time (*N*_Random_). Finally, we calculated the empirically permuted *P* value using the following formula: *P* = $\frac{\ {N}_{Random}\ge {N}_{Observation}}{N_{Total}}$, and empirical *P* value ≤ 0.05 is considered to be significant.

### Drug-gene interaction analysis

We conducted a drug-gene interaction analysis for identified genetics-risk genes by using protein-chemical interactions in the context of STRING-based PPI networks [46] and STITCH-based drug annotation information (v5.0, http://stitch.embl.de/) [47]. Only experimentally validated gene-drug interactions with ranked confidence score were selected for constructing a drug-gene interaction network. To examine the potential therapeutic effects of highly expressed genes in each immune cell, we conducted an enrichment analysis of 43 druggable categories based on the DGIdb database (https://www.dgidb.org/druggable_gene_categories) [48]. Additionally, we collected 1263 human druggable proteins, which are therapeutic targets of clinical stage or approved drugs, from a previous study [26]. Among them, 704 proteins are targets for potential COVID-19-relevant drugs based on registers of clinical trials for COVID-19, approved immunomodulatory/anticoagulant drugs, or have biological functions associated with SARS-CoV-2 infection.

### Integrated analysis of GWAS summary statistics and scRNA-seq data

To identify genetically regulatory-related peripheral immune cells for severe COVID-19, we implemented the RolyPoly algorithm [49] to incorporate GWAS summary statistics with scRNA-seq data. Let *g*(*i*) stand for the gene associated with SNP *i*, *S*_*j*_ = {*i* : *g*(*i*) = *j*} be the SNP set with multiple SNPs associated with the gene *j*, and ${\upbeta}_{S_j}$ be a GWAS-based effect-size vector of *S*_*j*_ with a priori assumption that ${\upbeta}_{S_j}\sim \mathrm{MVN}\left(\mathbf{0},{\sigma}_{\mathrm{j}}^2{\boldsymbol{I}}_{\mid {S}_j\mid}\right)$. Following the *prior*, RolyPoly gives a polygenic linear model for ${\upbeta}_{S_j}$:

$${\sigma}_j^2={\gamma}_0+\sum \limits_{i=1}^N{\gamma}_i{\alpha}_{ji}$$

where *γ*_0_ is an intercept term, *α*_*ji*_(*i* = 1, 2, …, *N*) are annotations such as cell-type-specific gene expression, and *γ*_*i*_ are annotation coefficients for *α*_*ji*_. To fit the observed and expected sum squared SNP effect sizes related to each gene by using the method-of-moments estimators, RolyPoly estimates *γ*_*i*_ by the following equation:

$$E\left(\sum \limits_{i\in {S}_j}{\hat{\beta}}_i^2\right)={\sigma}_j^2\mathrm{Tr}\left({\mathrm{R}}_{S_j}^2\right)+\mid {S}_j\mid {\sigma}_e^2{n}^{-1}$$

where ${\mathrm{R}}_{S_j}$ is the LD matrix of *S*_*j*_ and Tr represents the trace of a matrix. Finally, RolyPoly applies the block bootstrap method with 1000 iterations to estimate standard errors ${\hat{\sigma}}_{\gamma_i}$ for calculating a *t*-statistic and corresponding *P* values. The PLINK (v1.90) [50] was used to calculate the LD between SNPs within the 1-Mb window based on the 1,000 Genome Project European Phase 3 panel [33]. We restricted the analysis to SNPs in the autosomes, and any SNPs with MAF ≤ 5% were excluded. The major histocompatibility complex region (Chr6: 25–35 Mbp) was also excluded due to the extensive LD in this region.

### Defining cell state scores

We leveraged cell state score (CTS) to evaluate the immunological degree of individual immune cells expressed a certain predefined expression gene set [12, 14, 51]. The CTSs were initially depended on the average expression of the genes from the predefined gene set in the respective cell. For a given cell *m* and a gene set *k* (GS_*k*_), the cell state score CTS_*k*_ (*m*) was defined as the average relative expression (RE) of the genes in GS_*k*_. Nevertheless, such initial scores may be confounded by cell complexity, as cells with higher complexity have more genes identified and consequently would be expected to obtain higher cell state scores for any expression gene set. To control for this confounding effect by adding a control gene set (CG_*k*_), we calculate a similar cell state score with the control gene set and subtract it from the initial cell scores: CTS_*k*_(*m*) = average[RE(GS_*k*_,*m*)] − average[RE(CG_*k*_, *m*)]. The control gene set was randomly chosen on the basis of aggregate expression level bins, which obtain a comparable distribution of expression levels and over size to that of the pre-curated gene set The *AddModuleScore* function in Seurat [30] was applied to calculate the CTS with default parameters.

We used the inflammatory and cytokine genes (*N* = 324 genes), cytokine-cytokine receptor interactions (*N* = 294 genes), chemokine signaling pathway (*N* = 189 genes), T cell activation (GO: 0042110), response to interferon alpha (GO: 0035455), response to interferon beta (GO: 0035456), leukocyte migration (GO: 0050900), 5 well-defined proliferating markers (*MK167*, *TYMS*, *NKG7*, *IL7R*, and *CCR7*), 6 well-defined exhaustion markers (*LAG3*, *TIGIT*, *PDCD1*, *CTLA4*, *HAVCR2*, and *TOX*), and 12 cytotoxicity-associated genes (*PRF1*, *IFNG*, *GNLY*, *NKG7*, *GZMB*, *GZMA*, *GZMH*, *KLRK1*, *KLRB1*, *KLRD1*, *CTSW*, and *CST7*) to define inflammatory cytokine, chemokine, T cell activation, IFN-α/β response, migration, proliferation, exhaustion, and cytotoxicity score, respectively. These two gene sets of inflammatory and cytokine genes (*N* = 324 genes) and cytokine-cytokine receptor interactions (*N* = 294 genes) were collected to evaluate the level of hyperinflammatory response, which is used to reflect the degree of “cytokine storm” [52].

### Cell-to-cell interaction analysis

To identify potential cellular interactions of *CCR1*^+^ CD16+monocytes and *CXCR6*^+^ memory CD8+T cells with other immune cells, we utilized the CellChat R package [53] for inferring the predicted cell-to-cell communications based on two normalized scRNA-seq datasets (dataset #1 of PBMC and dataset #4 of BALF). CellChat algorithm could examine the significance of ligand-receptor interactions between two cell types depending on the expression of important factors, including stimulatory and inhibitory membrane-bound co-receptors, soluble agonists, and antagonists. The communication probability of a signaling pathway was derived from the sum of probabilities of their ligand-receptor interactions. We only concentrated on the ligand-receptor interactions that were significantly associated with severe COVID-19 compared with normal control.

### Compositional analysis for the proportions of immune cells using the scCODA method

To validate our findings concerning the different percentage of each cell type in PBMCs, we leveraged a Bayesian model of scCODA [54] for re-conducting the compositional single-cell data analysis. The scCODA framework modeled cell type counts with a hierarchical Dirichlet-Multinomial distribution that accounts for the uncertainty in the proportions of immune cell types in PBMCs and the negative correlative bias via collectively modelling of all measured immune cell type proportions rather than individual ones. The Bayesian inference model applied a Logit-normal spike-and-slab prior [55] with a log-link function to estimate these continuous or binary covariates’ effects on immune cell type proportions in PBMCs in a parsimonious fashion. In light of compositional analysis need a reference for identifying compositional changes [56], we used the default parameter in the scCODA: *reference_cell_type* = “automatic”.

### Statistical analysis

The Wilcoxon sum-rank test was used to assess DEGs in mild, moderate, and severe COVID-19 groups compared with normal control [40]. Both the Wilcoxon sum-rank test and scCODA were used for composition data analysis to examine the different proportions of peripheral immune cells across different COVID-19 severity. The Mann-Kendall trend analysis was applied to evaluate the significance of cell state cells with elevated severities of COVID-19. Pathway- and disease-based enrichment analyses used the hypergeometric test to identify remarkable biological pathways and disease terms [36]. The Pearson correlation analysis was used to calculate the correlation coefficient of highly expressed genes in *CCR1*^+^ CD16+monocytes between moderate and severe patients [40]. The paired Student’s *t* test was used to calculate the significance of ligand/receptor interactions of *CCR1*^+^ CD16+monocytes and *CXCR6*^+^ memory CD8+T cells with other immune cells between normal control and severe COVID-19.

## Results

### The computational framework of the current investigation

**Fig. 1:**
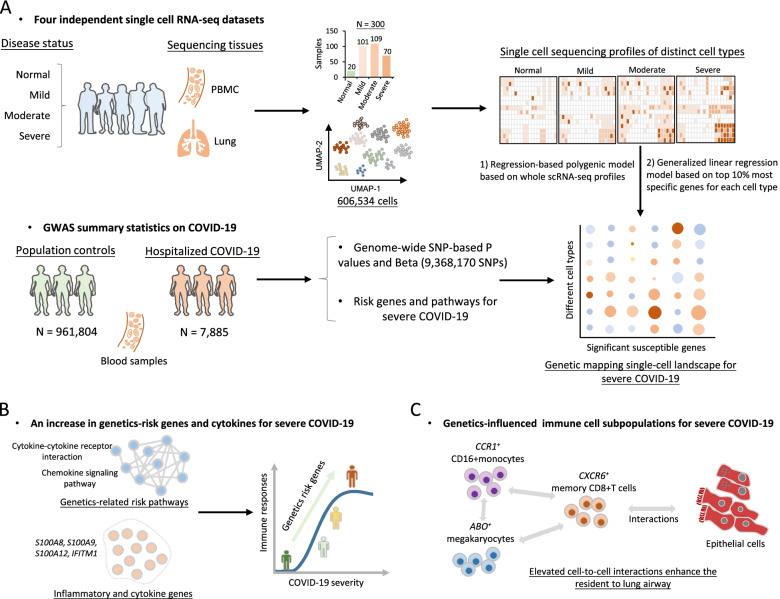
The workflow for this integrative genomic analysis. **A** Combination of single-cell RNA sequencing data and GWAS summary statistics on severe COVID-19 based on two independent methods. One method is the regression-based polygenic model based on whole scRNA-seq profiles, and another is the generalized linear regression model based on the top 10% most specific genes for each cell type. **B** An increase in genetics-risk genes and cytokines for severe COVID-19. **C** Cellular interaction analysis of genetics-influenced immune cell subsets with epithelial cells

As shown in Fig. 1, we devised a computational framework to parse the host genetics-modulated immune cell subpopulations implicated in severe COVID-19. It included three main parts: (1) integrative analysis that combined GWAS summary statistics with scRNA-seq data to genetically map single-cell landscape for severe COVID-19 (Fig. 1A and Additional file 2: Table S1); (2) identifying genetics-risk genes, pathways, and immune cell subpopulations that contributed to cytokine storms among severe patients (Fig. 1B); and (3) uncovering the cellular interactions of genetics-modulated immune cell subsets, as well as their functions with cells in lung tissues (Fig. 1C).

### Identification of immune cell types associated with severe COVID-19

To parse the host genetics-influenced immune responses at a single cellular level in PBMCs for severe COVID-19, we subjected three independent scRNA-seq datasets with 563,856 cells to UMAP based on highly variable genes using the Seurat (see the “Methods” section) [30]. There was identification of 13 distinct clusters unbiased by patients with different severities (Additional file 2: Table S2 and Additional file 3: Fig. S1). We leveraged well-known marker genes to assign these clusters to 13 distinct cell types, including mature B cells, megakaryocytes, naïve B cells, CD34+progenitors, dendritic cells, natural killer (NK) cells, CD14+monocytes, CD16+monocytes, memory CD4+T cells, naïve CD4+T cells, naïve CD8+T cells, memory CD8+T cells, and effector CD8+T cells (Additional file 3: Fig. S2-S3).

While performing the hierarchical clustering analysis on the scRNA-seq profiles, we discovered that cell types were the primary determinants of their clustering, followed by disease severities, indicating both COVID-19 pathology and immune cell types might have crucial roles in altered patterns of immune transcriptome instead of technical artifacts (Additional file 3: Fig. S4). As a vital feature for reflecting the alterations of immune responses, we examined the relative proportions of peripheral immune cells across different COVID-19 groups in comparison with the normal group. The proportions of CD14+monocytes, megakaryocytes, and CD34+progenitors were significantly elevated in moderate and severe patients, whereas the proportions of effector CD8+ T cells, memory CD8+T cells, memory CD4+T cells, naïve CD4+T cells, and NK cells were significantly decreased with the increased severities (Additional file 3: Fig. S5). To provide additional validated evidence, we used an independent method of scCODA to re-perform the compositional data analysis and found the results from scCODA [54] are highly consistent with the above findings from the Wilcoxon sum-rank test (Additional file 3: Fig. S6).

### Identification of genetic risk loci associated with severe COVID-19

**Fig. 2:**
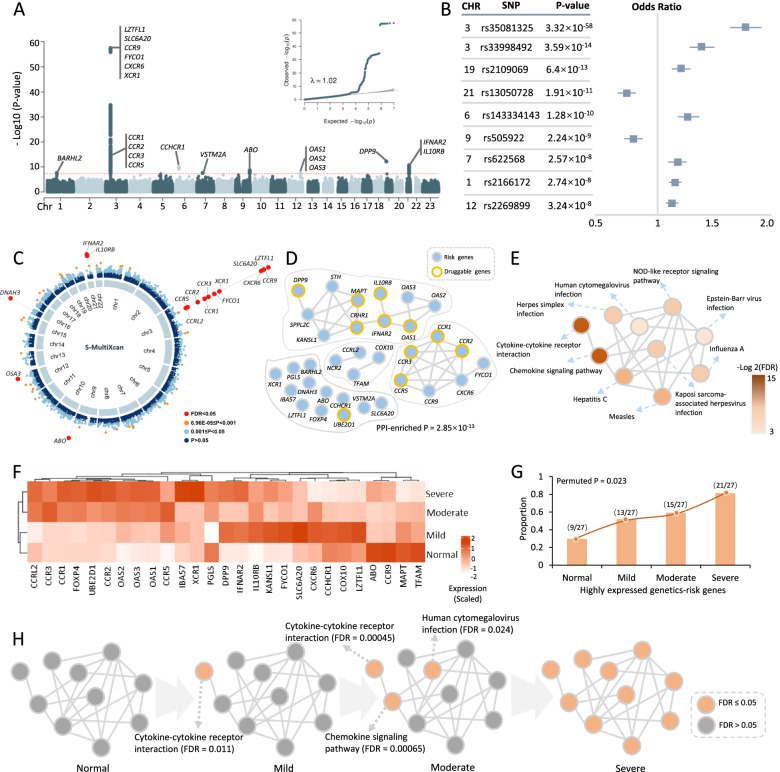
Risk genes and pathways associated with hospitalized COVID-19 from meta-GWAS summary data. **A** Manhattan plot and quantile-quantile (QQ) plot of meta-GWAS analysis highlighting eight risk genetic loci for hospitalized COVID-19. The red horizontal line represents the genome-wide significance threshold of *P* < 5×10^−8^. The genomic inflation factor *λ* = 1.02. **B** Nine index SNPs within eight genomic loci associated with hospitalized COVID-19. The left panel shows the *P* value of each index SNP, and the right panel shows the odds ratio with 95% confidence interval. **C** Circus plot showing the results of the S-MultiXcan-based analysis. The inner ring demonstrates the 22 autosomal chromosomes (Chr1-22). In the outer ring, a circular symbol represents a specific gene and color marks the statistical significance of the gene for hospitalized COVID-19 (red marks FDR < 0.05, orange indicates 6.96×10^−5^ ≤ *P* < 0.001, light blue marks 0.001 ≤ *P* ≤ 0.05, and dark blue indicates *P* > 0.0). **D** PPI network of these 34 identified risk genes based on the STRING database (v11.0, https://string-db.org/). The orange ring represents druggable genes targeted by at least one known drug. **E** Network module constructed by using the Jaccard distance showing the connectivity of 10 significant pathways enriched by 34 risk genes. **F** Heatmap showing the results of hierarchical clustering analysis of 27 risk genes on COVID-19 severity. Seven risk genes did not express in dataset #1, and the expression level of each gene was scaled. **G** The proportion of highly expressed genes among 27 risk genes in normal controls and in the three phases of COVID-19 (mild, moderate, and severe patients). Using 10,000 times of permutation analysis to calculate the significance of the observation (permuted *P* = 0.023). **H** Plot showing an increase of the significantly enriched pathways in the network module with elevated COVID-19 severities. Orange color represents a significantly enriched pathway (FDR ≤ 0.05) and gray color represents a non-significantly enriched pathway (FDR > 0.05)

Through performing a meta-analysis of 21 independent GWAS studies from the COVID-19 Host Genetic Consortium, eight genomic loci were identified to be associated with hospitalization in COVID-19 patients at a genome-wide significant level, including 1p22.2 (rs2166172, *P* = 2.74×10^−8^), 3p21.31 (rs35081325, *P* = 3.32×10^−58^, and rs33998492, *P* = 3.59×10^−14^), 6p21.33 (rs143334143, *P* = 1.28×10^−10^), 7p11.2 (rs622568, *P* = 2.57×10^−8^), 9q34.2 (rs505922, *P* = 2.24×10^−9^), 12q24.13 (rs2269899, *P* = 3.24×10^−8^), 19p13.3 (rs2109069, *P* = 6.4×10^−13^), and 21q22.11 (rs13050728, *P* =1.91×10^−11^) (Fig. 2A, Additional file 2: Table S3 and Additional file 3: Fig. S7). Among these eight loci, three loci, 1p22.2, 6p21.33, and 7p11.2, were newly identified. Consistently, these eight loci were replicated by using a GWAS with critically ill cases of COVID-19 who needed respiratory support in hospital or who died due to the disease (Additional file 2: Table S4). It should be noted that there were two independent genetic association signals (index SNPs: rs35081325 and rs33998492) in the 3p21.31 locus for severe COVID-19 (Fig. 2B and Additional file 3: Fig. S8). Using the Variant2Gene (V2G) algorithm [57], we prioritized *CXCR6* as a candidate causal gene for rs35081325 and causal gene *CCR1* for rs33998492 (see Additional file 1: Supplementary methods).

Furthermore, the index SNP of rs505922 (*P* = 2.24×10^−9^) in the 9q34.2 locus is highly LD with the reported SNP of rs657152 (*R*^2^ = 0.874) [27] and rs8176719 (*R*^2^ = 0.876) [25]. Based on the top-ranked V2G score for rs505922, we prioritized *ABO* as a potential causal gene contributing susceptibility to severe COVID-19. By performing a MAGMA gene-level association analysis, we observed that 25 genes including *CXCR6*, *CCR1*, *IFNAR2*, *IL10RB*, and *OAS1* were significantly associated with severe COVID-19 (FDR < 0.05, Additional file 2: Table S5 and Additional file 3: Fig. S9). GWAS-based pathway enrichment analysis revealed that 19 biological pathways, including cytokine-cytokine receptor interaction, influenza A, and TNF signaling, were significantly associated with hospitalization in COVID-19 patients (Additional file 2: Table S6 and Additional file 3: Fig. S10).

### Integrative analysis of GWAS on severe COVID-19 with GTEx eQTL data

To obtain combined signals from multiple tissues [58], we leveraged S-MultiXcan to meta-analyze the tissue-specific associations from 49 tissues in GTEx, which showed that the genetically predicted expressions of 16 genes were significantly associated with severe COVID-19 (FDR < 0.05, Fig. 2C and Additional file 2: Table S7). Of note, 14 of 16 genes (87.5%) were identified to be significant in MAGMA analysis (Additional file 3: Fig. S11A-B). Through conducting S-PrediXcan analysis of blood and lung tissues that were linked with SARS-CoV-2 infection, we found eight genes whose genetically regulated expression were significantly associated with severe COVID-19 (FDR < 0.05, Additional file 2: Table S8). Using in silico permutation analysis, we further observed that there existed a high consistence among results from MAGMA, S-PrediXcan, and S-MultiXcan analyses (*P* < 1.0×10^−5^, Additional file 3: Fig. S12A-C). The aforementioned multiple genomic analyses identified 34 risk genes that showed supportive evidence of involvement in the etiology of COVID-19 (Additional file 3: Fig. S13A-B).

### Functional characterization of 34 risk genes for severe COVID-19

The result of a network-based enrichment analysis suggested that 22 of 34 risk genes were significantly enriched in a PPI subnetwork (*P* = 2.85×10^−13^, Fig. 2D), which is consistent with the consensus that disease-related genes are more densely connected [59, 60]. To functionally characterize the drug targets of these genes, we conducted a drug-gene interaction analysis and identified 11 genes including *CCR1*, *IFNAR2*, *IL10RB*, and *OAS1* were targeted by at least one known drug (Fig. 2D and Additional file 3: Fig. S14), of which some genes including *CCR1*, *IFNAR2*, and *IL10RB* have been reported to be drug targets for treating severe COVID-19 patients [25, 26]. Furthermore, these 34 genes were significantly enriched in a functional module consisting of 10 biological pathways (Fig. 2E, Additional file 2: Table S9 and Additional file 3: Fig. S15), among which two top-ranked ones being cytokine-cytokine receptor interaction and chemokine signaling pathway (FDR < 0.05). Most of these enriched pathways have been reported to be implicated in COVID-19 [25, 61, 62].

Based on the expression profile of dataset #1, we conducted a hierarchical clustering analysis of these identified risk genes on COVID-19 severity and found that these risk genes predisposed to be highly expressed in severe patients compared to the normal group (permuted *P* = 0.023, Fig. 2F, G). Consistently, the number of significantly enriched pathways was elevated with increased severities (Fig. 2H). Genes in both cytokine-cytokine receptor interaction and chemokine signaling pathways showed significantly high expressions in the early phase of SARS-CoV-2 infection (Fig. 2H), suggesting that these two pathways could play critical roles in the initiation of COVID-19.

### Genetics-influenced peripheral immune cell types for severe COVID-19

**Fig. 3:**
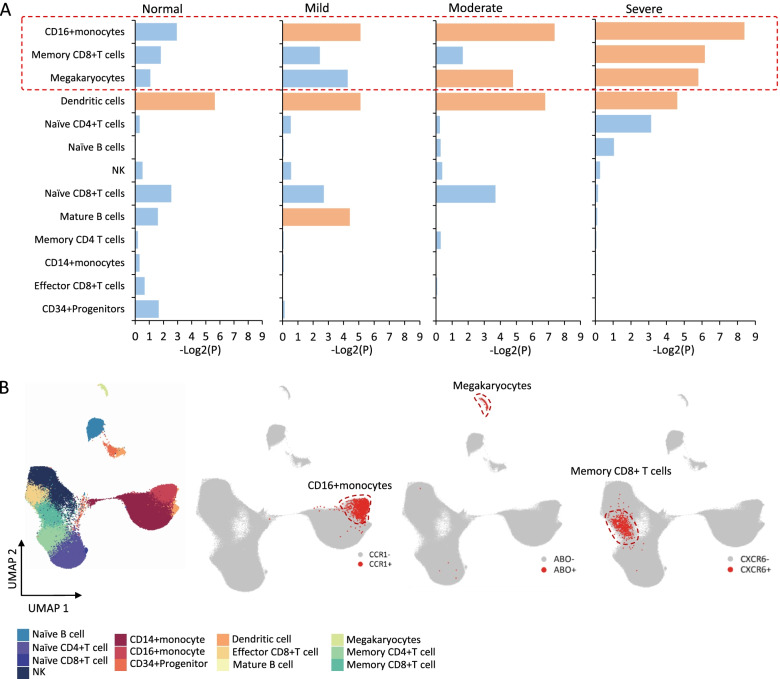
Integrative analysis identifies genetic associations between peripheral immune cells and severe COVID-19. **A** Bar graph showing the results of the combination of scRNA-seq data and GWAS summary statistics on severe COVID-19 based on the RolyPoly among normal controls and patients with different severities (i.e., mild, moderate, and severe). The *y*-axis shows the 13 cell types, and the *x*-axis shows the mean negative log-transformation *P* value (-Log2(*P*)). Orange color indicates a cell type showing a significant association, and light blue represents there is no significant association. **B** UMAP projections of peripheral immune cells colored by annotated cell types. The plot showing the region of CD16+monocytes, megakaryocytes, and memory CD8+T cells. The red dot represents positive gene expressions of *CCR1*^+^, *ABO*^+^, and *CXCR6*^+^, and gray stands for negative cells

To identify genome-wide host genetic components that have effects on peripheral immune cells for severe COVID-19, we first leveraged a regression-based polygenic model [49] to integrate GWAS summary data on severe COVID-19 with single-cell transcriptomic profiles (dataset #1) according to different COVID-19 severities (see the “Methods” section). We found that CD16+monocytes were significantly associated with three phases of COVID-19, mature B cells showed remarkable associations with mild COVID-19, megakaryocytes were significantly associated with moderate and severe COVID-19, and memory CD8+T cells showed significant associations with severe COVID-19 (permuted *P* < 0.05, Fig. 3A). Furthermore, we used a generalized linear regression model [63] to validate these severe COVID-19-associated cell types by conditioning on the 10% most specific genes for each type and consistently found that CD16+monocytes and megakaryocytes showed notable associations with severe COVID-19 (*P* < 0.05, see Additional file 1: Supplementary methods). We found that CD16+ monocytes tended to be associated with severe COVID-19 among patients with younger age, female, and low BMI, whereas memory CD8+ T cells predisposed to be associated with severe COVID-19 among patients with elder age, male, and high BMI. Smoking behaviors contribute a higher risk to the association of both CD16+ monocytes and memory CD8+ T cells with severe COVID-19 (Additional file 3: Fig. S16-S19). Additionally, using the Cell-ID method [64], we found that 34 GWAS-identified genes score of individual cells were higher detected in CD16+ monocytes and memory CD8+T cells (Additional file 3: Fig. S20). These results indicated that CD16+monocytes, megakaryocytes, and memory CD8+T cells were more vulnerable to the influence of genetic components on severe-stage patients.

Based on the specificity algorithm used in MAGMA [63], we noticed that the top specific cell type of *CCR1* was CD16+monocytes, *CXCR6* was most specifically expressed in memory CD8+T cells, and *ABO* was specific to megakaryocytes (Additional file 3: Fig. S21A), recalling that *CXCR6*, *CCR1*, and *ABO* were prioritized to be candidate causal genes for severe COVID-19 based on the V2G score in above genetics-based analysis. Compared with other cell types, *CCR1* was primarily expressed in CD16+monocytes (24.77%), *CXCR6* was mainly expressed in memory CD8+T cells (40.29%), and the *ABO*-expressed cells were highly specific to megakaryocytes (54.63%) (Additional file 2: Table S10 and Additional file 3: Fig. S21B). To gather additional empirical support, we analyzed the combined dataset of both datasets #2 and #3 as a validation and found *CCR1*, *CXCR6*, and *ABO* showed a consistent specificity in the three cell types (Additional file 3: Fig. S22).

Given that the primary goal of the current study was to characterize genetic factors that exert an effect on peripheral immune cell types for severe COVID-19, the majority of our subsequent detailed analyses would be concentrated on three immune cell subpopulations: *CCR1*^+^ CD16+monocytes, *ABO*^+^ megakaryocytes, and *CXCR6*^+^ memory CD8+T cells (Fig. 3B).

### CCR1^+^ CD16+monocytes and ABO^+^ megakaryocytes exacerbating inflammation in severe COVID-19

**Fig. 4:**
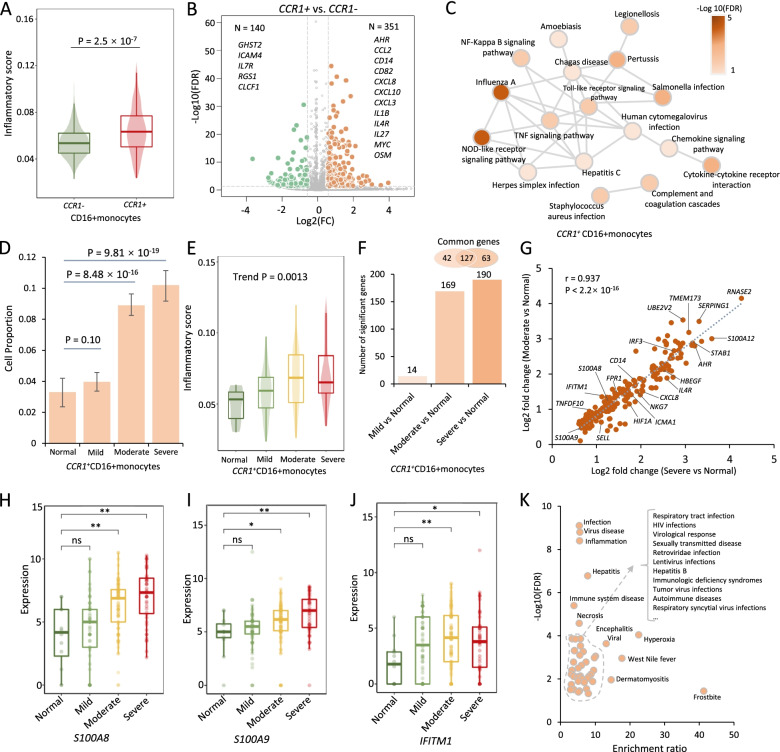
*CCR1*^+^ CD16+momocytes contribute higher risk to cytokine storms among severe COVID-19 patients. **A** Boxplot showing the difference in inflammatory cytokine score between *CCR1*^+^ and *CCR1*^−^ CD16+ monocytes. A two-side Wilcoxon sum-rank test was used. **B** Volcano plot showing differentially expressed genes between *CCR1*^+^ and *CCR1*^−^ CD16+ monocytes. **C** Significantly enriched pathways by 351 highly expressed genes among *CCR1*^+^ CD16+ monocytes. Color legend represents the log-transformed FDR value (-Log10(FDR)). **D** Bar graph showing the proportion of *CCR1*^+^ CD16+ monocytes among normal, mild, moderate, and severe groups. **E** Boxplot showing the inflammatory cytokine score of *CCR1*^+^ CD16+ monocytes among normal, mild, moderate, and severe groups. The Mann-Kendall trend analysis was used. **F** Bar graph showing the differentially up-DEGs among different COVID-19 patients compared with normal controls. Namely, mild COVID-19 vs. normal, moderate COVID-19 vs. normal, and severe COVID-19 vs. normal. Venn plot on top of bar showing the overlapped up-DEGs between moderate and severe patients. **G** The correlation of up-DEGs between moderate and severe patients. Pearson correlation analysis was used to calculate the correlation coefficient and *P* value. **H**–**J** Representative up-DEGs among *CCR1*^+^ CD16+ monocytes showing significantly elevated expressions with increased COVID-19 severities. **H***S100A8*, **I***S100A9*, and **J***IFITM1*. **K** Disease-term enrichment analysis on 190 up-DEGs based on the GLAD4U database. The *y*-axis shows -Log10(FDR), and the *x*-axis shows the enrichment ratio

The accumulating lines of evidence [12, 65] have suggested that subsets of monocytes and megakaryocytes might be the major resources of aggressive hyper-inflammatory response (named as cytokine storm) [52]. We sought to examine whether *CCR1*^***+***^ CD16+monocytes and *ABO*^***+***^ megakaryocytes play more important roles in cytokine storm among severe patients. As for *CCR1*^***+***^ CD16+monocytes, we found that the inflammatory cytokine score was significantly higher than that of *CCR1*^***−***^ CD16+monocytes (*P* = 2.5×10^−7^, Fig. 4A, Additional file 2: Table S11 and Additional file 3: Fig. S24A). Consistently, the combined score of both cytokine-cytokine receptor interaction and chemokine signaling pathway was prominently higher in *CCR1*^***+***^ CD16+monocytes (*P* < 2.2×10^−16^, Additional file 3: Fig. 23A and S24B). These results were validated by using the independent method of Cell-ID [64] (Additional file 1: Supplementary methods and Additional file 3: Fig. S29). Compared with *CCR1*^***−***^ CD16+monocytes, there were 351 significantly highly expressed genes in *CCR1*^***+***^ CD16+monocytes, such as inflammatory and cytokine genes of *IL1B*, *IL27*, *CXCL10*, *CXCL8*, *CD14*, and *OSM* (FDR < 0.05, Fig. 4B and Additional file 2: Table S12), which have been documented to be associated with the inflammatory response and chemotaxis of immune cells among COVID-19 patients [10, 15, 66, 67]. Functionally, 19 KEGG pathways were significantly overrepresented by the 351 highly expressed genes (FDR < 0.05, Fig. 4C and Additional file 2: Table S13), including cytokine-cytokine receptor interaction and chemokine signaling pathway, reminiscing that most of them were identified in above genetics-based pathway analysis. Additionally, these highly expressed genes among *CCR1*^***+***^ CD16+monocytes have a remarkably higher proportion of druggable genes and COVID-19-associated druggable genes (*P* ≤ 0.01, Additional file 2: Table S14 and Additional file 3: Fig. S23B).

The cell percentage of *CCR1*^***+***^ CD16+monocytes showed a notable elevation among moderate and severe patients compared with normal controls (*P* < 0.001), with no significant difference between mild patients and normal controls (*P* = 0.1, Fig. 4D). Furthermore, the inflammatory cytokine scores among *CCR1*^***+***^ CD16+monocytes were significantly elevated with increased severities (Trend *P* = 0.0013, Fig. 4E). In comparison with normal controls, mild, moderate, and severe patients displayed significantly up-regulated expressions (up-DEGs) with 14, 169, and 190 genes respectively (FDR < 0.05, Fig. 4F and Additional file 3: Fig. S23D). Notably, there existed a high correlation between up-DEGs of moderate and severe patients (*r* = 0.937, *P* < 2.2×10^−16^; Fig. 4G), such as *S100A8*, *S100A9*, and *IFITM1* (Fig. 4H–J), indicating a similar expression pattern between moderate and severe patients. Accumulating release of massive amounts of calprotectin (*S100A8/S100A9*) in monocytes contributes to inflammatory response among severe COVID-19 patients [10, 12, 16].

Furthermore, these 190 up-DEGs were significantly enriched in disease terms associated with viral infection and inflammation and 17 functional GO-terms (FDR < 0.05, Fig. 4K, Additional file 2: Tables S15-S16 and Additional file 3: Fig. S23E), including interferon alpha/beta signaling and interferon gamma signaling. These interferon-related genes, including *IRF3*, *IRF2*, *IFI6*, *IFITM1*, *ISG15*, and *ICAM1*, may induce autoinflammatory and autoimmune conditions contributing to the innate immune cells against SARS-CoV-2 infection [68, 69]. Of note, a high proportion of 63.68% among 190 up-DEGs, such as *CXCL8*, *IFITM1*, *S100A8*, and *S100A9*, were annotated into 15 potential druggable gene categories (Additional file 2: Table S17 and Additional file 3: Fig. S23F-L). These results indicate that interferon-related genes among *CCR1*^***+***^ CD16+monocytes have instrumental effects in exacerbating inflammation among severe patients.

In addition, we found that *ABO*^***+***^ megakaryocytes had a significantly higher inflammatory cytokine score than that in *ABO*^***−***^ cells by using two independent methods of *AddModuleScore* in Seurat [30] and Cell-ID [64] (*P* < 0.001, Additional file 3: Fig. S25A-B, S26A-B and S29). Compared with *ABO*^***−***^ megakaryocytes, 424 genes were significantly highly expressed in *ABO*^*+*^ megakaryocytes (FDR < 0.05, Additional file 2: Table S18 and Additional file 3: Fig. S25C). These 424 highly expressed genes were significantly enriched in systemic lupus erythematosus, alcoholism, and platelet activation (FDR < 0.05, Additional file 2: Table S19 and Additional file 3: Fig. S25D). Similar to *CCR1*^***+***^ CD16+monocytes, the cell percentage of *ABO*^***+***^ megakaryocytes was significantly elevated among moderate and severe patients (*P* < 0.01, Additional file 3: Fig. S25E). Among *ABO*^*+*^ megakaryocytes, 20 and 35 up-DEGs were notably associated with moderate and severe patients, respectively (FDR < 0.05, Additional file 3: Fig. S25F-G). There was a highly overlapped rate of these up-DEGs between moderate and severe COVID-19 groups, including *ACP1*, *S100A8*, and *A100A9* (18/20 = 90%, Additional file 3: Fig. S25F-N). These 35 up-DEGs were annotated to 12 druggable gene categories, and significantly enriched in several disease terms, such as shock and thrombocytopenia (Additional file 2: Tables S20-S21 and Additional file 3: Fig. S25H), which were reported to be associated with COVID-19 [70]. Overall, these results suggest that both *CCR1*^***+***^ CD16+monocytes and *ABO*^***+***^ megakaryocytes contribute higher risk to dysfunctional inflammation among severe patients.

### CXCR6^+^ memory CD8+T cells convey risk to severe COVID-19

**Fig. 5:**
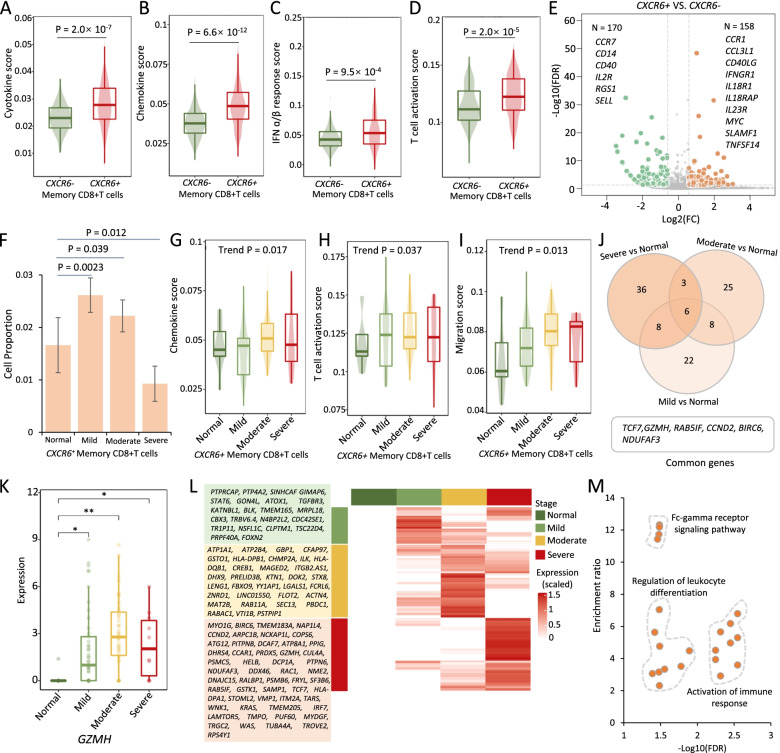
Multi-functionality of *CXCR6*^+^ memory CD8+T cells for severe COVID-19. **A**–**D** Boxplots showing the difference in **A** cytokine score, **B** chemokine score, **C** IFN-ɑ/β response score, and **D** T cell activation score between *CXCR6*^+^ and *CXCR6*^−^ memory CD8+T cells. A two-side Wilcoxon sum-rank test was used. **E** Volcano plot showing differentially expressed genes between *CXCR6*^+^ and *CXCR6*^−^ memory CD8+T cells. **F** Bar graph showing the proportion of *CXCR6*^+^ memory CD8+T cells among normal, mild, moderate, and severe groups. **G**–**I** Boxplots showing the **G** chemokine score, **H** T cell activation score, and **I** migration score of *CXCR6*^+^ memory CD8+T cells among normal, mild, moderate, and severe groups. The Mann-Kendall trend analysis was used. **J** Venn plot showing the overlapped up-DEGs between pairwise comparisons: mild vs. normal, moderate vs. normal, and severe vs. normal. **K** Representative gene of *GZMH* among *CXCR6*^+^ memory CD8+T cells showing significantly elevated expressions with increased COVID-19 severities. **L** Heatmap showing up-DEGs in *CXCR6*^+^ memory CD8+T cells from pairwise comparisons: mild vs. normal, moderate vs. normal, severe vs. normal. The up-DEGs listed in the green panel were from mild vs. normal, in the yellow panel were from moderate vs. normal, and in the orange panel were from severe vs. normal. **M** Scatter plot showing the enriched GO biological processes by 108 up-DEGs among *CXCR6*^+^ memory CD8+T cells. The *x*-axis shows -Log10(FDR), and the *y*-axis shows the enrichment ratio

Earlier studies [10, 71] have indicated that polyfunctional T cells play important roles in dominating the antiviral infection immune response and can release a substantially higher amount of multiple distinct cytokines and chemokines in comparison to other T cells. It is plausible to infer that there exist subsets of memory CD8+T cells predisposing to be multi-functional for against SARS-CoV-2 infection. We calculated several immunological features to evaluate whether *CXCR6*^***+***^ memory CD8+T cells have a higher polyfunctionality than *CXCR6*^***−***^ memory CD8+T cells. Compared with *CXCR6*^***−***^ memory CD8+T cells, we found that scores of cytokine, chemokine, IFN-ɑ/β response, T cell activation, proliferation, and migration were significantly higher among *CXCR6*^***+***^ memory CD8+T cells using both *AddModuleScore* in Seurat [30] and Cell-ID [64] (*P* < 0.05, Fig. 5A–D and Additional file 3: Fig. S27A-C, S28A-G and S29). There were 158 highly expressed genes among *CXCR6*^***+***^ memory CD8+T cells in comparison with *CXCR6*^***−***^ cells (FDR < 0.05, Fig. 5E). These highly expressed genes were significantly enriched in two biological pathways of cytokine-cytokine receptor interaction and inflammatory bowel (FDR < 0.05, Additional file 2: Table S22 and Additional file 3: Fig. S27D). The chemokine signaling pathway showed a suggestive enrichment (*P* < 0.05). These highly expressed genes contained numerous proinflammatory cytokine and chemokine genes, such as *CCR1*, *CCR2*, *CCR5*, *CCR6*, *CCL3L1*, *IFNGR1*, *IL18R1*, *IL23R*, *MYC*, and *TNFSF14*, which may be associated with the activation of memory CD8+T cells.

Furthermore, the cell proportion of *CXCR6*^***+***^ memory CD8+T cells was significantly higher among both mild and moderate COVID-19 than that among the normal group (*P* < 0.05), whereas the cell proportion of *CXCR6*^***+***^ memory CD8+T cells among severe COVID-19 was remarkably lower than that among the normal group (*P* = 0.012, Fig. 5F). Consistently, we found that the scores of chemokine, T cell activation, and migration were increased with the increasing patient severities among *CXCR6*^***+***^ memory CD8+T cells (Trend *P* < 0.05, Fig. 5G–I) and that lower cytotoxicity score and exhaustion score were observed among moderate-to-severe patients (Trend *P* < 0.05, Additional file 3: Fig. S27E-F). Additionally, we found 44, 42, and 53 up-DEGs that were notably associated with mild, moderate, and severe COVID-19, and there were six significant common genes across three phases of COVID-19, including *TCF7*, *GZMH*, *RAB5IF*, *CCND2*, *BIRC6*, and *NDUFAF3* (Fig. 5J–K and Additional file 3: Fig. S27G-N). The gene of *TCF7* was an essential factor in memory CD8+T cell differentiation [72], and *GZMH* was reported to mediate antiviral activity through direct cleavage of viral substrates [73]. These 108 up-DEGs were found to be significantly enriched in 22 functional GO-terms, including Fc-gamma receptor signaling pathway, regulation of leukocyte differentiation, and activation of immune response (Fig. 5L, M and Additional file 2: Table S23). Overall, these results indicated that *CXCR6*^***+***^ memory CD8+T cells have an enhanced propensity to be multi-functional and activated T cells involved in severe COVID-19.

### Elevated interactions of CXCR6^+^ memory CD8+T cells with epithelial cells among severe COVID-19

**Fig. 6:**
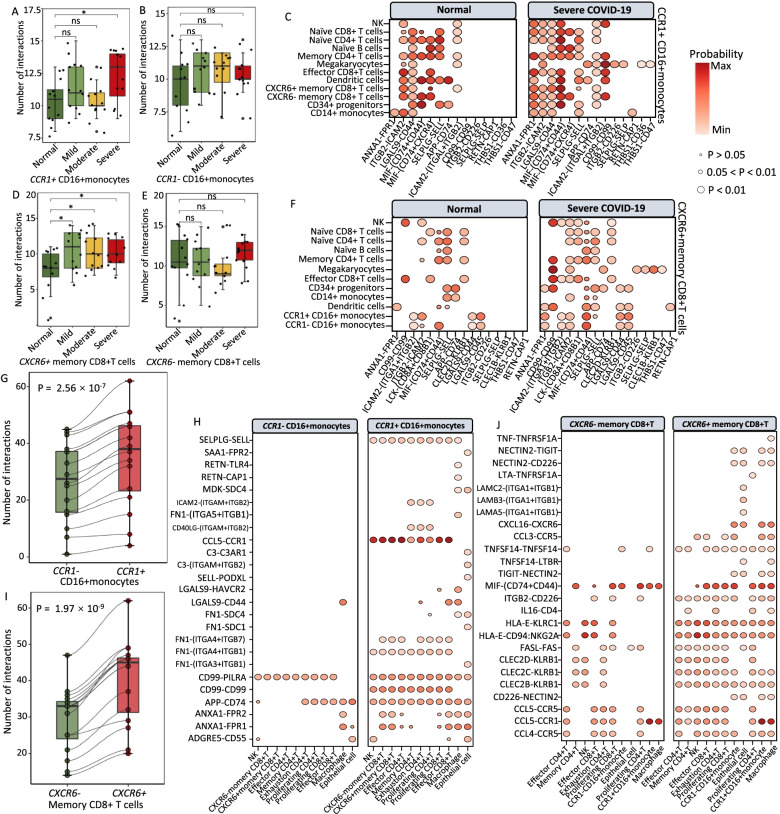
Cell-to-cell interactions of *CCR1*^+^ CD16+momocytes and *CXCR6*^+^ memory CD8+T cells with other cells in PBMC and BALF. **A**, **B** Boxplot showing the number of cellular interactions of **A***CCR1*^+^ CD16+ monocytes and **B***CCR1*^−^ CD16+ monocytes with other immune cells in PBMC between normal controls and patients with increased COVID-19 severities. **C** Predicted cellular interactions of *CCR1*^+^ CD16+ monocytes with other immune cells in PBMC, comparing severe COVID-19 vs. normal control. **D**, **E** Boxplot showing the number of cellular interactions of **D***CXCR6*^+^ memory CD8+T cells and **E***CXCR6*^−^ memory CD8+T cells with other immune cells in PBMC between normal controls and patients with increased COVID-19 severities. **F** Predicted cellular interactions of *CXCR6*^+^ memory CD8+T cells with other immune cells in PBMC, comparing severe COVID-19 vs. normal control. **G** Boxplot showing an increase in cellular interactions with other cells in BALF for *CCR1*^+^ CD16+ monocytes than *CCR1*^−^ CD16+ monocytes. **H** Predicted cellular interactions with other cells in BALF, comparing *CCR1*^+^ CD16+ monocytes with *CCR1*^−^ CD16+ monocytes. **I** Boxplot showing an increase in cellular interactions with other cells in BALF for *CXCR6*^+^ memory CD8+T cells than *CXCR6*^−^ memory CD8+T cells. **J** Predicted cellular interactions with other cells in BALF, comparing *CXCR6*^+^ memory CD8+T cells with *CXCR6*^−^ memory CD8+T cells. The circular size represents the significance of each ligand-receptor axis, and color represents the communication probability

To gain refined insights into *CCR1*^+^ CD16+monocytes and *CXCR6*^+^ memory CD8+T cells, we examined the cellular interactions among cell populations in PBMCs and BALFs according to the COVID-19 disease status using the CellChat algorithm [53]. For *CCR1*^+^ CD16+monocytes in PBMCs, we found an increase in cell-to-cell interactions with other immune cells among severe patients than that in normal controls (*P* < 0.05, Fig. 6A and Additional file 3: Fig. S30). There was no statistical difference in cellular communications of *CCR1*^−^ CD16+monocytes with other cells between normal and COVID-19 patients (*P* > 0.05, Fig. 6B). Compared with normal controls, *CCR1*^+^ CD16+monocytes showed elevated interactions with megakaryocytes, memory CD8+T cells, NK, effector CD8+T cells, and CD14+monocytes among severe patients (Additional file 3: Fig. S30). There were 14 ligand-receptor interactions remarkably dominated among severe patients (Fig. 6C and Additional file 3: Fig. S31A), including *ANXA1-FPR1*, *ITGB2-ICAM2/CD226*, *LGALS9-CD44*, *SELPLG-SELL/SELP*, *APP-CD74*, and *THBS1-CD36/CD47*.

With regard to *CXCR6*^+^ memory CD8+T cells in PBMCs, the predicted cell-to-cell interactions showed an elevation with increased severities of COVID-19 (*P* < 0.05, Fig. 6D). Similar to *CCR1*^−^ CD16+monocytes, we observed no remarked difference of cellular interactions between normal controls and COVID-19 patients among *CXCR6*^−^ memory CD8+T cells (*P* > 0.05, Fig. 6E). Compared with healthy individuals, *CXCR6*^+^ memory CD8+T cells demonstrated higher cellular communications with CD14+monocytes, CD34+progenitors, dendritic cells, effector CD8+T cells, naïve CD8+T cells, memory CD4+T cell, naïve CD4+T cells, NK, and megakaryocytes among severe patients (Additional file 3: Fig. S30). There were 20 elevated cellular interactions of *CXCR6*^+^ memory CD8+T cells with other immune cells among severe patients, including *ANXA1-FPR1*, *THBS1-CD47*, *CD99-CD99*, *ICAM2-*(*ITGAL+ITGB2*), and *ITGB2-ICAM2/CD226* (Fig. 6F and Additional file 3: Fig. S31B). These cell adhesion molecules (*ANXA1* and *ICMA2*), cytokine binding and receptor activity genes (*CD44*, *CD45*, *CD47*, *CD74*, and *THBS1*), and inflammatory genes (*FPR1* and *SELL*) have been reported to be associated with COVID-19 [16, 66, 74, 75].

Among BALF cells, we also observed an increase in cellular interactions of *CCR1*^+^ CD16+monocytes and *CXCR6*^+^ memory CD8+T cells comparing to their corresponding negative cells (*P* < 0.001, Fig. 6G–J and Additional file 3: Fig. S32A), for example, enhanced ligand-receptor axes of *SELPLG-SELL*, *CCL5-CCR1*, *FN1-(ITGA4+ITGB1)*, *CD99-CD99*, and *APP-CD74* among *CCR1*^+^ CD16+monocytes (Fig. 6H), as well as *CXCL16-CXCR6*, *TNFSF14-TNFRSF14*, *ITGB2-CD226*, *CLEC2B/CLEC2C-KLRB1*, and *CCL3/CCL4-CCR5* among *CXCR6*^+^ memory CD8+T cells (Fig. 6J). Notably, there was a 60% increase in cellular interactions between *CCR1*^+^ CD16+monocytes and epithelial cells compared with that of *CCR1*^−^ CD16+monocytes (Additional file 3: Fig. S32B). We also found a 33.33% increase in the interactions between *CXCR6*^+^ memory CD8+T cells and epithelial cells compared with that of *CXCR6*^−^ memory CD8+T cells (Additional file 3: Fig. S32C), such as enhanced ligand-receptor interactions including *TNF-TNFRSF1A*, *CXCL16-CXCR6*, and *CCL3-CCR5*. Previous studies [76, 77] have reported that the *CXCL16-CXCR6* axis modulates the localization of tissue-resident memory CD8+T cells to the lung airways. Overall, these results suggest that the increased cellular interactions of *CXCR6*^+^ memory CD8+T cells with epithelial cells probably enhance the residence of this specific population of T cells to the lung airways for against SARS-CoV-2 infection.

## Discussion

By using large-scale genetics data, we identified eight genomic loci including three novel loci (e.g., 1p22.2, 6p21.33, and 7p11.2) that were significantly associated with severe COVID-19. Other five loci including 3p21.31, 9q34.2, 12q24.13, 19p13.3, and 21q22.11 have been reported to be involved in COVID-19 risk in previous studies [23–28]. Notably, we prioritized 34 risk genes, including potential causal genes of *CXCR6*, *CCR1*, and *ABO*, to be associated with severe COVID-19. The CXC motif chemokine receptor 6 (CXCR6), which is a G protein-coupled receptor with seven transmembrane domains, regulates the partitioning of resident memory T cells by recruiting lung tissue-resident memory CD8+T cells to airways [76]. *CCR1* gene encodes the CC motif chemokine receptor 1 (CCR1) belonging to a member of the beta chemokine receptor family. Several previous GWASs have reported genetic variants in *CCR1* are associated with COVID-19 susceptibility at a genome-wide significant level [25, 27]. For the *ABO* gene, it encodes protein relevant to the ABO blood group system. Both genetic and non-genetic studies [25, 27, 78] have showed the involvement of *ABO* gene in COVID-19 susceptibility, while the *ABO* gene encodes protein that is relevant to the ABO blood group system, and it was also notably associated with several thrombotic and coagulation-related traits including deep vein thrombosis and pulmonary heart disease, which have been reported to be risk factors and sequalae to severe COVID-19 [79, 80].

Understanding the immune responses of monocytes and memory T cells is fundamental to the rational design of innovative and effective strategies to develop better vaccines [81, 82] and contributes to reveal the pathogenesis of severe COVID-19 [12]. Our current analyses reveal that host genetic determinants have a prominent influence on the immune responses of CD16+monocytes, megakaryocytes, and memory CD8+T cells to severe COVID-19. Previous studies [11, 12, 65] showed that the influence caused by monocytes and megakaryocytes in inflammatory storms is noteworthy among severe COVID-19 patients. We found that *CCR1*^*+*^ CD16+monocytes and *ABO*^+^ megakaryocytes showed a significantly increased propensity to cause inflammatory storms among severe patients. The observations suggest highly expressed interferon-related genes among the two cell subsets, including *S100A8*, *S100A9*, *S100A12*, *CD14*, *CXCL8*, *IGSF6*, *IRF3*, *IFI6*, *IFITM1*, and *IFITM3*, contribute to exacerbate inflammation among severe patients. The inflammatory mediator of EN-RAGE encoded by *S100A12* was significantly correlated with COVID-19 [21], and *S100A8*, *S100A9*, *IRF3*, *IFI6*, *IFITM1*, and *IFITM3* have been reported to elicit autoinflammatory and autoimmune conditions in response to SARS-CoV-2 infection [10, 12, 16, 68, 69]. Double-positive CD14+CD16+monocytes reported as tissue-infiltrative cells have a higher potency of antigen presentation and highly expressed proinflammatory cytokines [83, 84]. Additionally, interferons are the mediators in several canonical host antiviral signaling for activating the expression of numerous required molecules of the early response to viral infection [85], and impaired type I interferon activity plays important roles in severe COVID-19 [69]. Our findings described above suggest that *CCR1*^***+***^ CD16+monocytes and *ABO*^+^ megakaryocytes as two functional subsets of myeloid cells convey higher risks to severe COVID-19.

Memory CD8+T cells could elicit immunization that shows enhanced functional features contributing to protect host from viral infectious [82]. After influenza virus infections, memory CD8+T cells reside in the lung for a couple of months and these resident memory T cells are necessary for effective immunity against secondary infection [86]. Among severe COVID-19 patients, we found that *CXCR6*^*+*^ memory CD8+T cells undertook several enhanced functional features, including higher scores of cytokine, chemokine, T cell activation, proliferation, and migration, which suggests *CXCR6*^*+*^ memory CD8+T cells potentially contributing to the protection of SARS-CoV-2 infection. Among these positive *CXCR6*^+^ cells, numerous highly expressed cytokine and chemokine genes, including *CCR1*, *CCR2*, *IFNGR1*, and *MYC*, may work on activating memory T cells. Earlier evidence indicated that MYC was rapidly but temporally induced during the early stage of T cell activation [87]. The *CCR1* plays a pivotal role in the recruitment of effector immune cells to the site of inflammation, and the pharmacologic inhibition of this gene may suppress immune hyper-activation in severe COVID-19 [15]. Memory CD8+T cells obtained the capability of transforming to effector cells by sensing inflammation from monocytes [82]. Thus, inflammatory *CCR1*^*+*^ CD16+monocytes among severe COVID-19 patients potentially accelerate the activation of memory CD8+T cells.

Additionally, we observed a decrease of the cell proportion of *CXCR6*^*+*^ memory CD8+T cells among severe patients. This decrease in peripheral blood among severe patients is probably due to efflux to the site of virally infected lung tissue in answer to ongoing tissue damage. Earlier studies [12, 88] have reported that functional CD8+T cell subsets manifest a notable decrease in the peripheral blood of severe COVID-19 patients. The epithelium is the most vulnerable tissue to be attacked by viral or microbial infection; thus, the presence of resident memory CD8+T cells is imperative for defending the debilitating infections for hosts [86]. In the current study, we found an increase in cellular interactions of *CXCR6*^+^ memory CD8+T cells with epitheliums. Enhanced ligand-receptor interactions including *TNF-TNFSFRSF1A*, *CXCL16-CXCR6*, and *CCL3-CCR5* may contribute to the lung residence of memory CD8+T cells. Previous evidence demonstrated a major role for *CXCL16-CXCR6* interactions in regulating the residence of virus-specific memory CD8+T cells [76, 77]. An earlier study showed a stronger interaction between epithelial and immune cells among severe COVID-19 cases than that among moderate cases [15]. We demonstrated that *CXCR6*^+^ memory CD8+T cells mounted highly effective immune responses to against COVID-19, highlighting the remarkable biological plasticity in subsets of memory CD8+T cells differentiation.

The power of this study is limited by the lack of matched genetic data and scRNA-seq data in each sample for uncovering the genetic effects on immune cells for severe COVID-19. To reduce the influence of this limitation, we adopted a widely used approach by integrating a large-scale GWAS summary statistics with an enormous amount of single-cell sequencing data, as referenced in previous studies [49, 89, 90]. As referenced to previous studies [91–94], we excluded the major histocompatibility complex (MHC) region from all genomic analyses to avoid the confounding of methods by the unusual genetic architecture and extensively high levels of LD at this locus, which could lead to the inflation of identified COVID-19-associated genes and pathways. However, it should be noticed that there might exist un-identified risk genes implicated in severe COVID-19 in this region. Based on our findings suggesting that host genetic components exert regulatory effects on immunological dysregulations for SRAS-CoV-2 infection, more studies are warranted for exploring the genetic modification of peripheral T cells to defend against lethal severe COVID-19.

## Conclusions

In sum, we provide comprehensive insights that host genetic determinants are fundamental in influencing the peripheral immune responses to severe COVID-19. Both *CCR1*^*+*^ CD16+monocytes and *ABO*^*+*^ megakaryocytes contribute higher risk to the dysfunctional inflammatory response among severe patients. *CXCR6*^*+*^ memory CD8+T cells exhibit a notable polyfunctionality including high expression of cytokines and chemokines, as well as enhanced activation and proliferation of T cells in severe COVID-19 patients. Further experiments to parse the molecular mechanism of three cell subpopulations are crucial for understanding the inter-individual variation of the initiation and progression of COVID-19.

## Supplementary Information

**Additional file 1.** Supplementary methods

**Additional file 2: Table S1.** Samples collected from four independent scRNA-seq datasets on COVID-19. **Table S2.** Selected well-known markers used to define cell types in PBMCs. **Table S3.** Significant SNPs associated with severe COVID-19 identified by meta-GWAS analysis. **Table S4.** Replication of these identified loci by using samples with very severe respiratory confirmed COVID-19. **Table S5.** Significant genes associated with severe COVID-19 identified by MAGMA gene-based association analysis. **Table S6.** Significant enriched pathways associated with severe COVID-19 identified from MAGMA-based pathway enrichment analysis. **Table S7.** The 16 significant genes associated with severe COVID-19 identified by S-MultiXcan analysis based on 49 tissues from GTEx consortium. **Table S8.** The eight significant genes associated with severe COVID-19 identified by S-PrediXcan analysis based on lung and blood tissues. **Table S9.** The biological pathways enriched by 34 risk genes associated with severe COVID-19. **Table S10.** The percentage of three severe COVID-19-risk genes expressed in all 13 distinct cell types in PBMCs. **Table S11.** Summary of inflammatory and cytokine-related genes and genes in two identified KEGG pathways. **Table S12.** Highly-expressed inflammatory and cytokine genes among *CCR1+* CD16+monocytes. **Table S13.** Pathway enrichment analysis of 351 highly-expressed genes among *CCR1+* CD16+monocytes. **Table S14.** Druggble proteins collected from the ChEMBL database. **Table S15.** Functional enrichment analysis of 190 up-DEGs associated with severe COVID-19 based on the Reactome database. **Table S16.** Disease-based enrichment analysis of 190 up-DEGs associated with severe COVID-19 among *CCR1+* CD16+monocytes based on the GLAD4U database. **Table S17.** 190 up-DEGs associated with severe COVID-19 among *CCR1+* CD16+monocytes matched in druggable gene categories based on the DGIdb resource. **Table S18.** Highly-expressed inflammatory and cytokine genes among *ABO+* megakaryocytes. **Table S19.** Pathway enrichment analysis of 424 highly-expressed genes among *ABO+* megakaryocytes. **Table S20.** Disease-term enrichment analysis of 35 up-DEGs associated with severe COVID-19 among *ABO+* megakaryocytes based on the GLAD4U database. **Table S21.** 35 up-DEGs significantly associated with severe COVID-19 among ABO+ megakaryocytes matched in druggable gene categories. **Table S22.** Pathway enrichment analysis of 158 highly-expressed genes among CXCR6+ memory CD8+T cells. **Table S23.** GO-terms enrichment analysis of 108 up-DEGs associated with COVID-19 among *CXCR6*^*+*^ memory CD8+T cells.

**Additional file 3: Fig. S1.** UMP projections of cells in PBMCs from normal controls, mild, moderate, and severe COVID-19 patients by using the Seurat R package (dataset #1). **Fig. S2.** Heatmap showing levels of well-known marker genes specific for each cell type in PBMCs. **Fig. S3.** Single-cell transcriptomes of PBMCs from normal controls, mild, moderate, and severe COVID-19 patients. **Fig. S4.** Hierarchical clustering using the PCC of a normalized transcriptome between controls and patients in cell type resolution. **Fig. S5.** Boxplots showing percentages of each cell type for PBMCs in donors from healthy control and COVID-19 patients. **Fig. S6.** scCODA determines the compositional differences of each cell type in PBMCs among donors from healthy control and COVID-19 patients. **Fig. S7.** Regional association plots for severe COVID-19-associated genetic loci based on meta-GWAS summary data. **Fig. S8.** Regional association plots for severe COVID-19-associated genetic loci based on meta-GWAS summary data. **Fig. S9.** Circus plot showing the results of MAGMA-based gene-level association analysis. **Fig. S10.** The 19 biological pathways enriched from the MAGMA-based pathway enrichment analysis. **Fig. S11.** High consistence results between MAGMA and S-MultiXcan analysis. **Fig. S12.** *In silico* permutation analysis of 100,000 times of random selections. **Fig. S13.** Multiple independent approaches identify genetics-relevant risk genes associated with severe COVID-19. **Fig. S14.** Plot of gene-drug interaction analysis for 34 risk genes. **Fig. S15.** The 10 biological pathways significantly enriched by 34 risk genes based on the KEGG database. **Fig. S16.** Barplots showing the results of RolyPoly among COVID-19 patients stratified by patient’s age. **Fig. S17.** Barplots showing the results of RolyPoly among COVID-19 patients stratified by patient’s sex. **Fig. S18.** Barplots showing the results of RolyPoly among COVID-19 patients stratified by patient’s BMI. **Fig. S19.** Barplots showing the results of RolyPoly COVID-19 patients stratified by patient’s smoking status. **Fig. S20.** Cell-ID-based enrichment in GWAS-identified gene signatures (34 genes) of CD16+monocytes and memory CD8+ T cells using scRNA-seq dataset. **Fig. S21.** Genetics-risk genes influenced three immune cell subsets for severe COVID-19. **Fig. S22.** Dot plot showing the expressed percent of three risk genes of *CXCR6*, *CCR1*, and *ABO* in each peripheral cell type in PBMCs among severe patients based on two scRNA-seq dataset of #2 and #3. **Fig. S23.** *CCR1*^+^ CD16+monocytes showing higher risk to cytokine storms among COVID-19 patients. **Fig. S24.** Boxplots showing the difference of inflammatory cytokine score and pathway activation score of CD16+ monocytes among normal controls and COVID-19 groups. **Fig. S25.** *ABO*^+^ megakaryocytes contribute higher risk to cytokine storms among severe COVID-19 patients. **Fig. S26.** Boxplots showing the difference of inflammatory cytokine score and pathway activation score of and megakaryocytes among normal controls and COVID-19 groups. **Fig. S27.** Evidence showing the multi-functionality of CXCR6^+^ memory CD8+T cells for severe COVID-19. **Fig. S28.** Boxplots showing the different score of several immunological features between *CXCR6*^+^ and *CXCR6*^-^ memory CD8+T cells among normal controls and COVID-19 patients. **Fig. S29.** Cell-ID-based enrichment in functional terms of CD16+monocytes, megakaryocyte, and memory CD8+ T cells using scRNA-seq dataset. **Fig. S30.** Differences in the number of predicated cell-to-cell interactions in PBMCs. **Fig. S31.** Predicted cellular interaction of both *CCR1*^*+*^ CD16+ monocytes and *CXCR6*^*+*^ memory CD8+T cells with other immune cells in PBMCs. **Fig. S32.** Prediction of cell-to-cell interactions of cells in BALFs.

## Data Availability

All the GWAS summary statistics used in this study can be accessed in the official websites (www.covid19hg.org/results) [22]. The GTEx eQTL data (version 8) were downloaded from Zenodo repository (https://zenodo.org/record/3518299#.Xv6Z6igzbgl) [42]. Four scRNA-seq datasets were downloaded from the GEO database (https://www.ncbi.nlm.nih.gov/gds/?term=GSE149689 [18], https://www.ncbi.nlm.nih.gov/geo/query/acc.cgi?acc=GSE158055 [12], and https://www.ncbi.nlm.nih.gov/gds/?term=GSE150861 [11]) and the ArrayExpress database (https://www.ebi.ac.uk/arrayexpress/experiments/E-MTAB-9357) [10]. All analyzed codes for hypergenometric analysis [95], hierarchical clustering analysis [18], in silico permutation analysis [25], S-PrediXcan [41], S-MultiXcan [43], MDS [39], MAGMA [35], RolyPoly [49], scCODA [54], Cell-ID [64], and CellChat [53] analysis in the “Methods” section are available in an online GitHub repository at https://github.com/mayunlong89/COVID19_scRNA [40].

## Abbreviations

COVID-19: Coronavirus disease 2019
SARS-CoV-2: Severe acute respiratory syndrome coronavirus 2
GWAS: Genome-wide association study
scRNA-seq: Single-cell RNA sequencing
PBMCs: Peripheral blood mononuclear cells
BALF: Bronchoalveolar lavage fluid
eQTL: Expression quantitative trait loci
GEO: The Gene Expression Omnibus database
WHO: The World Health Organization
SNP: Single nucleotide polymorphism
OR: Odds ratio
MAF: Minor allele frequency
QQ: Quantile-quantile
MAGMA: Multi-marker Analysis of GenoMic Annotation
LD: Linkage disequilibrium
FDR: False discovery rate
KEGG: The Kyoto Encyclopedia of Genes and Genomes
PPC: The Pearson correlation coefficient
MDS: Multidimensional scaling
OTG: The Open Target Genetics
PPI: Protein-protein interaction
up-DEG: Significantly up-regulated expression gene associated with COVID-19
