# Supplementary material for: Integrating single-cell sequencing data with GWAS summary statistics reveals CD16+monocytes and memory CD8+T cells involved in severe COVID-19
Source: Genome Med. 2022 Feb 17;14:16. doi: 10.1186/s13073-022-01021-1 (PMC8851814; doi:10.1186/s13073-022-01021-1)
Supplement: Supplementary file 1 — Additional file 1. Supplementary methods [file 13073_2022_1021_MOESM1_ESM.pdf]

***Supplementary methods***

## Prioritization of candidate causal genes for eight identified genomic loci

To further explore the causal effects of nine index SNPs in eight genomic loci on severe COVID-19, we performed a prioritization analysis by leveraging the Open Target Genetics (OTG, https://genetics.opentargets.org/) tool [1]. The OTG tool is a web-access integrative resource that aggregates human GWAS summary statistics, including UK Biobank data and NHGRI-EBI GWAS catalog, and functional genomic data, including protein quantitative trait loci data (pQTLs), expression QTL (eQTL) from eQTL Catalogue, eQTLGen and GTEx, and promoter capture hiC (PCHI-C) from 27 different cell types, and DNase I hypersensitive site (DHS)-gene promoter correlation and other datasets from a wide range of cell types and tissues. Based on the Variant2Gene (V2G) algorithm, for the index SNP rs2166172 in 1p22.2, we prioritized *BARHL2* as a candidate causal gene for severe COVID-19. With the highest V2G score, *CCHCR1* was highlighted as candidate causal gene for the index SNP rs143334143 in 6p21.33, *VSTM2A* as candidate causal gene for rs622568 in 7p11.2, *OAS1* as candidate causal gene for rs2269899 in 12q24.13, *DPP9* as candidate causal gene for rs2109069 in 19p13.3, and *IFNAR2* as candidate causal gene for rs13050728 in 21q22.11. The index SNP of rs13050728 ($P = 1.91\times10^{-11}$) in 21q22.11 is highly LD with the reported SNP of rs9976829 ($R^2 = 1$) [2] and rs2236757 ($R^2 = 0.8266$) [3]. The index SNP of rs2269899 in 12q24.13 ($P = 3.24\times10^{-8}$) shows high LD with the reported SNP of rs10735079 ($R^2 = 0.9282$) [3].

## Combination of GWAS summary statistics with scRNA-seq data (dataset #1) by using the MAGMA algorithm

As an independent technical approach to validate genetics-related peripheral immune cells in PBMCs implicated in severe COVID-19 identified from the RolyPoly algorithm, we applied a generalized linear regression (GLR) model in the MAGMA (v1.06, https://ctg.cncr.nl/software/magma) [4, 5]. Conditioning on the 10% most specific genes from the single cell data for each cell type, we examined gene-level genetic associations of severe COVID-19-associated immune cell types by adding these variables as covariates for the linear regression model [5-7]. The specificity of each gene for each cell type was calculated by using the mean gene expression of cell type divided the sum of mean gene expression across all cell types. A window of 50 kb upstream to 50 kb downstream for a given gene coordinates was set to calculate gene-level

association statistics. The 1,000 Genome Project European Phase 3 panel [8] was used as the reference panel for both methods. We restricted the GLR analysis to SNPs in the autosomes, and any SNPs with minor allele frequency < 5% were excluded. The major histocompatibility complex region (Chr6: 25-35 Mbp) was also excluded due to the extensive linkage disequilibrium (LD) in this region.

**Using the Cell-ID method to assess the enrichment in specific gene signatures of individual cells in single cell RNA-seq dataset on severe COVID-19**

To deeply investigate the which individual cells in the scRNA-seq dataset are enriched in the gene lists associated to the GWAS hits (N = 34 genes, Additional file 3: Figure S13B), as well as functional terms (Counts > 10 genes as the Cell-ID default) of inflammatory and cytokine genes (N = 324 genes, Additional file 2: Table S10), cytokine-cytokine receptor interactions (N = 294 genes), chemokine signaling pathway (N = 189 genes), T cell activation (GO: 0042110), response to interferon alpha (GO: 0035455), response to interferon beta (GO: 0035456), leukocyte migration (GO: 0050900) we re-performed the analysis by using the Cell-ID tool [9], a clustering-free multivariate statistical method for the robust extraction of per-cell gene signatures from single cell sequencing data. The Cell-ID conducts a dimensionality reduction of gene expression matrix through multiple correspondence analysis (MCA), a statistical algorithm that offers a simultaneous representation of observation (e.g., cells) and variables (e.g., genes) in low-dimensional space [10, 11]. Both genes and cells are projected in a common orthogonal space. In such space, a gene is closer to a cell, suggesting the gene is more specific to the cell.

## References


1. Ghoussaini M, Mountjoy E, Carmona M, Peat G, Schmidt EM, Hercules A, Fumis L, Miranda A, Carvalho-Silva D, Buniello A, et al: **Open Targets Genetics: systematic identification of trait-associated genes using large-scale genetics and functional genomics.** *Nucleic Acids Res* 2021, **49:**D1311-d1320.
2. Ma Y, Huang Y, Zhao S, Yao Y, Zhang Y, Qu J, Wu N, Su J: **Integrative Genomics Analysis Reveals a 21q22.11 Locus Contributing Risk to COVID-19.** *Hum Mol Genet* 2021.
3. Pairo-Castineira E, Clohisey S, Klaric L, Bretherick AD, Rawlik K, Pasko D, Walker S, Parkinson N, Fourman MH, Russell CD, et al: **Genetic mechanisms of critical illness in COVID-19.** *Nature* 2021, **591:**92-98.
4. de Leeuw CA, Mooij JM, Heskes T, Posthuma D: **MAGMA: generalized gene-set analysis of GWAS data.** *PLoS Comput Biol* 2015, **11:**e1004219.

5. Bryois J, Skene NG, Hansen TF, Kogelman LJA, Watson HJ, Liu Z, Brueggeman L, Breen G, Bulik CM, Arenas E, et al: **Genetic identification of cell types underlying brain complex traits yields insights into the etiology of Parkinson's disease.** *Nat Genet* 2020, **52:**482-493.
6. Skene NG, Bryois J, Bakken TE, Breen G, Crowley JJ, Gaspar HA, Giusti-Rodriguez P, Hodge RD, Miller JA, Muñoz-Manchado AB, et al: **Genetic identification of brain cell types underlying schizophrenia.** *Nat Genet* 2018, **50:**825-833.
7. Sheng X, Guan Y, Ma Z, Wu J, Liu H, Qiu C, Vitale S, Miao Z, Seasock MJ, Palmer M, et al: **Mapping the genetic architecture of human traits to cell types in the kidney identifies mechanisms of disease and potential treatments.** *Nat Genet* 2021, **53:**1322-1333.
8. Auton A, Brooks LD, Durbin RM, Garrison EP, Kang HM, Korbel JO, Marchini JL, McCarthy S, McVean GA, Abecasis GR: **A global reference for human genetic variation.** *Nature* 2015, **526:**68-74.
9. Cortal A, Martignetti L, Six E, Rausell A: **Gene signature extraction and cell identity recognition at the single-cell level with Cell-ID.** *Nat Biotechnol* 2021, **39:**1095-1102.
10. Aşan Z, Greenacre M: **Biplots of fuzzy coded data.** *Fuzzy sets and Systems* 2011, **183:**57-71.
11. Rausell A, Juan D, Pazos F, Valencia A: **Protein interactions and ligand binding: from protein subfamilies to functional specificity.** *Proceedings of the National Academy of Sciences* 2010, **107:**1995-2000.
