# Supplementary material for: Integrating single-cell sequencing data with GWAS summary statistics reveals CD16+monocytes and memory CD8+T cells involved in severe COVID-19
Source: Genome Med. 2022 Feb 17;14:16. doi: 10.1186/s13073-022-01021-1 (PMC8851814; doi:10.1186/s13073-022-01021-1)
Supplement: Supplementary file 2 — Additional file 2: Table S1. Samples collected from four independent scRNA-seq datasets on COVID-19. Table S2. Selected well-known markers used to define cell types in PBMCs. Table S3. Significant SNPs associated with severe COVID-19 identified by meta-GWAS analysis. Table S4. Replication of these identified loci by using samples with very severe respiratory confirmed COVID-19. Table S5. Significant genes associated with severe COVID-19 identified by MAGMA gene-based association analysis. Table S6. Significant enriched pathways associated with severe COVID-19 identified from MAGMA-based pathway enrichment analysis. Table S7. The 16 significant genes associated with severe COVID-19 identified by S-MultiXcan analysis based on 49 tissues from GTEx consortium. Table S8. The eight significant genes associated with severe COVID-19 identified by S-PrediXcan analysis based on lung and blood tissues. Table S9. The biological pathways enriched by 34 risk genes associated with severe COVID-19. Table S10. The percentage of three severe COVID-19-risk genes expressed in all 13 distinct cell types in PBMCs. Table S11. Summary of inflammatory and cytokine-related genes and genes in two identified KEGG pathways. Table S12. Highly-expressed inflammatory and cytokine genes among CCR1+ CD16+monocytes. Table S13. Pathway enrichment analysis of 351 highly-expressed genes among CCR1+ CD16+monocytes. Table S14. Druggble proteins collected from the ChEMBL database. Table S15. Functional enrichment analysis of 190 up-DEGs associated with severe COVID-19 based on the Reactome database. Table S16. Disease-based enrichment analysis of 190 up-DEGs associated with severe COVID-19 among CCR1+ CD16+monocytes based on the GLAD4U database. Table S17. 190 up-DEGs associated with severe COVID-19 among CCR1+ CD16+monocytes matched in druggable gene categories based on the DGIdb resource. Table S18. Highly-expressed inflammatory and cytokine genes among ABO+ megakaryocytes. Table S19. Pathway enrichment [file 13073_2022_1021_MOESM2_ESM.pdf]

Supplementary tables

Table S1: Samples collected from four independent scRNA-seq datasets on COVID-19

Characteristics	Dataset #1 (E-MTAB-9357)	Dataset #2 (GSE149689)	Dataset #3 (GSE150861)	Dataset #4 (GSE158055)	Total samples
Normal controls	16	4	0	0	20
Mild COVID-19	96	5	0	0	101
Moderate COVID-19	106	0	0	3	109
Severe COVID-19	52	6	3	9	70
Remission	0	0	4	0	4

Table S2: Selected well-known markers used to define cell types in PBMCs

Cell types	Markers
CD14+ monocytes	*CD14, CD4, NR4A1*
CD16+ monocytes	*FCGR3A, CX3CR1, CD16, CD4, NR4A1*
Naïve CD4+T cells	*LEF1, CD197, TCF7, CD3D, CD3E, CD4*
Naïve CD8+T cells	*LEF1, CD197, TCF7, CD3D, CD3E, CD8A*
Effector CD4+T cells	*PRDM1, PRF1, GZMB, GNLY, CD3D, CD3E, CD4*
Effector CD8+T cells	*PRDM1, PRF1, GZMB, GNLY, CD3D, CD3E, CD8A*
Memory CD4+T cells	*GZMK, CD69, AQP3, CD3D, CD3E, CD4*
Memory CD8+T cells	*GZMK, CD69, AQP3, CD3D, CD3E, CD8A*
Exhaustion CD4+T cells	*LAG3, TIGIT, CD279,PDCD1, TIM3, CD3D, CD3E, CD4*
Exhaustion CD8+T cells	*LAG3, TIGIT, CD279, PDCD1, TIM3, CD3D, CD3E, CD8A*
Proliferating CD4+T cells	*MK167, TYMS, CD3D, CD3E, CD4*
Proliferating CD8+T cells	*MK167, TYMS, CD3D, CD3E, CD8A*
Regulatory CD4+T cells	*FOXP3, CD3D, CD3E, CD4*
Regulatory CD8+T cells	*FOXP3, CD3D, CD3E, CD8A*
NK	*FCGR3A, CD16, TRGC1, NKG7,NCAM1,CD38,CD62L,GZMB,CD56*
B	*CD79A, MS4A1, CD19*
Plasma B	*CD79A, CD38*
Naïve B	*IGHD, FCER2*
mDC	*CD14, CD1C*
pDC	*CLEC4A, CD123*
Megakaryocyte	*PPBP*
Progenitor	*CD34, CD38*
DC	*FCER1A, IL3RA, CD1C, CD141*

Table S3: Significant SNPs associated with severe COVID-19 identified by meta-GWAS analysis

SNP	CHR	POS	Loci	ATL	OR (95% CI)	Meta P-value
rs35081325	3	45489921	3p21.31	T	1.81 (1.788-1.853)	3.32E-58
rs33998492	3	46273598	3p21.31	A	1.36 (1.331-1.379)	3.59E-14
rs2109069	19	4319443	19p13.3	A	1.183 (1.175-1.191)	6.40E-13
rs13050728	21	34215210	21q22.11	C	0.858 (0.851-0.865)	1.914E-11
rs143334143	6	30721426	6p21.33	A	1.273 (1.241-1.273)	1.283E-10
rs505922	9	135749229	9q34.2	T	0.88 (0.875-0.886)	2.24E-09
rs622568	7	54247894	7p11.2	C	1.167 (1.158-1.175)	2.57E-08
rs2166172	1	90808514	1p22.2	C	1.132 (1.126-1.137)	2.74E-08
rs2269899	12	112981956	12q24.13	T	1.132 (1.127-1.138)	3.24E-08

Note: SNP =single nucleotide polymorphism, CHR = chromosome, POS = position, ATL = altered allele, OR = odds ratio, 95% CI = 95% confidence interval, Meta P-value is generated from the Metal tool by meta-analyzing 969,689 samples from 21 independent contributing studies in the COVID-19 Host Genetic Consortium round 4 (B2_ALL, Susceptibility [Hospitalized COVID-19 vs. Population]).

Table S4: Replication of these identified loci by using samples with critically ill cases of COVID-19

SNP	CHR	POS	Loci	ATL	OR (95% CI)	Meta P-value
rs35081325	3	45489921	3p21.31	T	1.88 (1.83-1.937)	2.7E-49
rs33998492	3	46273598	3p21.31	A	1.35 (1.324-1.38)	2.1E-10
rs2109069	19	4319443	19p13.3	A	1.28 (1.267-1.292)	4.2E-21
rs13050728	21	34215210	21q22.11	C	0.82 (0.807-0.828)	1.3E-14
rs143334143	6	30721426	6p21.33	A	1.32 (1.295-1.337)	4.97E-12
rs505922	9	135749229	9q34.2	T	0.903 (0.898-0.908)	4.95E-5
rs622568	7	54247894	7p11.2	C	1.21 (1.198-1.223)	1.42E-08
rs2166172	1	90808514	1p22.2	C	1.07 (1.067-1.074)	6.86E-03
rs2269899	12	112981956	12q24.13	T	1.21 (1.199-1.218)	3.3E-13

Note: SNP =single nucleotide polymorphism, CHR = chromosome, POS = position, ATL = altered allele, OR = odds ratio, 95% CI = 95% confidence interval, Meta P-value is generated from the Metal tool by meta-analyzing 714,592 GWAS samples (5,582 critically ill cases of COVID-19 vs. 709,010 population controls) from 18 independent contributing studies (COVID-19 Host Genetic Consortium round 5, file name: COVID19_HGI_A2_ALL_leave_23andme_20210107.txt). These critically ill cases of COVID-19 were defined as those individuals who needed respiratory support in hospital or who died due to the COVID-19 disease.

Table S5: Significant genes associated with severe COVID-19 identified by MAGMA gene-based association analysis

Gene name	CHR	Start POS	Stop POS	Number of SNPs	Z score	P value	FDR
LZTFL1	chr3	45844808	45977216	314	13.14	1.01E-39	1.93E-35
FYCO1	chr3	45939391	46057316	331	11.15	3.67E-29	3.51E-25
XCR1	chr3	46042291	46088979	165	10.28	4.36E-25	2.78E-21
CXCR6	chr3	45964973	46009845	120	9.91	1.86E-23	8.90E-20
CCR9	chr3	45907996	45964667	152	9.19	2.01E-20	7.69E-17
SLC6A20	chr3	45776941	45858039	208	7.33	1.19E-13	3.80E-10
CCR3	chr3	46263872	46328197	214	7.19	3.35E-13	9.16E-10
CCR1	chr3	46223200	46269832	177	7.00	1.30E-12	3.11E-09
IFNAR2	chr21	34582231	34656831	221	6.83	4.21E-12	8.95E-09
CCR2	chr3	46375235	46422413	136	6.53	3.26E-11	6.24E-08
CCR5	chr3	46391633	46437697	133	5.69	6.28E-09	1.01E-05
OAS3	chr12	113356249	113431056	265	5.69	6.35E-09	1.01E-05
OAS1	chr12	113324739	113377712	157	5.28	6.57E-08	9.67E-05
SPPL2C	chr17	43902256	43944438	281	4.63	1.81E-06	2.47E-03
CRHR1	chr17	43677710	43933194	1203	4.59	2.24E-06	2.86E-03
STH	chr17	44056616	44097060	225	4.57	2.43E-06	2.91E-03
MAPT	chr17	43951702	44125700	901	4.41	5.09E-06	5.73E-03
UBE2D1	chr10	60074739	60150513	188	4.40	5.44E-06	5.78E-03
KANSL1	chr17	44087282	44322740	941	4.20	1.35E-05	1.36E-02
TFAM	chr10	60124903	60178990	145	4.02	2.94E-05	2.81E-02
CCRL2	chr3	46428721	46474488	195	3.99	3.33E-05	3.03E-02
IBA57	chr1	228333429	228389958	118	3.98	3.52E-05	3.06E-02
IL10RB	chr21	34618665	34689539	230	3.92	4.36E-05	3.63E-02
COX10	chr17	13952719	14131996	549	3.87	5.37E-05	4.28E-02
NCR2	chr6	41283528	41338625	328	3.83	6.32E-05	4.84E-02

Note: SNP =single nucleotide polymorphism, CHR = chromosome, POS = position, FDR = False discovery rate.

Table S6: Significant enriched pathways associated with severe COVID-19 identified from MAGMA-based pathway enrichment analysis

ID	KEGG ID	Pathway names	Number of genes	Beta	SE	P value
1	hsa00533	Glycosaminoglycan biosynthesis - keratan sulfate	14	0.66	0.20	5.84E-04
2	hsa05168	Herpes simplex virus 1 infection	183	0.16	0.06	2.13E-03
3	hsa00601	Glycosphingolipid biosynthesis - lacto and neolacto series	27	0.43	0.15	2.69E-03
4	hsa04390	Hippo signaling pathway	153	0.17	0.06	2.87E-03
5	hsa05162	Measles	131	0.19	0.07	3.13E-03
6	hsa05164	Influenza A	167	0.15	0.06	6.36E-03
7	hsa04064	NF-kappa B signaling pathway	92	0.19	0.08	9.44E-03
8	hsa04621	NOD-like receptor signaling pathway	167	0.13	0.06	1.11E-02
9	hsa04625	C-type lectin receptor signaling pathway	104	0.16	0.07	1.13E-02
10	hsa05143	African trypanosomiasis	34	0.28	0.12	1.33E-02
11	hsa05160	Hepatitis C	131	0.14	0.07	1.63E-02
12	hsa04392	Hippo signaling pathway - multiple species	28	0.28	0.13	1.80E-02
13	hsa00603	Glycosphingolipid biosynthesis - globo and isoglobo series	15	0.48	0.23	1.81E-02
14	hsa04137	Mitophagy - animal	64	0.17	0.08	1.87E-02
15	hsa04630	JAK-STAT signaling pathway	158	0.12	0.07	3.50E-02
16	hsa04668	TNF signaling pathway	110	0.13	0.07	3.95E-02
17	hsa00471	D-Glutamine and D-glutamate metabolism	5	0.57	0.34	4.47E-02
18	hsa04060	Cytokine-cytokine receptor interaction	286	0.09	0.05	4.67E-02
19	hsa05132	Salmonella infection	81	0.14	0.09	4.92E-02

Table S7. The 16 significant genes associated with severe COVID-19 identified by S-MultiXcan analysis based on 49 tissues from GTEx consortium

Gene name	CHR	START	STOP	P value	FDR
LZTFL1	chr3	45864808	45957216	1.08E-30	2.41E-26
SLC6A20	chr3	45796941	45838039	2.19E-29	2.44E-25
CCR9	chr3	45927996	45944667	4.16E-29	3.10E-25
CXCR6	chr3	45984973	45989845	1.56E-28	8.71E-25
XCR1	chr3	46062291	46068979	4.20E-16	1.88E-12
FYCO1	chr3	45959391	46037316	1.53E-14	5.69E-11
CCR3	chr3	46283872	46308197	8.15E-13	2.60E-09
CCR1	chr3	46243200	46249832	1.13E-11	3.15E-08
DNAH3	chr16	20944476	21170762	4.44E-10	1.10E-06
CCR2	chr3	46395235	46402413	1.16E-09	2.59E-06
ABO	chr9	136130563	136150630	1.36E-07	2.76E-04
IFNAR2	chr21	34602231	34636831	3.28E-07	6.10E-04
IL10RB	chr21	34638665	34669539	8.28E-07	1.42E-03
CCR5	chr3	46411633	46417697	1.22E-06	1.95E-03
OAS3	chr12	113376249	113411056	2.83E-06	4.21E-03
CCRL2	chr3	46448721	46454488	4.61E-06	6.43E-03

Note: CHR = chromosome, START = start position on chromosome, STOP = stop position on chromosome, FDR = False discovery rate.

Table S8: The eight significant genes associated with severe COVID-19 identified by S-PrediXcan analysis based on lung and blood tissues

Gene name	CHR	START	STOP	Z score	P value	FDR	Tissue
CCR9	chr3	45927996	45944667	11.69	1.47E-31	1.79E-27	Blood
PGLS	chr19	17622278	17632097	-4.64	3.43E-06	2.09E-02	Blood
CXCR6	chr3	45984973	45989845	-10.55	5.10E-26	7.41E-22	Lung
CCR5	chr3	46411633	46417697	-5.46	4.85E-08	3.52E-04	Lung
FOXP4	chr6	41514164	41570122	4.88	1.08E-06	5.23E-03	Lung
IL10RB	chr21	34638665	34669539	4.74	2.19E-06	7.95E-03	Lung
FYCO1	chr3	45959391	46037316	4.65	3.35E-06	8.45E-03	Lung
ABO	chr9	136130563	136150630	4.64	3.49E-06	8.45E-03	Lung

Note: CHR = chromosome, START = start position on chromosome, STOP = stop position on chromosome, FDR = False discovery rate.

Table S9: The biological pathways enriched by 34 risk genes associated with severe COVID-19

ID	KEGG ID	Pathway names	Number of genes	Ratio	P Value	FDR
1	hsa04060	Cytokine-cytokine receptor interaction	294	10.89	3.89E-08	1.27E-05
2	hsa04062	Chemokine signaling pathway	189	13.17	5.12E-07	8.35E-05
3	hsa05160	Hepatitis C	131	10.86	4.29E-04	3.60E-02
4	hsa05162	Measles	132	10.78	4.42E-04	3.60E-02
5	hsa04621	NOD-like receptor signaling pathway	168	8.47	1.10E-03	6.36E-02
6	hsa05164	Influenza A	171	8.32	1.17E-03	6.36E-02
7	hsa05168	Herpes simplex infection	185	7.69	1.57E-03	6.51E-02
8	hsa05167	Kaposi sarcoma-associated herpesvirus infection	186	7.65	1.60E-03	6.51E-02
9	hsa05169	Epstein-Barr virus infection	201	7.08	2.13E-03	0.077
10	hsa05163	Human cytomegalovirus infection	225	6.32	3.20E-03	0.104

Table S10: The percentage of three severe COVID-19-risk genes expressed in all 13 distinct cell types in PBMCs

Cell types	*CXCR6*	*CCR1*	*ABO*
Effector CD8+T cells	0.007	0.008	0.002
Memory CD8+T cells	0.021	0.014	0.004
Naive CD8+T cells	0.013	0.01	0.003
Naive CD4+T cells	3.08E-04	0.001	0.012
Memory CD4+T cells	0.004	0.002	0.014
CD14+ monocytes	8.84E-05	0.099	0.002
CD16+ monocytes	2.42E-04	0.079	0.002
NK	3.19E-03	0.012	0.001
Naive B cells	3.27E-04	0.004	0.007
Dendritic cells	1.63E-04	0.051	0.007
Megakaryocytes	1.64E-03	0.004	0.112
CD34+Progenitors	1.17E-03	0.011	0.039
Mature B cells	0	0.024	0

Table S11: Summary of inflammatory and cytokine-related genes and genes in two identified KEGG pathways

Categories	Gene lists	Resources
Inflammatory-related genes	*ABCA1, ABI1, ACVR1B, ACVR2A, ADGRE1, ADM, ADORA2B, ADRM1, AHR, APLNR, AQP9, ATP2A2, ATP2B1, ATP2C1, AXL, BDKRB1, BEST1, BST2, BTG2, C3AR1, C5AR1, CALCRL, CCL17, CCL2, CCL20, CCL22, CCL24, CCL5,CCL7, CCR7, CCRL2, CD14, CD40, CD48, CD55, CD69, CD70,CD82, CDKN1A, CHST2, CLEC5A, CMKLR1, CSF1, CSF3, CSF3R, CX3CL1, CXCL10, CXCL11, CXCL6, CXCL8, CXCL9, CXCR6, CYBB, DCBLD2, EBI3, EDN1, EIF2AK2, EMP3, EREG, F3, FFAR2, FPR1, FZD5, GABBR1, GCH1, GNA15, GNAI3, GP1BA, GPC3, GPR132, GPR183, HAS2, HBEGF, HIF1A, HPN, HRH1, ICAM1, ICAM4, ICOSLG, IFITM1, IFNAR1, IFNGR2, IL10, IL10RA, IL12B, IL15, IL15RA, IL18, IL18R1, IL18RAP, IL1A, IL1B, IL1R1, IL2RB, IL4R, IL6, IL7R, INHBA, IRAK2, IRF1, IRF7, ITGA5, ITGB3, ITGB8, KCNA3, KCNJ2, KCNMB2, KIF1B, KLF6, LAMP3, LCK, LCP2, LDLR, LIF, LPAR1, LTA, LY6E, LYN, MARCO, MEFV, MEP1A, MET, MMP14, MSR1, MXD1, MYC, NAMPT, NDP, NFKB1, NFKBIA, NLRP3, NMI, NMUR1, NOD2, NPFFR2, OLR1, OPRK1, OSM, OSMR, P2RX4, P2RX7, P2RY2, PCDH7, PDE4B, PDPN, PIK3R5, PLAUR, PROK2, PSEN1, PTAFR, PTGER2, PTGER4, PTGIR, PTPRE, PVR, RAF1, RASGRP1, RELA, RGS1, RGS16, RHOG, RIPK2, RNF144B, ROS1, RTP4, SCARF1, SCN1B, SELE, SELENOS, SELL, SEMA4D, SERPINE1, SGMS2, SLAMF1, SLC11A2, SLC1A2, SLC28A2, SLC31A1, SLC31A2, SLC4A4, SLC7A1, SLC7A2, SPHK1, SRI, STAB1, TACR1, TACR3, TAPBP, TIMP1, TLR1, TLR2, TLR3, TNFAIP6, TNFRSF1B, TNFRSF9, TNFSF10, TNFSF15, TNFSF9, TPBG, VIP*	PMID: 33657410
Cytokine-related genes	*IL2, IL7, CSF3, CXCL10, CCL2, CCL3, TNF, TFTN1, IL6*	PMID: 32192578
Cytokine-related genes	*CXCL10, CCL7, IL1RN, CSF1, IFNG, IL6, IL2RA, IL10, IL18, HGF, CXCL9, CSF3, CCL3, CCL27*	Exuberant elevation of IP-10, MCP-3 and IL-1ra during SARS-CoV-2 2 infection is associated with disease severity and fatal outcome, 2020.3, *MedRxiv*
Cytokine-related genes	*TGFB1*	PMID: 32346099
Cytokine-related genes	*IL1B, LTA, IFNG, CSF1, CSF2, LTB, IL6, TNFSF13, IL18, IL2, IL4*	PMID: 32377375
Cytokine-related genes	*IL1B, IL18, IL6, TNF, CCL2, CCL7, CCL12, CXCL8, CCL3, CXCL9, CXCL10, CXCL11*	PMID: 32505227
Cytokine-related genes	*IL1B, IL6, TNF, CCL2, CCL3, CCL4, CCL7, CXCL9, CXCL10, CXCL11, CXCL1, CXCL2, CXCL3, CXCL8, CCL3L1, CCL8, CXCL16*	PMID: 32398875
Cytokine-related genes	*IL6, CXCL8*	PMID: 32434211
Cytokine-related genes	*CSF2, IL6, IFNG*	Pathogenic T-cells and inflammatory monocytes incite inflammatory storms in severe COVID-19 patients, 2020.6, National Science Review

Cytokine-related genes	*IL6, IL10, CXCL8, CXCL10, IFNG, IFNA1*	A consensus Covid-19 immune signature combines immuno-protection with discrete sepsis-like traits associated with poor prognosis., 2020.6, medRxiv
Cytokine-related genes	*CXCL10, CXCL9, CCL2, IL1RN, CCL5, CCL11, TNF, HGF, IFNA2*	PMID: 32669297
Cytokine-related genes	*TNF, IL1B, IL18*	PMID: 32651212
Cytokine-related genes	*TNF, CCL3, CCL4, CCL20, IL1B, IL6, IL10, CXCL2, CXCL3, CXCL8, CXCL9, CCL3L1, CCL4L2*	PMID: 32764665
Cytokine-related genes	*IL6, CXCL10, CCL7, HGF, OSM, TNFSF14, IFNA1, SA100A12, FGF19, CXCL5, CCL4, CCL8, CCL19, CXCL11, CCL3, IL18R1, CSF1, TNF, CCL20, TGFA, IFNB1*	PMID: 32788292
Cytokine-related genes	*TNF*	PMID: 32877699
Cytokine-related genes	*IL6, IL8, TNF, IL1B*	PMID: 32839624
Cytokine-related genes	*IL17C, TNFSF10, FGF7, XCL1, FGF13, LIF, TGFB3, INHBE, CERS1, TXLNA, IFNW1, IL22, XCL2, CCL25, CCL16, CD40LG, IL20, FASLG, TPO, SCYL3, PF4V1, TNFSF8, GDF15, IL1A, VEGFA, GDF7, BMP6, PDGFA, IL21, ABCD-1, ABCD-2, PDGFB, TNFSF4, FAM19A1, HBEGF, PDGFD, IL12RB2, GH1, VEGFB, MIP3B, IL27, PF4, BMP8B, TNFSF12, IL15, SCYL2, SCYL1, TSLP, GDF11, SDF1B, INHBA, PPBP, FGF11, IFNG-AS1, FGF22, VEGFC, CCL18, TNFSF11, IL12A, EBI3, AMH, IL26, IL32, PDGFC, FGF23, IGF1, IL1F11, CCL28, CLCF1, TNFSF9, BMP3, IL24, GDF10, CXCL6, GDF9, IL23A, IL16, CD70, IL5, FGF9, IFNL1, TSC1, FGF2, IL23R, IL1G, SPP1, IL12RB1, BMP4, IL13, TPAR1, TGFB2, FAM19A2, AGIF3, EDA, MIF, TNFSF13B, BMP7, FGF18, CCL23*	KEGG database (PMID: 10592173)
Genes in the KEGG pathway of cytokine-cytokine receptor interaction (hsa04060)	*GDF11, CCL26, CXCL13, CXCR6, TNFSF13B, CCR9, CCL27, EDAR, IL24, IL17F, TNFRSF13C, CCR1, CCR3, CCR4, CCR5, CCR6, CCR7, CCR8, CNTF, CNTFR, ACVR1C, IL17RE, IL31RA, CSF1, CSF1R, CSF2, CSF2RB, CSF3, CSF3R, CSH1, CSH2, IL34, CTF1, IL23R, GDF7, CX3CR1, IFNLR1, EDA, EPO, EPOR, TNFRSF13B, CLCF1, IL17RA, IL27, IL36RN, GDF1, GDF2, MSTN, GDF9, GDF10, AMH, GH1, GH2, AMHR2, GHR, IL36B, IL37, IL36A, IL17C, IL17B, TNFRSF21, BMP10, CCR10, IFNL2, IFNL3, IFNL1, XCR1, CXCR3, CXCL17, CXCL1, CXCL2, CXCL3, IL19, IFNE, IFNA1, IFNA2, IFNA4, IFNA5, IFNA6, IFNA7, IFNA8, IFNA10, IFNA13, IFNA14, IFNA16, IFNA17, IFNA21, IFNAR1, IFNAR2, IFNB1, IFNG, IFNGR1, IFNGR2, IFNW1, BMP8A, FAS, IL1A, IL1B, IL1R1, IL1RAP, IL1RN, IL2, IL2RA, FASLG, IL2RB, IL2RG, IL3, IL4, IL4R, IL5, IL5RA, IL6, IL6R, IL6ST, IL7, IL7R, CXCL8, CXCR1, IL9, CXCR2, IL10, IL10RA, IL10RB, IL11, IL11RA, IL12A, IL12B, IL12RB1, IL12RB2, IL13, IL13RA1, IL13RA2, IL15, IL15RA, IL16, TNFRSF9, IL17A, IL18, INHA, INHBA, INHBB, INHBC, CXCL10, IL31, CCL4L1, GDF6, LEP, LEPR, LIF, LIFR, LTA, LTB, LTBR, CCL3L3, CXCL9, MPL, NGF, NGFR, NODAL, TNFRSF11B, OSM, IL20, IL21R, IL22, TNFRSF12A, ACKR4, IL23A, PF4, PF4V1, IL17D, IL20RA, IL20RB, PPBP, TNFRSF19, IL17RB, IL26, PRL, PRLR, IL36G, CCL28, IFNK, ACKR3, CXCL16, IL22RA1, IL21, EDA2R, TNFRSF17, CCL1, CCL2, CCL3, CCL3L1, CCL4, CCL5, CCL7, CCL8, CCL11, CCL13, CCL14, CCL15, CCL16, CCL17, CCL18, CCL19, CCL20, CCL21, CCL22, CCL23, CCL24, CCL25, CXCL6, CXCL11, CXCL5, XCL1, CX3CL1, CXCL12, CXCR5, IL25, BMP2, BMP3, BMP4, BMP5, BMP6, BMP7, BMP8B, BMPR1A, BMPR1B, BMPR2, XCL2, TGFB1, TGFB2, TGFB3, TGFBR1, TGFBR2, THPO, TNF, TNFRSF1A, TNFRSF1B, TNFSF4, CCR2, TNFRSF4, IL1R2, CXCR4, GDF5, INHBE, IL1F10, IL17RC, RELT, TSLP, TNFSF11, TNFRSF25, TNFSF14, TNFSF13, TNFSF12, TNFSF10, TNFSF9, TNFRSF14, TNFRSF6B, TNFRSF18,*	KEGG database (PMID: 10592173)

	TNFRSF11A, TNFRSF10D, TNFRSF10C, TNFRSF10B, TNFRSF10A, IL18RAP, IL1RL2, IL18R1, TNFSF18, ACVR1, IL33, ACVR1B, IL1RL1, OSMR, ACVR2A, CD4, BMP15, IL32, ACVR2B, CD27, ACVRL1, TNFRSF8, TNFSF8, IL27RA, GDF15, CXCL14, CCL4L2, GDF3, CD40, CD40LG, CD70, TNFSF15	
Genes in the KEGG pathway of chemokine signaling pathway (hsa04062)	*AKT3, RASGRP2, CCL26, VAV3, CXCL13, CXCR6, GNB5, ADCY1, ADCY2, CCR9, CCL27, ADCY3, ADCY5, ADCY6, ADCY7, ADCY8, CHUK, ADCY9, CCR1, CCR3, CCR4, CCR5, CCR6, CCR7, CCR8, GRK7, CRK, CRKL, PIK3R6, CX3CR1, ADRBK1, ADRBK2, DOCK2, ADCY4, AKT1, AKT2, PTK2B, FGR, FOXO3, PLCB1, PIK3R5, SHC2, GNAI1, GNAI2, GNAI3, GNB1, GNB2, GNB3, GNG3, GNG4, GNG5, GNG7, GNG10, GNG11, GNGT1, GNGT2, CCR10, XCR1, CXCR3, GRK4, GRK5, GRK6, GRB2, CXCL1, CXCL2, CXCL3, GSK3A, GSK3B, HCK, HRAS, IKBKB, CXCL8, CXCR1, CXCR2, CXCL10, ITK, JAK2, JAK3, KRAS, RHOA, CCL4L1, SHC4, LYN, ARRB1, ARRB2, CCL3L3, CXCL9, NFKB1, NFKBIA, NFKBIB, NRAS, PAK1, GNG13, PF4, PF4V1, PIK3CA, PIK3CB, PIK3CD, PIK3CG, PIK3R1, PIK3R2, PLCB2, PLCB3, PLCB4, SHC3, GNG2, PPBP, PRKACA, PRKACB, PRKACG, PRKCB, PRKCD, PRKCZ, MAPK1, MAPK3, GNG12, MAP2K1, PARD3, CCL28, BAD, PTK2, PREX1, CXCL16, PXN, RAC1, RAC2, RAF1, RAP1A, RAP1B, GNB4, RELA, ROCK1, CCL1, CCL2, CCL3, CCL3L1, CCL4, CCL5, CCL7, CCL8, CCL11, CCL13, CCL14, CCL15, CCL16, CCL17, CCL18, CCL19, CCL20, CCL21, CCL22, CCL23, CCL24, CCL25, CXCL6, CXCL11, CXCL5, XCL1, CX3CL1, CXCL12, CXCR5, SHC1, NCF1, SOS1, SOS2, SRC, BRAF, STAT1, STAT2, STAT3, STAT5B, XCL2, TIAM1, CCR2, VAV1, VAV2, WAS, CXCR4, PIK3R3, IKBKG, WASL, GNG8, ROCK2, CXCL14, CCL4L2, BCAR1, ELMO1, CDC42*	KEGG database (PMID: 10592173)

Table S12: Highly-expressed inflammatory and cytokine genes among *CCR1*+ CD16+monocytes

Gene	T score	Fold change	P value	FDR
ADM	6.26	1.72	5.17E-10	2.97E-08
AHR	5.16	1.71	2.83E-07	1.07E-05
AQP9	5.70	1.96	1.47E-08	7.07E-07
C3AR1	6.30	1.56	3.99E-10	2.31E-08
CCL3	6.16	1.55	9.43E-10	5.26E-08
CCL3L1	5.57	1.71	3.01E-08	1.37E-06
CCL4L2	3.06	1.92	2.28E-03	2.49E-02
CD14	18.55	2.14	2.02E-68	1.57E-64
CD82	3.98	1.59	7.16E-05	1.43E-03
CSF3R	7.18	1.56	1.16E-12	9.94E-11
CXCL10	4.17	3.36	3.29E-05	7.14E-04
CXCL2	7.12	2.72	1.83E-12	1.53E-10
CXCL3	3.33	3.33	9.03E-04	1.19E-02
CXCL8	5.56	1.52	3.29E-08	1.48E-06
EIF2AK2	7.87	1.95	7.19E-15	7.88E-13
EREG	6.05	2.02	1.92E-09	1.02E-07
FFAR2	5.56	1.92	3.29E-08	1.48E-06
FPR1	10.14	1.53	2.60E-23	5.69E-21
GPR183	2.99	1.69	2.85E-03	2.99E-02
HBEGF	4.55	1.70	5.84E-06	1.57E-04
IL1B	6.66	1.58	3.89E-11	2.65E-09
IL1RN	8.87	2.68	2.54E-18	3.63E-16
IL27	2.90	2.73	3.75E-03	3.67E-02
IL4R	3.29	1.58	1.04E-03	1.33E-02
ITGA5	2.85	1.53	4.39E-03	4.17E-02
LDLR	4.06	1.72	5.11E-05	1.05E-03
MARCO	7.60	1.66	5.50E-14	5.44E-12

MYC	3.12	1.90	1.83E-03	2.11E-02
NLRP3	4.92	1.61	9.51E-07	3.16E-05
OSM	2.92	1.82	3.53E-03	3.51E-02
PTAFR	6.99	1.85	4.44E-12	3.42E-10
RTP4	3.35	1.80	8.32E-04	1.11E-02
SELL	9.16	1.94	1.92E-19	3.18E-17
SPHK1	4.81	2.25	1.71E-06	5.32E-05
STAB1	7.78	2.40	1.56E-14	1.65E-12
TNFAIP6	3.44	3.33	5.94E-04	8.43E-03
CCL2	3.36	5.74	8.08E-04	1.08E-02

Table S13: Pathway enrichment analysis of 351 highly-expressed genes among *CCR1*+ CD16+monocytes.

Pathway name	Gene size	Enrichment ratio	P Value	FDR
NOD-like receptor signaling pathway	168	4.25	4.32E-07	9.07E-05
Influenza A	171	4.17	5.56E-07	9.07E-05
Cytokine-cytokine receptor interaction	294	3.14	1.61E-06	1.75E-04
Hematopoietic cell lineage	97	5.19	2.83E-06	2.31E-04
Pertussis	76	5.52	1.12E-05	7.32E-04
Salmonella infection	86	4.88	3.39E-05	1.84E-03
Legionellosis	55	6.10	4.16E-05	1.94E-03
Staphylococcus aureus infection	56	5.99	4.75E-05	1.94E-03
Complement and coagulation cascades	79	4.78	9.93E-05	3.60E-03
Toll-like receptor signaling pathway	104	4.03	1.73E-04	5.63E-03
TNF signaling pathway	110	3.81	2.74E-04	8.13E-03
NF-kappa B signaling pathway	95	3.98	4.09E-04	1.11E-02
Malaria	49	5.14	1.01E-03	2.52E-02
Hepatitis C	131	3.20	1.10E-03	2.56E-02
Herpes simplex infection	185	2.72	1.50E-03	3.26E-02
Chemokine signaling pathway	189	2.66	1.80E-03	3.67E-02
Amoebiasis	96	3.50	1.98E-03	3.79E-02
Human cytomegalovirus infection	225	2.42	2.71E-03	4.91E-02
Chagas disease (American trypanosomiasis)	102	3.29	2.90E-03	4.97E-02
Phagosome	152	2.76	3.32E-03	5.42E-02
Cytosolic DNA-sensing pathway	63	4.00	3.71E-03	5.77E-02

Measles	132	2.86	4.16E-03	6.17E-02
Transcriptional misregulation in cancer	186	2.48	4.78E-03	6.77E-02
IL-17 signaling pathway	93	3.16	6.57E-03	8.92E-02
Glycosaminoglycan degradation	19	6.63	9.74E-03	1.27E-01
JAK-STAT signaling pathway	162	2.33	1.51E-02	1.90E-01
Bladder cancer	41	4.09	1.59E-02	1.92E-01
Acute myeloid leukemia	66	3.18	2.01E-02	2.19E-01
Inflammatory bowel disease (IBD)	65	3.23	1.90E-02	2.19E-01
Rheumatoid arthritis	90	2.80	2.01E-02	2.19E-01
RIG-I-like receptor signaling pathway	70	3.00	2.53E-02	2.66E-01
Tuberculosis	179	2.11	2.70E-02	2.75E-01
Nicotinate and nicotinamide metabolism	30	4.20	3.37E-02	2.97E-01
PPAR signaling pathway	74	2.84	3.12E-02	2.97E-01
Osteoclast differentiation	128	2.29	3.29E-02	2.97E-01
Leishmaniasis	74	2.84	3.12E-02	2.97E-01
Kaposi sarcoma-associated herpesvirus infection	186	2.03	3.34E-02	2.97E-01
Antifolate resistance	31	4.06	3.67E-02	3.15E-01
Th17 cell differentiation	107	2.35	4.22E-02	3.52E-01
Glycosphingolipid biosynthesis	15	5.59	4.84E-02	3.85E-01

Table S14: Druggble proteins collected from the ChEMBL database

Categories	Protein lists	Resources
1,263 human druggable proteins	*HGNC_SYMBOL, HTR3E, PSMD12, PLK4, PIK3R2, PSMD14, MSTN, PSMA7, XPO1, DCLK1, PLA2G10, PDPK1, CYP26A1, MLNR, MGAM, HCRTR2, CACNA1F, BRD4, NR1I2, GMNN, STK16, TRPA1, DGAT1, PAK3, HTR3B, BMP10, ABCA1, ABHD16A, F8, F9, TNF, IFNA1, IFNG, IGHE, FN1, TTR, CXCL10, CSF2, MAS1, CD74, CD3D, SHBG, TRAV29DV5, IGF1, CYP17A1, IL5, SERPINE1, CYP1A2, IL6, LCK, CDK1, CD2, CEACAM5, CALCA, LIPF, TUBB, CHRM4, SERPINA6, MMP2, PTPRC, MYL3, HCK, CYP3A4, CYP21A2, HTR1A, CD3G, ALOX5, WEE2, CD28, RARB, THRA, MYL2, HSPA8, CHRM1, PIM1, TOP1, TOP2A, DMD, CDK4, IMPDH2, FCGR1A, SRC, MYH6, RARG, ADRB3, FOLR2, SI, INSRR, PKM, TYR, GABRA1, IFNGR1, FOLR1, CD19, ELN, CYP11B1, PNLIP, ENG, FLT1, ADRA2B, GABRB1, GABRG2, CYP11B2, RXRA, ERBB3, GART, CDH3, CA4, MMP8, PPIB, EDNRB, HRH2, BRD2, PSMA1, PSMA3, HSD3B2, CD27, PIK3R1, PSMB8, PSMB9, PSMB4, PSMB6, RXRB, HSD11B1, CTGF, CD40LG, AVPR2, CALCR, SRD5A2, SSTR3, CNR2, TRHR, PTGS2, OPRM1, SCN4A, PDE6B, IFNGR2, THPO, MPL, ADH7, CASR, GRM5, MC3R, AKR1C3, PIK3CA, CA7, NR4A2, KIR2DL1, KIR2DL2, KIR2DL3, PSMC4, TNNT2, MMP13, NOTCH1, AVPR1B, MTNR1A, CSNK1A1, PIK3CG, MAPKAPK2, PSMB3, CLK1, CLK2, GSK3A, AGTR2, CDK7, IRAK1, PSMD7, CCR4, CCR9, BMX, KIF11, EPHB3, LOXL3, PSMC1, RBX1, TNNC1, TUBA4A, TUBB4B, HBB, HBA1, HBA2, PIP4K2B, HAMP, MUC5AC, CDK6, CDK5, CDK17, NFKB2, SCN7A, FKBP4, KMT2A, RELA, NOTCH2, ACVR1, GRIN1, BTK, LTB, PDE4B, PDE4D, MAP4K2, SLC10A2, GRIN2B, PDE3B, FKBP5, PRPF4B, HTR4, NCOA4, IKBKE, IL13RA2, MELK, NR1I3, EBP, SHH, MYLK, KCNJ8, ADRM1, NFE2L2, RPE65, DDB1, BCL2A1, IL17A, CYP2A13, AOC3, MGAM2, HLA-DRB1, MYLK3, SLC22A6, SLC44A4, TMEM97, TUBA1A, MARK2, SV2A, KCNK18, TPH2, PSMA8, SLC22A8, MAPK15, HDAC2, BCL2L2-PABPN1, GHSR, MAP4K1, APH1A, AURKB, SLC46A1, PVRL4, TAAR1, HBEGF, HSD17B10, TSSK1B, HDAC8, FGF23, IL22, HIPK3, CYP3A43, CD248, PDE7B, TNFRSF12A, KCNK9, DLL4, PSENEN, PIM2, HDAC6, GABBR1, GDF2, CA14, NOTCH3, GABRQ, SRPK3, AKT3, HCN4, ANGPTL3, HRH3, SCN10A, ICOS, TNFSF11, TNFSF14, LTA, CD4, MMP3, IL8, PDGFRA, TNFRSF9, DDR1, GLA, NCAM1, HGF, IL6ST, AKR1C1, MPO, IL12B, IL5RA, PSMB11, MYH7B, PSMD11, BMPR1B, CDC7, PIK3CD, STK25, FAAH, CACNA1A, GABRP, KCNK3, RIOK3, TERT, CHEK1, GABRD, FFAR1, IKBKB, AURKA, GAK, NPC1, ANGPT2, HDAC3, GRIN2D, TLR3, KCNN4, PRPF4, GPR39, PSMD3, MAP3K13, DAPK3, MAP3K7, RIPK2, CACNA1G, KCNQ3, KCNQ2, CA12, HCRTR1, PSCA, PDE6D, AKR1B10, NUAK1, GRIN3B, CCNT1, PDE8A, JAK2, ABCC9, RAMP1, CTSV, FZD7, ROCK2, GUCY1B2, NCOR1, ULK1, CA11, RPS6KA5, RPS6KA4, IDH1, TNFSF13, GABBR2, PDE5A, STK10, PRKD3, KCNK2, S1PR2, CACNA1H, PDE8B, TNKS, ESRRB, MAP4K4, GLP2R, S1PR4, PAK4, CHEK2, ADH1B, ADH1C, ALDH1A1, DHFR, GSR, PAH, SOD1, PNP, ABL1, CFD, F12, PLAT, CA1, CA2, SERPINC1, C5, PDGFB, NGF, IGF2, IL1A, IL1B, TRAC, LMNA, TNNC2, FGB, CHRNA1, AFP, GC, ESR1, KLKB1, TRGV3, HMGCR, RAF1, GBA, APOB, NR3C1, TK1, VWF, TUBB4A, TRBV7-9, ERBB2, NTRK1, TP53, AMY2A, CYP1A1, TYMS, ATP1A1, ATP1B1, APP, ALDH2, ITGB3, ITGB2, CYP11A1, IL4, PRKCG, FGF1, ITGB1, PRKCB, INSR, FYN, BCHE, PGR, EIF4E, NPM1, ITGAV, TH, TPO, KLK3, H1F0, ADH1A, FES, CSF1R, CHRNG, ADRB2, CD3E, HSP90AA1, YES1, LYN, IGF1R, CHRM2, ABCB1, NR3C2, HSP90AB1, ADH4, MME, INHBA, ITGA2B, MET, ADRB1, FCGR3A, ITGA5, VIM, STS, CHRM5, ADRA2A, ROS1, FGF2, HMGB1, TACSTD2, FGR, PARP1, LTA4H, ADORA3, CALM1, TUBA3C, CALCB, GAA, AR, RARA, ARAF, BCL2, TGFB3, CYP2D6, MAPT, KIT, THRB, CD37, UMPS, ITGAM, CHRNB1, BCR, FGFR1, VDR, ESRRA, CYP2A6, CYP19A1, CETP, CYP2C9, ADH5, MS4A1, ODC1, ACE, MYL4, MYH7, CCL2, CFTR, ITGA4, ATP1A3, EEF2, SLC5A1, HSD17B1, FDPS, DRD2, IL2RB, BRAF, GLUL, IL9, CSF2RA, PHKG2, MUC1, ALOX15, NPR1, CD44, CTLA4, EPCAM, DPEP1, TSHR, PDE6A, SELE, FER, PRKCA, ITGA2, PRKACA, TYRP1, TPH1, CHRNB2, CR1, PSMC3, ALOX12, ITGB5, SRD5A1, PDE6G, ITGB6, ADRA2C, SDC1, EPOR, TNNI3, AOC1, NFKB1, TYMP, CD22, ALOX5AP, CHRM3, PSMB1, ATP4A, ITGAL, DDC, CYP3A5, IMPDH1, MAG, MAOA, TACR2, S1PR1, CNR1, DRD1, C5AR1, TBXA2R, FGFR2, RYR1, DRD4,*	PMID: 33837377

	DRD5, COMT, IL10, ACHE, FGFR4, FGFR3, PRKACG, PRKACB, LHCGR, PTGS1, GLRA1, RPS6KB1, JAK1, TNFSF4, CPT2, RRM1, FSHR, CMA1, SLC6A2, GABRR1, CCND1, CYP3A7, TBXAS1, PRKCH, CCNE1, CDK2, CXCR1, CXCR2, GUCY2C, ADRA1D, EDNRA, TACR1, PTAFR, PSMA2, PSMA4, ITGB7, DNMT1, IL3RA, MAOB, MAPK3, MARK3, DPP4, PDE4A, PSMA5, PSMB5, HTR1D, HTR1B, HTR2A, ADH6, HTR2C, GABRB3, MAPK1, TNFRSF8, IMPA1, ADORA2A, ADORA2B, EPHA2, EPHA3, EPHA8, TACR3, LTK, CASP1, TYK2, CD6, CCND2, CCND3, WEE1, BDKRB2, SLC6A1, CHRNA5, TSPO, ADORA1, AGTR1, OXTR, SSTR1, SSTR2, CHRNB4, HTR1F, GNRHR, NTSR1, CPS1, RRM2, CD52, SSTR4, SLC5A2, GABRA5, SLC6A4, AKT1, AKT2, ATIC, CCKAR, CCKBR, MC4R, CCR1, CHRNA3, GRK4, HPD, ICAM3, CD70, MC5R, KIF5B, CYP2C19, GUCY1A2, ABCC1, CD80, TTK, GABRA3, EPHX2, HTR7, PTGER1, CRHR1, IL13, SSTR5, ADRA1A, HRH1, ADRA1B, PTGER4, DRD3, SCN1A, SOAT1, BSG, CHKA, PSMC2, MAP2K2, CHRNA7, FLT3, TGFBR1, SCNN1A, PPARG, FDFT1, AVPR1A, GGCX, GRIK1, PLA2G5, CD79B, PSMB10, IL15, PMEL, OPRD1, OPRK1, OPRL1, P2RY2, HNF4A, CSK, IARS, SLC19A1, HTR2B, CCR2, PRKCI, CD86, GRIA1, GRIA2, GRIA3, PIK3CB, MTOR, TEC, TXK, ABL2, FRK, HTT, PTGFR, PTGER3, PTGER2, PTGIR, MCAM, GLP1R, CTSK, GPD2, SYK, TNFRSF4, NAMPT, CHRNA4, TNNT3, MAPK8, MAPK9, RECQL, HTR3A, BDKRB1, GABRA2, GABRB2, GCGR, SCTR, HTR5A, XDH, GRIA4, SLC6A9, GABRA4, RXRG, IFNAR2, PSMD8, CSNK1D, IDH2, SLC9A3, TNNI2, MTNR1B, CDK8, FNTA, FNTB, CSNK1E, PSMB2, PGF, VEGFB, PSEN1, PSEN2, GSK3B, DIO1, NPY4R, HTR6, CDK9, ATP1A2, RAB9A, PDE6C, ATP4B, SCNN1B, SCNN1G, RORC, BLK, CYP2J2, ALDH5A1, CCL11, CCR5, KCNQ1, CLCN2, RPS6KA3, JAK3, PLK1, DAPK1, COL4A4, LIMK2, MAPK12, MAPK10, BLM, CACNA2D1, PRKAA2, ATP1B3, FXYD2, EPHA5, EPHB1, EPHA4, SLC12A3, PSMD4, NR1H2, MTTP, CASP9, GFER, P2RX3, HDAC4, KCNQ4, BACE1, CLDN18, SIK1, KCNK10, SHFM1, PSMA6, CXCR4, TGFB2, PSMC5, PSMC6, ESRRG, PPIA, FKBP1A, KCNJ2, FKBP1B, TUBA1B, CSNK2A1, GABRE, SRPK2, PRKDC, ADAM17, IL13RA1, MAP3K9, HSD11B2, ABAT, CDK16, CACNA1B, MDM2, MYL7, PMP22, MYT1, CACNA1D, MC2R, MC1R, SLC6A3, GUCY1A3, DHODH, GUCY1B3, PRKCE, RHD, GHRHR, MAP2K1, MAP3K10, TOP2B, PPARD, PTH1R, FOLH1, PRKCQ, CHRNE, MST1R, PTK2, PRKCZ, PRKCD, CHRNB3, SLC18A2, PPAT, AOX1, TYRO3, CHRND, BCL2L1, MCL1, PPARA, TNK2, CYP24A1, PPP3CA, PDE4C, PPID, ITK, DMPK, ABCC8, KCNMA1, KCNH2, MAP3K12, GRIN2A, DPYD, FAP, GRIK2, GRIK3, STK4, PLA2G7, PRKAA1, NR1H3, MAP2K5, PAK2, STK3, PSMD2, MAP3K1, PRKG2, PTGDR, GPR17, MSLN, IKZF1, ROCK1, FZD5, TNK1, MADCAM1, BIRC3, BIRC2, TUBB3, ATR, HDAC1, CAMK2G, CAMK2D, NAE1, CUL4A, SLC12A1, DYRK1A, TPBG, CACNA1S, ACVR2B, ATP1A4, BMPR2, PTK6, TUBB2A, CACNA1C, PDE7A, PDE6H, PRKG1, IL18, KEAP1, PTK2B, FZD2, GRM2, PDE3A, SCN5A, SQLE, KCNJ11, GRM3, GRIN2C, PSMD6, BRD3, PDCD1, PDK1, PDK2, PDK3, PRKD1, ERBB4, RPS6KA2, DHCR24, SF3B3, RYR3, RPS6KA1, TESK1, LTB4R, MAPK11, NPY5R, CHRNA2, CHRNA6, STK11, GRK1, SCN9A, GRIK4, CCDC6, GABRA6, GRIK5, PKN1, PKN2, MAPK14, MAP3K11, CALCRL, NTRK2, SMN2, PDK4, HIF1A, UGCG, CA9, PHKG1, DDR2, CYP51A1, TXNRD1, AAK1, TUBB8, PDCD4, MAP3K19, BRDT, TNNI3K, LRRK2, ANO1, CD276, TUBA3E, ULK3, NPSR1, HTR3D, TRPM8, DPP9, PIM3, MYLK4, HIPK1, IFNLR1, CAMK1D, MAP4K3, MAPKAPK5, ULK2, GABRG1, CA13, ABHD12, MINK1, DAGLB, HIPK4, TRPV1, TRPV3, UBA3, GRIN3A, STK35, HCAR2, GPBAR1, GRK7, CLEC4C, HDAC7, APH1B, HTR3C, RXFP2, MUC16, PIK3R5, CPT1B, NCSTN, PIK3R3, ESR2, RORB, BCL2L2, SLCO2A1, BHMT, TSLP, TOP1MT, HDAC10, HDAC11, LINGO1, SLC47A1, LOXL4, EGLN2, NR1H4, CAMKK2, SLC22A12, SRPK1, CRBN, CSF3R, SCN2A, PSMB7, PSMD1, S1PR3, GPER, P2RX7, PKMYT1, MAP3K5, SIGMAR1, PIP5K1A, MAP3K3, GPA33, SLC29A1, EPAS1, TSG101, SMO, GABRG3, SOST, VKORC1, TUBA1C, RIOK1, TUBB6, ABHD6, TUBB2B, RIOK2, EBPL, ACE2, STK33, DCLK3, EGLN1, CHRNA10, NUAK2, PARP12, SIK2, MYLK2, S1PR5, P2RY12, SLK, TNKS2, TAOK3, BHMT2, HIPK2, HRH4, SRMS, FZD8, TUBB1, EGLN3, SRD5A3, CLK4, TRPV4, RXFP1, NOD2, EML4, GPR35, PDE11A, BCL2L10, IL23A, RTN4, KCNQ5, TLR9, CYSLTR2, SLC22A11, BMP2K, TDP1, IRAK4, SIRT5, P4HTM, SCN3A, CACNA2D2, SLC5A4, TLR7, MAP3K20, IL20, CISD1, CACNA1I, STK26, RCOR3, EIF2AK4, DHCR7, RPS6KB2, MALT1, STK17A, EPHA6, CHRNA9, PARP2, NPC1L1, TBK1, STEAP1, EGFL7, SCN11A, DAPK2, TNIK, UTS2R, IKZF3, HDAC9, ALK, CELA1, PSMD13, FZD1, TFR2, AURKC, SCN8A, HDAC5, CAMK2A, PDE10A, HPSE, CYSLTR1, CA5B, NISCH, SIK3, MAP3K2, DYRK1B,	

	L3MBTL1, DAGLA, LOXL2, MAP4K5, TNFRSF18, PTGDR2, IRAK3, NCOR2, STK24, PARP3, DNMT3A, MAP3K4, TNFRSF10A, TNFRSF10B, EGFR, REN, ADA, TGFB1, IL2RA, TFRC, MMP1, CD5, MMP7, CSF1, SPP1, IL1R1, TNFRSF1A, IL4R, F2R, CD40, CD38, AXL, CA5A, LTBR, ACVRL1, CD200, SULT1A1, EPHB4, IL10RB, MERTK, CASP8, ANGPT1, NTRK3, PCSK9, CD274, TNFSF13B, F2, PLG, CD14, TLR4, NRP1, MAPK13, ICOSLG, DKK1, ICAM1, FCER2, CA3, RET, GSTP1, PDGFRB, GHR, PTHLH, TDGF1, SELL, ATP1B2, PLA2G2A, MMP9, IDO1, AKR1B1, NQO1, NQO2, CBR1, IFNAR1, CD33, EPHA1, CA6, TNC, EPHB2, CSF2RB, TIE1, FLT4, KDR, SNCA, PTGDS, PRCP, LEPR, VEGFC, IL11RA, GPNMB, RRM2B, LY96, EPHB6, TNFSF12, VEGFA, SELP, MMP12, GFRA1, DLK1, TEK, PDCD1LG2, SLAMF7, F10, MFGE8, IL6R, IL17RA,	
704 human druggable proteins have evidence for involvement in COVID-19	*SIGMAR1, DNMT1, SIRT5, BRD4, BRD2, IL17RA, TLR9, TLR7, ESR1, ESR2, CSNK2A1, RET, FLT3, PIK3CD, TOP2A, PIK3R1, AXL, PIK3CB, PIK3CG, EBP, ALK, PIK3R2, CCNT1, CDK1, ROS1, CDK4, CCND1, CCNE1, CDK2, CCND3, PIK3CA, CDK9, CDK6, CDK5, DHCR24, MAPK14, PIK3R5, PIK3R3, ABL1, LMNA, LYN, CHRM4, CHRM5, ADRA2A, PDGFRB, KIT, BCR, FGFR1, DRD2, PDGFRA, FLT1, ADRA2C, CHRM3, IMPDH1, FGFR2, FGFR4, FGFR3, ADRA1D, HTR2A, HTR2C, HRH1, DRD3, KDR, HTR2B, ABL2, HTR6, SMN1, SMN2, PLK4, GAK, NPC1, JAK2, CACNA1F, ROCK2, EGFR, F10, CA1, CA2, NR3C1, ERBB2, TP53, INSR, LCK, FYN, PGR, EIF4E, CSF1R, YES1, IGF1R, ABCB1, HCK, CYP3A4, FGR, RARA, RARB, CHRM1, VDR, CYP2C9, RARG, NQO2, ADRA2B, ATP4A, DRD1, DRD4, PPIB, SLC6A2, AGTR1, SLC6A4, CYP2C19, HTR7, PTGS2, OPRM1, FLT4, THPO, CSK, MTOR, TEC, TXK, PTGIR, RAB9A, BLK, BMX, JAK3, BLM, ESRRG, PPIA, FKBP1A, FKBP1B, CACNA1D, FKBP4, GRIN1, BTK, PPP3CA, DDR1, PPID, KCNH2, STK4, MAP2K5, STK3, GRIN2B, FKBP5, ROCK1, PRPF4B, CAMK2D, CACNA1S, PTK6, CACNA1C, ERBB4, CA9, DDR2, AAK1, PDCD4, MAP3K19, SLC47A1, SRPK1, EBPL, NUAK2, CLK4, TDP1, DHCR7, SRPK3, CAMK2A, RIPK2, IMPDH2, SRC, BMPR1B, AURKA, EPHB6, MAPK13, PRPF4, NUAK1, PAK4, ALDH1A1, RAF1, ATP1A1, ATP1B1, CYP17A1, PRKCB, CHRM2, MET, ARAF, CYP2D6, THRB, PIM1, CYP19A1, CFTR, ATP1A3, ATP1B2, BRAF, CYP11B2, EPHA1, ERBB3, DRD5, CPT2, MAPK3, MARK3, MAPK1, EPHA2, EPHA3, EPHA8, EPHB2, CCND2, AKT1, AKT2, EPHX2, PTGER1, SCN1A, SCN4A, TIE1, MPL, GRIA1, GRIA2, GRIA3, FRK, PTGER2, SYK, MAPK8, MAPK9, GRIA4, MAPKAPK2, CDK8, FNTA, FNTB, CDK7, ATP1A2, PLK1, LIMK2, MAPK12, MAPK10, ATP1B3, FXYD2, EPHB3, EPHA5, EPHB4, EPHB1, EPHA4, SIK1, KCNJ2, MAP3K9, SCN7A, MYT1, TEK, ACVR1, TNK2, CYP24A1, PRKG2, TNK1, NAE1, DYRK1A, ATP1A4, SCN5A, TESK1, MAPK11, GRK1, SCN9A, PKN1, PKN2, MAP3K11, CYP51A1, TNNI3K, MARK2, PIM3, HIPK4, UBA3, MAPK15, GRK7, CPT1B, AURKB, CAMKK2, SCN2A, PKMYT1, RIOK2, TSSK1B, SIK2, SRMS, EML4, SCN3A, MAP3K20, PIM2, EIF2AK4, TBK1, SCN11A, SCN8A, PDE10A, AKT3, SIK3, HCN4, DYRK1B, MAP4K5, SCN10A, RBX1, IKZF1, CUL4A, DDB1, CRBN, IKZF3, KCNK3, RIOK3, XPO1, DCLK1, GRIN2D, TLR3, DAPK3, MAP3K7, CA12, PDE6D, GRIN3B, GUCY1B2, GMNN, STK16, PDE5A, STK10, KCNK2, S1PR2, ESRRB, S1PR4, DHFR, F2, PLG, REN, SERPINC1, C5, TNF, IFNG, IL1B, KLKB1, MMP1, HMGCR, CSF2, MAS1, SHBG, TUBB4A, NTRK1, TYMS, ITGB3, CYP1A2, IL6, TUBB, ADRB2, SERPINA6, NR3C2, ITGA2B, CD14, ADRB1, IL6R, MMP7, HMGB1, LTA4H, ADORA3, TUBA3C, CXCL8, AR, ESRRA, ACE, SLC5A1, ADRB3, INSRR, IL1R1, CSF2RA, VEGFA, ALOX15, SELP, TSHR, PDE6A, FER, IFNAR1, SRD5A1, PDE6G, NFKB1, CYP3A5, S1PR1, CNR1, GART, ACHE, MMP8, PTGS1, CA6, GLRA1, JAK1, CYP3A7, EDNRB, TBXAS1, HRH2, EDNRA, TACR1, DPP4, HTR1D, ADORA2A, ADORA2B, CCN2, TYK2, CD6, AVPR2, OXTR, GNRHR, SRD5A2, SLC5A2, ATIC, GRK4, CSF2RB, CD80, TTK, ADRA1A, ADRA1B, PDE6B, MAP2K2, AVPR1A, OPRD1, OPRK1, SLC19A1, CD86, AKR1C3, CA7, MMP13, AVPR1B, MTNR1A, IFNAR2, MTNR1B, CLK1, GSK3B, AGTR2, PDE6C, CYP2J2, CCR5, RPS6KA3, DAPK1, PRKAA2, KCNK10, TUBA1B, TUBA4A, TUBB4B, PIP4K2B, SRPK2, CDK16, CDK17, SLC6A3, DHODH, GUCY1B1, MAP2K1, TOP2B, AOX1, IL10RB, ITK, MERTK, GRIN2A, FAP, PRKAA1, TUBB3, CAMK2G, BMPR2, TUBB2A, PDE6H, PRKG1, PTK2B, MELK, GRIN2C, PDCD1, RPS6KA2, SF3B3, STK11, NTRK3, IL17A, NTRK2, HIF1A, TUBB8, LRRK2, TUBA3E, AC091230.1, NPSR1, TUBA1A, KCNK18, IFNLR1, MINK1, GRIN3A, MAP4K1, S1PR3, GPER1, HSD17B10, PIP5K1A, MAP3K3, SLC29A1, TUBA1C, RIOK1, TUBB6, TUBB2B, DCLK3, S1PR5, P2RY12, SLK, TUBB1, SRD5A3, CYP3A43, KCNK9, CYSLTR2, BMP2K, SLC5A4, STK26, DAPK2, TNIK, CYSLTR1, MAP3K2, PTGDR2, GLA, COMT, BRD3, F8, IL2RA, FGB, VWF, CD2, CD3E, ALOX5, MS4A1, ITGA4, PKM,*	PMID: 33837377

	IL2RB, GLUL, IFNGR1, NQO1, ALOX12, ITGAL, CXCR1, CXCR2, F2R, ITGB7, HSD3B2, PDE4A, IL12B, CD52, PPARG, IFNGR2, CCR2, RECQL, CXCR4, ADAM17, PPAT, PDE4B, PDE4C, AC008397.2, PDE4D, PTGDR, GPR17, KEAP1, PDE3A, IL11RA, TXNRD1, CA13, CSF3R, VKORC1, HRH4, NOD2, GPR35, IL23A, TNFSF13B, CA5B, HRH3, ACE2, TLR4, PSMD11, CDC7, PSMD14, STK25, TNFRSF10B, PSMA7, PDE8A, CTSV, NCOR1, ULK1, IDH1, TNFSF13, CHEK2, PLAT, ADA, IL1A, TTR, AFP, CXCL10, TFRC, APOB, TK1, CD74, APP, ALDH2, IL4, MPO, ITGB1, CEACAM5, NPM1, ITGAV, H1-0, HSP90AA1, HSP90AB1, ITGA5, VIM, GSTP1, CSF1, PARP1, CALM1, BCL2, SPP1, HSPA8, TOP1, ADH5, CCL2, EEF2, PLA2G2A, AKR1B1, FOLR1, CD19, PHKG2, MUC1, CD44, EPCAM, PRKCA, PRKACA, TNNI3, DDC, IL10, PRKACG, PRKACB, RPS6KB1, RRM1, PSMA2, PSMA3, PSMA4, PSMB9, PSMA5, PSMB5, BDKRB2, CPS1, RRM2, KIF5B, GUCY1A2, ABCC1, SOAT1, BSG, PSMC2, SNCA, GGCX, IL6ST, HTT, GLP1R, GPD2, PSMC4, BDKRB1, PSMD8, IDH2, PSMB3, PSEN1, GSK3A, PSMD7, KIF11, NR1H2, GFER, HDAC4, PSMA6, PSMC1, PSMC5, HBA2, HBA1, PRKDC, NFKB2, KMT2A, PTK2, PRKCZ, PRKCD, BCL2L1, MCL1, MFGE8, PAK2, PSMD2, MAP3K1, HDAC1, IL18, PSMD6, PDK1, RPS6KA1, SHH, MYLK, ADRM1, CCDC6, BCL2A1, TMEM97, RRM2B, DPP9, MAPKAPK5, ABHD12, PCSK9, TRPV1, HDAC7, MUC16, HDAC2, BCL2L2, BCL2L2-PABPN1, PSMD1, GPA33, TSG101, IL22, RTN4, RPS6KB2, STK17A, PSMD13, NCOR2, STK24	

Table S15: Functional enrichment analysis of 190 up-DEGs associated with severe COVID-19 based on the Reactome database

Description	Size	Ratio	P Value	FDR
Immune System	1997	2.56	1.65E-14	2.86E-11
Innate Immune System	1053	3.24	1.79E-12	1.55E-09
Neutrophil degranulation	479	4.07	3.39E-09	1.95E-06
Negative regulators of DDX58/IFIH1 signaling	34	16.71	1.49E-07	6.45E-05
Cellular responses to external stimuli	503	3.23	3.15E-06	1.09E-03
DDX58/IFIH1-mediated induction of interferon-alpha/beta	78	8.33	4.86E-06	1.40E-03
Interleukin-4 and Interleukin-13 signaling	108	6.77	6.92E-06	1.71E-03
Adaptive Immune System	756	2.58	1.45E-05	3.13E-03
Interferon alpha/beta signaling	69	8.24	2.09E-05	4.02E-03
Cellular responses to stress	426	3.05	6.63E-05	1.15E-02
Regulation of TLR by endogenous ligand	19	17.09	7.38E-05	1.16E-02
Cytokine Signaling in Immune system	688	2.48	9.46E-05	1.36E-02
Interferon gamma signaling	92	6.18	1.34E-04	1.79E-02
Interferon Signaling	197	4.12	1.56E-04	1.93E-02
Class I MHC mediated antigen processing & presentation	371	3.06	1.86E-04	2.14E-02
Antigen processing-Cross presentation	99	5.74	2.13E-04	2.30E-02
IRF3-mediated induction of type I IFN	13	18.74	4.77E-04	4.85E-02

Table S16: Disease-based enrichment analysis of 190 up-DEGs associated with severe COVID-19 among *CCR1*+ CD16+monocytes based on the GLAD4U database.

Disease terms	Size	Expect	Enrichment Ratio	P Value	FDR
Infection	643	5.19	5.40	2.90E-13	7.88E-10
Virus Diseases	580	4.68	5.56	1.17E-12	1.59E-09
Inflammation	565	4.56	5.48	4.42E-12	4.01E-09
Hepatitis	253	2.04	7.84	2.45E-10	1.67E-07
Immune System Diseases	806	6.50	3.84	7.35E-09	4.00E-06
Necrosis	371	2.99	5.35	5.79E-08	2.62E-05
Hyperoxia	33	0.27	22.54	2.32E-07	9.03E-05
HIV	862	6.95	3.31	4.60E-07	1.37E-04
Respiratory Tract Infections	281	2.27	5.73	4.85E-07	1.37E-04
virological response	282	2.28	5.71	5.05E-07	1.37E-04
Sexually Transmitted Diseases	496	4.00	4.25	5.76E-07	1.42E-04
Encephalitis, Viral	66	0.53	13.15	1.02E-06	2.31E-04
Bacterial Infections	260	2.10	5.72	1.39E-06	2.91E-04
Retroviridae Infections	494	3.99	4.01	2.62E-06	4.87E-04
HIV Infections	495	3.99	4.01	2.69E-06	4.87E-04
Lentivirus Infections	498	4.02	3.98	2.90E-06	4.93E-04
Hepatitis B	190	1.53	6.52	3.39E-06	5.42E-04
Mouth Diseases	246	1.98	5.54	5.25E-06	7.93E-04
Reperfusion Injury	86	0.69	10.09	6.15E-06	8.80E-04
Encephalitis	89	0.72	9.75	7.73E-06	1.05E-03
West Nile Fever	35	0.28	17.71	8.58E-06	1.11E-03
Immunologic Deficiency Syndromes	500	4.03	3.72	1.36E-05	1.66E-03
Rhinitis	135	1.09	7.35	1.40E-05	1.66E-03
Tumor Virus Infections	186	1.50	6.00	2.07E-05	2.35E-03
Psoriasis	243	1.96	5.10	2.90E-05	3.16E-03

Autoimmune Diseases	546	4.40	3.41	3.76E-05	3.93E-03
Arthritis, Reactive	78	0.63	9.54	4.00E-05	3.99E-03
Skin and Connective Tissue Diseases	617	4.98	3.21	4.11E-05	3.99E-03
Skin Diseases, Viral	85	0.69	8.75	6.50E-05	6.07E-03
Stress	643	5.19	3.08	6.70E-05	6.07E-03
Connective Tissue Diseases	392	3.16	3.79	8.40E-05	7.32E-03
Hepatitis, Chronic	174	1.40	5.70	8.61E-05	7.32E-03
Liver Neoplasms	396	3.19	3.76	9.25E-05	7.61E-03
Periodontitis	91	0.73	8.17	9.52E-05	7.61E-03
Respiratory Syncytial Virus Infections	229	1.85	4.87	1.04E-04	8.09E-03
Gram-Positive Bacterial Infections	181	1.46	5.48	1.13E-04	8.56E-03
Periodontal Diseases	96	0.77	7.75	1.28E-04	9.41E-03
Dermatitis, Atopic	140	1.13	6.20	1.43E-04	1.02E-02
Dermatomyositis	34	0.27	14.58	1.57E-04	1.09E-02
Hepatitis C	195	1.57	5.09	1.89E-04	1.27E-02
Lipidoses	67	0.54	9.25	2.07E-04	1.31E-02
Pneumonia	109	0.88	6.82	2.57E-04	1.45E-02
Adenocarcinoma	522	4.21	3.09	3.27E-04	1.81E-02
Chondrodysplasia Punctata	83	0.67	7.47	5.62E-04	2.88E-02
Lupus erythematosus	294	2.37	3.79	6.56E-04	3.08E-02
Leukemia	565	4.56	2.85	6.87E-04	3.11E-02
Pharyngeal Neoplasms	137	1.11	5.43	8.66E-04	3.62E-02
Frostbite	6	0.05	41.32	9.50E-04	3.64E-02
Actinomycetales Infections	145	1.17	5.13	1.16E-03	4.00E-02
Measles	62	0.50	8.00	1.58E-03	4.73E-02

Table S17: 190 up-DEGs associated with severe COVID-19 among *CCR1*+ CD16+monocytes matched in druggable gene categories based on the DGIdb resource

Druggable Gene Category	Matching Gene Count	Matching Gene(s)
Druggable genome	65	*AHR, APOBEC3A, ASGR1, C1QB, CD14, CD163, CD300E, CD36, CD53, CD63, CD84, CD99, CDKN1A, CTSA, CTSD, CTSL, CXCL8, CYP1B1, FKBP5, FOLR3, FPR1, FPR2, GLUL, HBEGF, HIF1A, HRH2, HSPA5, ICAM1, IFITM1, IGFBP7, IL4R, ISG15, LAIR1, LDHA, LGALS3BP, MGST1, NAMPT, PARP9, PGD, PIM1, PIM3, PLBD1, PLSCR1, PPIF, PSMA1, PXK, RNASE2, S100A12, S100A8, S100A9, SELL, SERPING1, SGK1, SIGLEC1, SLC12A7, SLC25A37, SLC2A3, SMPDL3A, TCN2, TNFAIP2, TNFSF10, TXN, TXNRD2, UPP1, VCAN*
Enzyme	35	*AHR, APOBEC3A, ATG12, BLVRB, CTSA, CTSD, CTSL, CYP1B1, DYNLL1, GLRX, GLUL, GM2A, HIF1A, HSPA5, IFIH1, ISG15, KRTCAP2, NCF1, PARP9, PGD, PIM1, PIM3, PXK, S100A8, S100A9, SGK1, SMPDL3A, SUMO3, SUPT5H, TNFAIP3, UBE2B, UBE2K, UBE2L3, UBE2V2, UPP1*
Kinase	21	*CCNL1, CD163, CD300E, CD63, CDKN1A, GADD45GIP1, HRH2, IFIH1, IRF3, MPLKIP, PIM1, PIM3, PXK, RGCC, S100A12, S100A8, S100A9, SGK1, SOCS3, STING1, TNFAIP3*
Clinically actionable	18	*BCL3, CAMTA1, CD36, CDKN1A, CYP1B1, ETV6, HIF1A, H2BC12, IRF2, MAML2, NOP10, PER1, PIM1, PRCC, SGK1, SOCS3, STING1, TNFAIP3*

Transcription factor	14	*AHR, ATF3, CAMTA1, CEBPB, CEBPD, ETV6, HIF1A, ID1, IFI16, IRF2, IRF3, PER1, SCAND1, SGK1*
Cell surface	8	*C1QB, CD36, CD53, CD63, HBEGF, HSPA5, ICAM1, VAMP5*
Transporter	8	*CD36, EMB, HIF1A, HSPA5, SGK1, SLC12A7, SLC25A37, SLC2A3*
Transcription factor binding	7	*AHR, BCL3, HIF1A, ID1, IFI16, PIM1, STING1*
Drug resistance	6	*CDKN1A, ICAM1, LDHA, MGST1, SGK1, UBE2B*
External side of plasma membrane	6	*CD14, CD163, CD36, FLOT1, ICAM1, SLC12A7*
Protease	5	*CTSA, CTSD, CTSL, PSMA1, TNFAIP3*
Protease inhibitor	4	*APLP2, HSPA5, NAIP, SERPING1*
Serine threonine kinase	4	*PIM1, PIM3, PXK, SGK1*
G protein coupled receptor	3	*FPR1, FPR2, HRH2*
Nuclear hormone receptor	3	*AHR, GADD45GIP1, PER1*

Table S18: Highly-expressed inflammatory and cytokine genes among *ABO*+ megakaryocytes

Gene name	T score	Fold change	P value	FDR
ADORA2B	2.80	2.27	5.29E-03	2.227E-02
ADRM1	3.02	1.66	2.72E-03	1.301E-02
AHR	2.52	2.25	1.22E-02	4.349E-02
GNA15	3.49	1.82	5.35E-04	3.461E-03
IRAK2	3.20	2.85	1.48E-03	7.919E-03
KCNA3	3.29	1.89	1.10E-03	6.179E-03
PDGFA	4.26	1.71	2.53E-05	2.666E-04
PTGIR	4.05	1.56	6.18E-05	5.671E-04
SPHK1	3.71	1.57	2.36E-04	1.745E-03
BMP6	2.90	2.44	3.96E-03	1.766E-02
TNFSF4	4.81	2.67	2.18E-06	3.138E-05

Table S19: Pathway enrichment analysis of 424 highly-expressed genes among *ABO+* megakaryocytes

**Pathway name**	**Gene size**	**Enrichment ratio**	**P Value**	**FDR**
Systemic lupus erythematosus	133	4.79	4.55E-07	1.48E-04
Alcoholism	180	4.01	9.82E-07	1.60E-04
Platelet activation	123	3.80	1.42E-04	1.55E-02
SNARE interactions in vesicular transport	34	6.24	1.10E-03	8.94E-02
Endocytosis	244	2.43	1.80E-03	1.17E-01
Viral carcinogenesis	201	2.53	2.74E-03	1.28E-01
VEGF signaling pathway	59	4.32	2.52E-03	1.28E-01
Necroptosis	162	2.62	4.83E-03	1.61E-01
Mitophagy	65	3.92	4.11E-03	1.61E-01
Chemokine signaling pathway	189	2.47	4.95E-03	1.61E-01
Transcriptional misregulation in cancer	186	2.28	1.23E-02	3.65E-01
Axon guidance	175	2.18	2.23E-02	6.05E-01
Bacterial invasion of epithelial cells	74	2.87	2.99E-02	6.51E-01
Regulation of actin cytoskeleton	213	1.99	2.88E-02	6.51E-01
Adherens junction	72	2.95	2.70E-02	6.51E-01
Human cytomegalovirus infection	225	1.89	3.97E-02	8.08E-01
Proteoglycans in cancer	201	1.90	4.77E-02	8.64E-01
Tight junction	170	2.00	4.73E-02	8.64E-01

Table S20: Disease-term enrichment analysis of 35 up-DEGs associated with severe COVID-19 among *ABO*+ megakaryocytes based on the GLAD4U database

Term ID	Disease-terms	Size	Expect	Enrichment Ratio	P Value
PA443490	Bernard-Soulier Syndrome	9	0.01	140.31	8.69E-05
PA445644	Shock	367	0.58	8.60	2.59E-04
PA443842	Death	417	0.66	7.57	4.65E-04
PA445846	Thrombocytopenia	96	0.15	19.73	4.67E-04
PA443382	Anoxia	251	0.40	10.06	6.36E-04
PA445457	Pterygium	25	0.04	50.51	7.13E-04
PA165108957	Venous ulcer of leg	26	0.04	48.57	7.71E-04
PA166048906	Blood Platelet Disorders	118	0.19	16.05	8.51E-04
PA444668	Keratitis	31	0.05	40.74	1.10E-03
PA166129556	disease activity score 28 joint in rheumatoid arthritis	31	0.05	40.74	1.10E-03
PA443429	Arteritis	34	0.05	37.14	1.32E-03
PA443882	Dermatomyositis	34	0.05	37.14	1.32E-03
PA445850	Thrombosis	141	0.22	13.43	1.42E-03
PA166123766	platelet aggregation	153	0.24	12.38	1.80E-03
PA446220	Abdominal Pain	43	0.07	29.37	2.11E-03
PA445793	Synovitis	47	0.07	26.87	2.51E-03
PA443433	Arthritis, Juvenile Rheumatoid	48	0.08	26.31	2.62E-03
PA445051	Necrosis	371	0.59	6.81	2.68E-03
PA446477	Polymyositis	49	0.08	25.77	2.73E-03
PA166048887	Irritable Bowel Syndrome	50	0.08	25.26	2.84E-03
PA445752	Stress	643	1.02	4.91	3.17E-03
PA445593	Sarcoidosis	56	0.09	22.55	3.55E-03
PA444034	Endocarditis	67	0.11	18.85	5.04E-03

PA443829	Cystic Fibrosis	223	0.35	8.49	5.20E-03
PA445044	Nasal Polyps	75	0.12	16.84	6.27E-03
PA444035	Endocarditis, Bacterial	76	0.12	16.62	6.44E-03
PA445512	Arthritis, Reactive	78	0.12	16.19	6.77E-03
PA446020	Varicose Veins	79	0.13	15.99	6.94E-03
PA165108622	Drug interaction with drug	494	0.78	5.11	7.38E-03
PA165374639	suicide	83	0.13	15.22	7.63E-03
PA446303	Tooth Loss	84	0.13	15.03	7.81E-03
PA444942	Methemoglobinemia	5	0.01	126.28	7.89E-03
PA447296	nondiabetic proteinuric nephropathy	5	0.01	126.28	7.89E-03
PA443474	Bacterial Infections	260	0.41	7.29	7.93E-03
PA445672	Sinusitis	89	0.14	14.19	8.73E-03
PA443690	Charcot-Marie-Tooth Disease	95	0.15	13.29	9.90E-03
PA445382	Polyps	95	0.15	13.29	9.90E-03
PA445296	Periodontal Diseases	96	0.15	13.15	1.01E-02
PA447230	HIV	862	1.37	3.66	1.08E-02
PA165108641	Acatalasia	7	0.01	90.20	1.10E-02
PA165108334	Ulnar neuropathy	8	0.01	78.93	1.26E-02
PA446833	Pouchitis	8	0.01	78.93	1.26E-02
PA445355	Pneumonia	109	0.17	11.59	1.29E-02
PA446540	Cytomegalovirus Retinitis	9	0.01	70.16	1.42E-02
PA447149	Chills	9	0.01	70.16	1.42E-02
PA444231	Fructose Intolerance	10	0.02	63.14	1.57E-02
PA443820	Cryptococcosis	11	0.02	57.40	1.73E-02

Table S21: 35 up-DEGs significantly associated with severe COVID-19 among *ABO*+ megakaryocytes matched in druggable gene categories

Druggable Gene Category	Matching Gene Count	Matching Gene(s)
Druggable genome	11	*ACP1, CFLAR, CYB5R3, FKBP8, HSPB1, MPIG6B, P2RX1, S100A8, S100A9, SMOX, TUBB1*
Enzyme	9	*ACP1, ATP6V1E1, CYB5R3, ENO1, HK1, S100A8, S100A9, SMOX, SUPT4H1*
Kinase	5	*HCST, HK1, S100A8, S100A9, SKP1*
Transporter	4	*ATF4, ATP6V1E1, SEC14L1, SLC44A2*
Cell surface	2	*ENO1, HCST*
Clinically actionable	1	*MYH9*
Drug resistance	1	*CFLAR*
External side of plasma membrane	1	*P2RX1*
Ion channel	1	*P2RX1*
Protease	1	*CFLAR*
Protein phosphatase	1	*ACP1*
Transcription factor	1	*ATF4*

Table S22: Pathway enrichment analysis of 158 highly-expressed genes among *CXCR6*+ memory CD8+T cells

Pathway name	Size	Enrichment Ratio	P Value	FDR
Cytokine-cytokine receptor interaction	294	5.65	1.24E-08	4.04E-06
Inflammatory bowel disease (IBD)	65	9.58	3.43E-05	5.58E-03
NF-kappa B signaling pathway	95	5.46	2.14E-03	2.32E-01
Toxoplasmosis	113	4.59	4.54E-03	2.85E-01
Necroptosis	162	3.84	4.55E-03	2.85E-01
Allograft rejection	38	8.19	5.69E-03	2.85E-01
Pyruvate metabolism	39	7.98	6.12E-03	2.85E-01
ABC transporters	44	7.07	8.57E-03	3.49E-01
Apoptosis	136	3.81	9.81E-03	3.55E-01
Intestinal immune network for IgA production	49	6.35	1.15E-02	3.76E-01
Autoimmune thyroid disease	53	5.87	1.43E-02	4.23E-01
Chagas disease (American trypanosomiasis)	102	4.07	1.66E-02	4.51E-01
Human cytomegalovirus infection	225	2.77	2.09E-02	5.25E-01
Kaposi sarcoma-associated herpesvirus infection	186	2.79	3.32E-02	6.83E-01
Citrate cycle (TCA cycle)	30	6.92	3.35E-02	6.83E-01
Chemokine signaling pathway	189	2.74	3.52E-02	6.83E-01
Circadian rhythm	31	6.69	3.56E-02	6.83E-01
Measles	132	3.14	3.81E-02	6.85E-01
Apoptosis	33	6.29	3.99E-02	6.85E-01
Phenylalanine, tyrosine and tryptophan biosynthesis	5	20.75	4.73E-02	7.71E-01

Table S23: GO-terms enrichment analysis of 108 up-DEGs associated with COVID-19 among *CXCR6*$^{+}$ memory CD8+T cells

GO-terms	Size	Enrichment ratio	P Value	FDR
Immune response-activating cell surface receptor signaling pathway	300	6.79	6.30E-07	2.83E-03
Positive regulation of immune system process	979	3.59	9.22E-07	2.83E-03
Immune response-activating signal transduction	454	5.30	9.34E-07	2.83E-03
Immune response-regulating cell surface receptor signaling pathway	330	6.17	1.60E-06	3.54E-03
Immune response-regulating signaling pathway	485	4.96	1.95E-06	3.54E-03
Post-translational protein modification	360	5.66	3.71E-06	4.83E-03
Regulation of immune system process	1400	2.91	3.72E-06	4.83E-03
Positive regulation of immune response	706	3.93	5.21E-06	5.63E-03
Activation of immune response	534	4.51	5.58E-06	5.63E-03
Leukocyte differentiation	496	4.48	1.41E-05	1.28E-02
Hemopoiesis	791	3.51	2.01E-05	1.66E-02
Hematopoietic or lymphoid organ development	833	3.33	3.67E-05	2.78E-02
Regulation of hemopoiesis	389	4.76	4.64E-05	3.25E-02
Immune response-regulating cell surface receptor signaling pathway involved in phagocytosis	76	12.18	5.61E-05	3.28E-02
Fc-gamma receptor signaling pathway involved in phagocytosis	76	12.18	5.61E-05	3.28E-02
Immune response	1919	2.32	5.76E-05	3.28E-02
Antigen receptor-mediated signaling pathway	184	7.04	6.16E-05	3.29E-02
Fc-gamma receptor signaling pathway	79	11.72	6.75E-05	3.32E-02
Immune system development	881	3.15	6.94E-05	3.32E-02
Fc receptor mediated stimulatory signaling pathway	81	11.43	7.61E-05	3.46E-02
Regulation of leukocyte differentiation	263	5.63	8.77E-05	3.80E-02
Regulation of immune response	909	3.06	9.86E-05	4.07E-02
