# Supplementary material for: Integrating single-cell sequencing data with GWAS summary statistics reveals CD16+monocytes and memory CD8+T cells involved in severe COVID-19
Source: Genome Med. 2022 Feb 17;14:16. doi: 10.1186/s13073-022-01021-1 (PMC8851814; doi:10.1186/s13073-022-01021-1)
Supplement: Supplementary file 3 — Additional file 3: Fig. S1. UMP projections of cells in PBMCs from normal controls, mild, moderate, and severe COVID-19 patients by using the Seurat R package (dataset #1). Fig. S2. Heatmap showing levels of well-known marker genes specific for each cell type in PBMCs. Fig. S3. Single-cell transcriptomes of PBMCs from normal controls, mild, moderate, and severe COVID-19 patients. Fig. S4. Hierarchical clustering using the PCC of a normalized transcriptome between controls and patients in cell type resolution. Fig. S5. Boxplots showing percentages of each cell type for PBMCs in donors from healthy control and COVID-19 patients. Fig. S6. scCODA determines the compositional differences of each cell type in PBMCs among donors from healthy control and COVID-19 patients. Fig. S7. Regional association plots for severe COVID-19-associated genetic loci based on meta-GWAS summary data. Fig. S8. Regional association plots for severe COVID-19-associated genetic loci based on meta-GWAS summary data. Fig. S9. Circus plot showing the results of MAGMA-based gene-level association analysis. Fig. S10. The 19 biological pathways enriched from the MAGMA-based pathway enrichment analysis. Fig. S11. High consistence results between MAGMA and S-MultiXcan analysis. Fig. S12. In silico permutation analysis of 100,000 times of random selections. Fig. S13. Multiple independent approaches identify genetics-relevant risk genes associated with severe COVID-19. Fig. S14. Plot of gene-drug interaction analysis for 34 risk genes. Fig. S15. The 10 biological pathways significantly enriched by 34 risk genes based on the KEGG database. Fig. S16. Barplots showing the results of RolyPoly among COVID-19 patients stratified by patient’s age. Fig. S17. Barplots showing the results of RolyPoly among COVID-19 patients stratified by patient’s sex. Fig. S18. Barplots showing the results of RolyPoly among COVID-19 patients stratified by patient’s BMI. Fig. S19. Barplots showing the results of RolyPoly COVID-19 [file 13073_2022_1021_MOESM3_ESM.pdf]

***Supplementary figures***

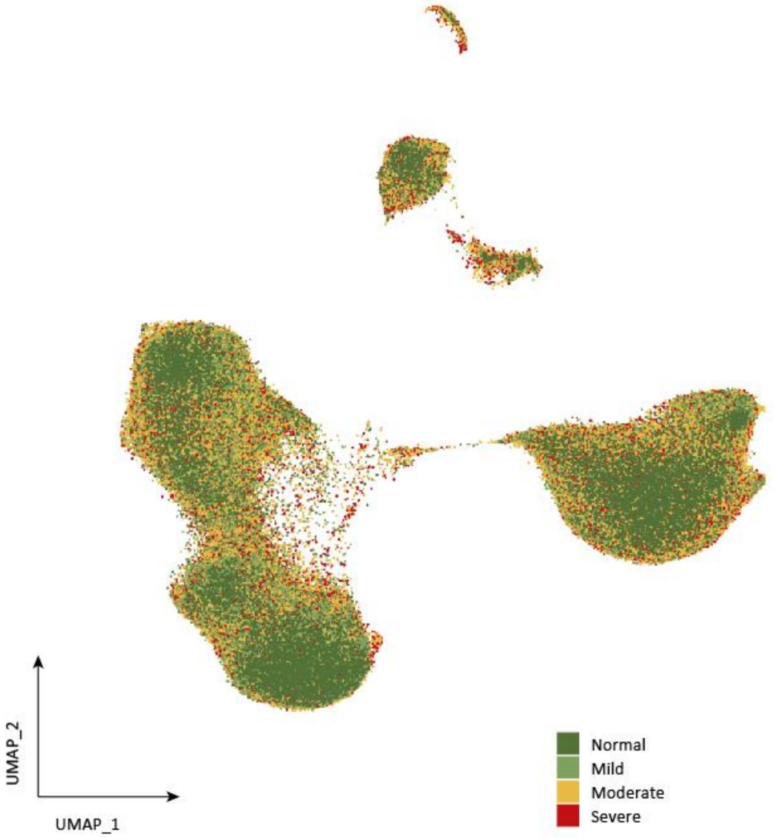


**Fig. S1. UMP projections of cells in PBMCs from normal controls, mild, moderate, and severe COVID-19 patients by using the Seurat R package (dataset #1).** Dark green represents normal controls, light green represents mild patients, orange represents moderate patients, and red represents severe patients.

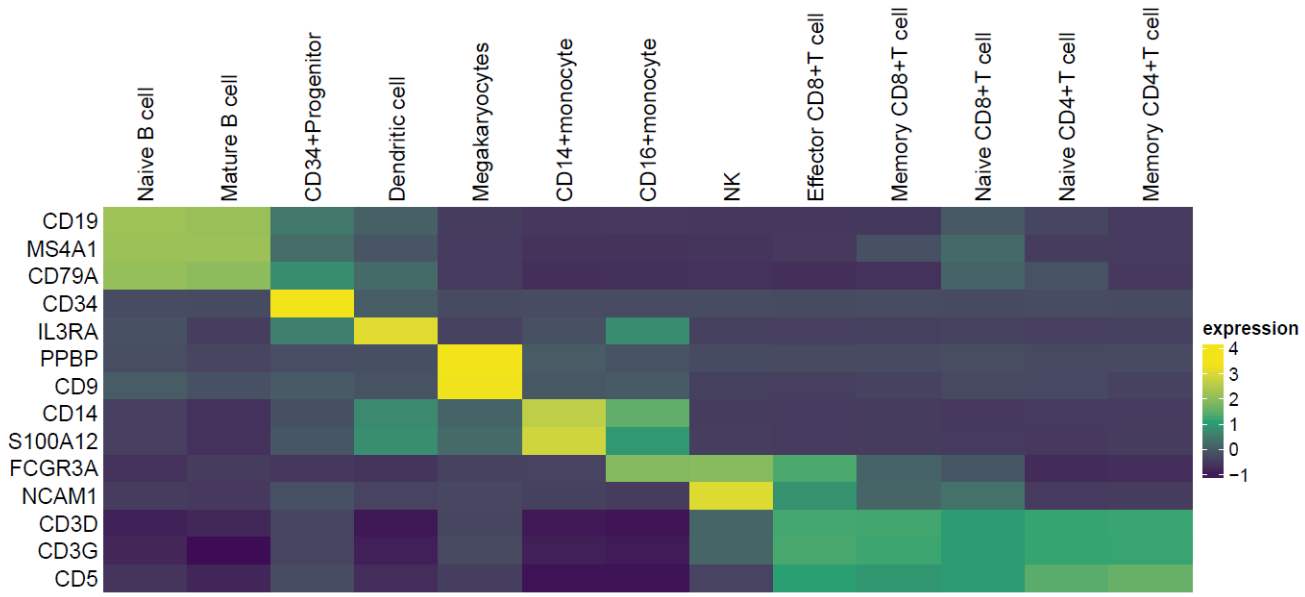


**Fig. S2. Heatmap showing levels of well-known marker genes specific for each cell type in PBMCs.** Color legend represents the expression level of each marker gene.

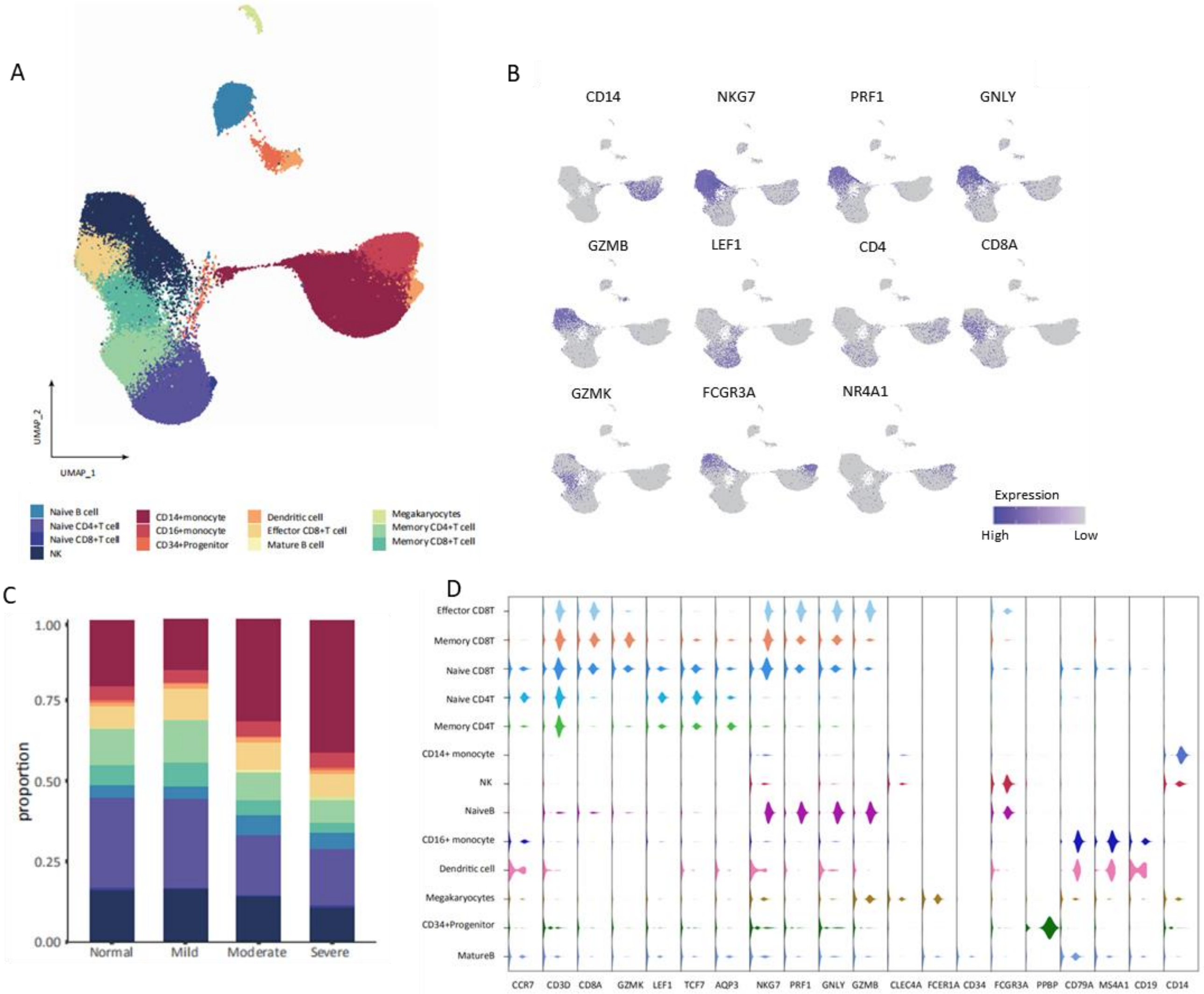


**Fig. S3. Single-cell transcriptomes of PBMCs from normal controls, mild, moderate, and severe COVID-19 patients.** A) UMAP projections of 514,400 cells from normal controls, mild, moderate, and severe patients colored by annotated cell types. There was 13 clusters with 13 distinct cell types annotated by using well-known markers. B) Normalized expression of marker genes on a UMAP plot. For example, the well-known markers of *CD14*, *NKG7*, *PRF1*, *GNLY*, *GZMB*, *LEF1*, *CD4*, *CD8A*, *GZMK*, *FCGR3A*, and *NR4A1*. C) Proportion of cell types among normal controls, mild, moderate, and severe patients. D) Violin plots of selected markers (lower row) for 13 cell subpopulations. The left column shows the cell types annotated by combinations of markers.

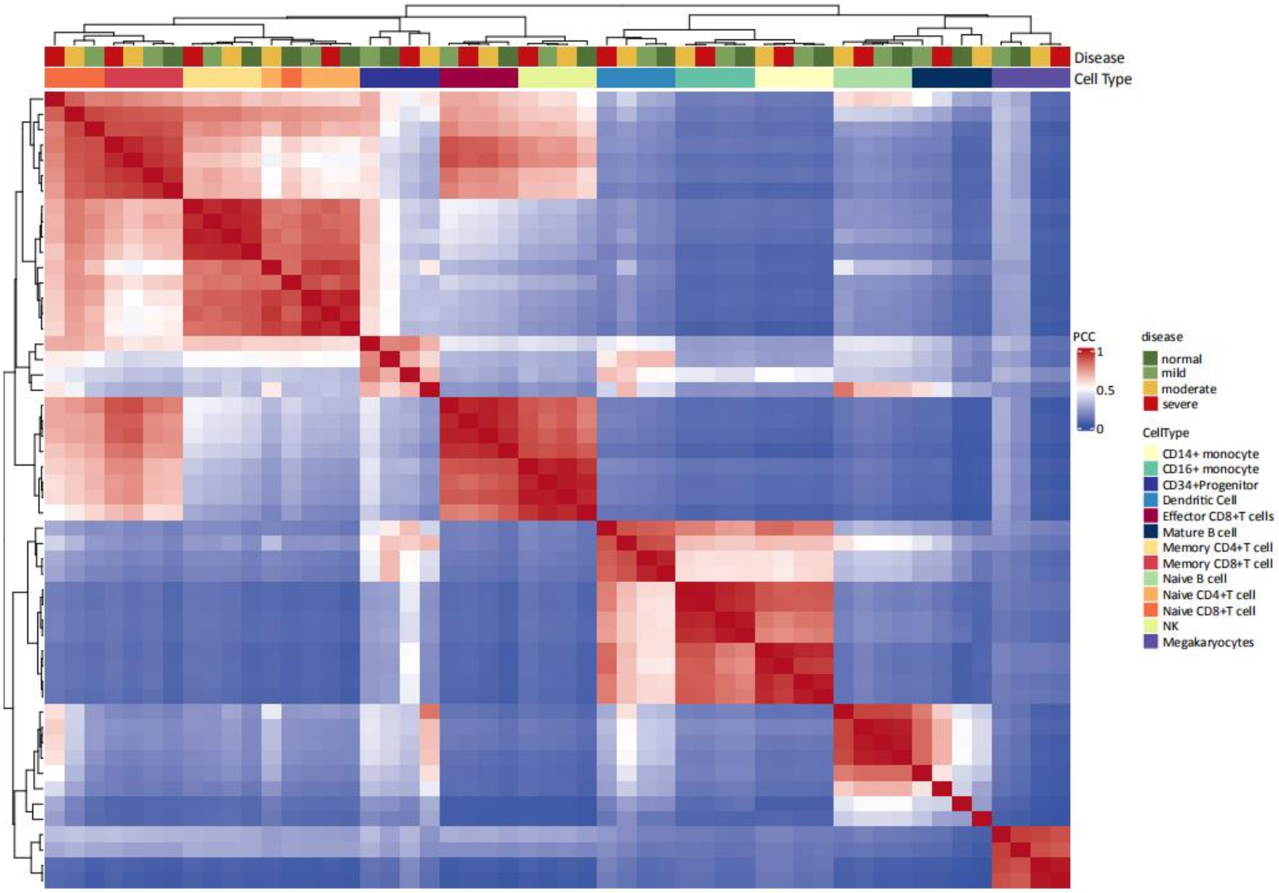


**Fig. S4. Hierarchical clustering using the PCC of a normalized transcriptome between diseases (normal and patients with different COVID-19 severities) in cell type resolution (n = 13).** The color intensity of the heatmap represents the PPC values. The color bars above the heatmap shows the disease group and cell type. This analysis was based on the largest scRNA-seq dataset of dataset #1 (accession number: E-MTAB-9357).

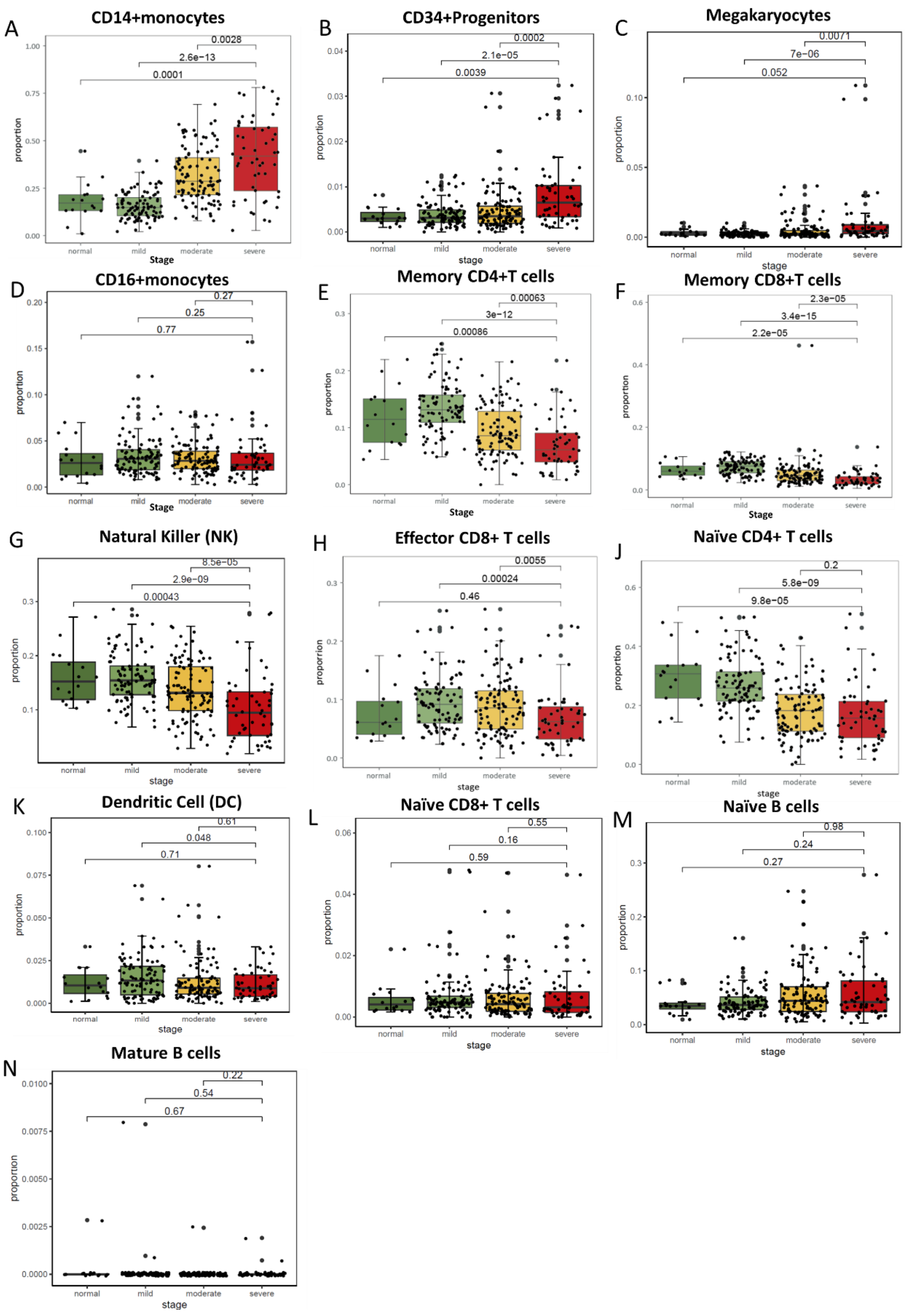


**Fig. S5. Boxplots showing percentages of each cell type in PBMCs among donors from healthy control and COVID-19 patients with different severities based on the scRNA-seq dataset.** The statistical analysis for calculating difference among different groups was based on the Wilcoxon sum-rank test.

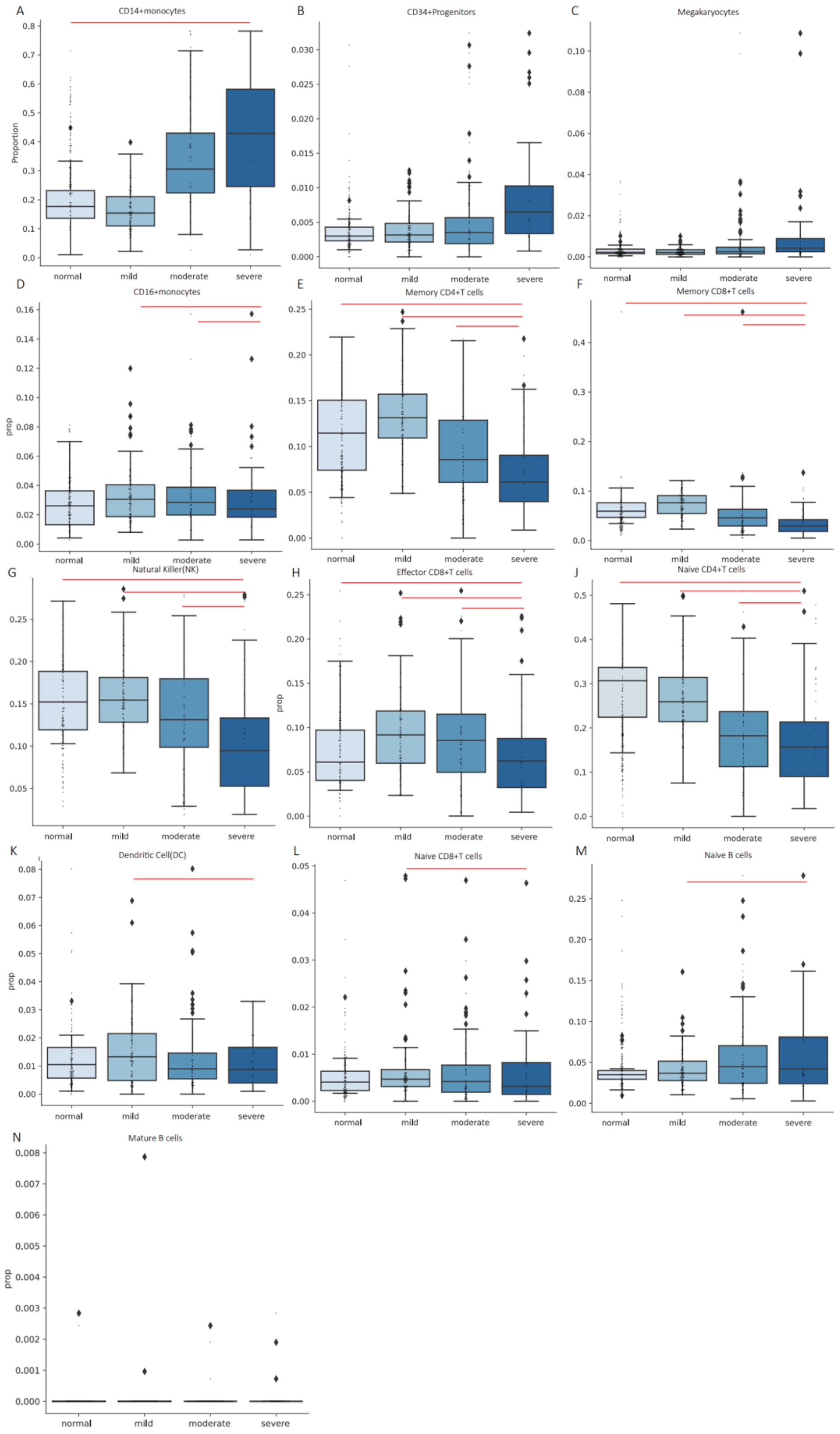


**Fig. S6. scCODA determines the compositional differences of each cell type in PBMCs among donors from healthy control and COVID-19 patients with different severities based on the scRNA-seq dataset.** Credible and significant results are depicted as red colored bars. These results are generated from an independent technique (i.e., scCODA) to validate the findings using the Wilcoxon rank-sum test in Additional file 3: Fig. S5.

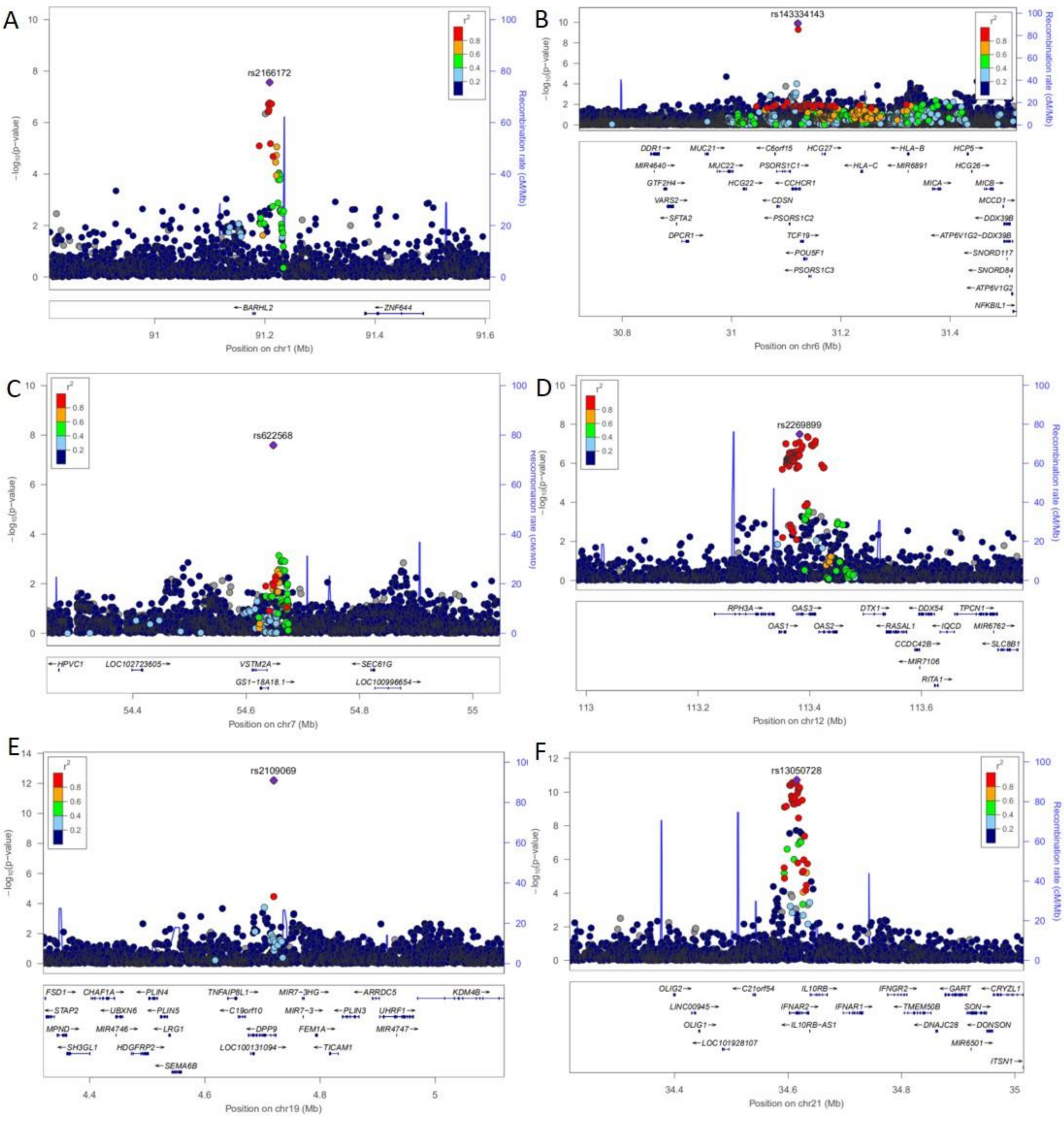


**Fig. S7. Regional association plots for severe COVID-19-associated genetic loci based on meta-GWAS summary data.** A-F) 1p22.2, 6p21.33, 7p11.2, 12q24.13, 19p13.3 and 21q22.11. The purple diamond marks the most strongly associated SNP in each locus with severe COVID-19. The color illustrates LD information with the given SNP, as shown in the color legend. The detailed information is shown in Additional file 2: Table S3.

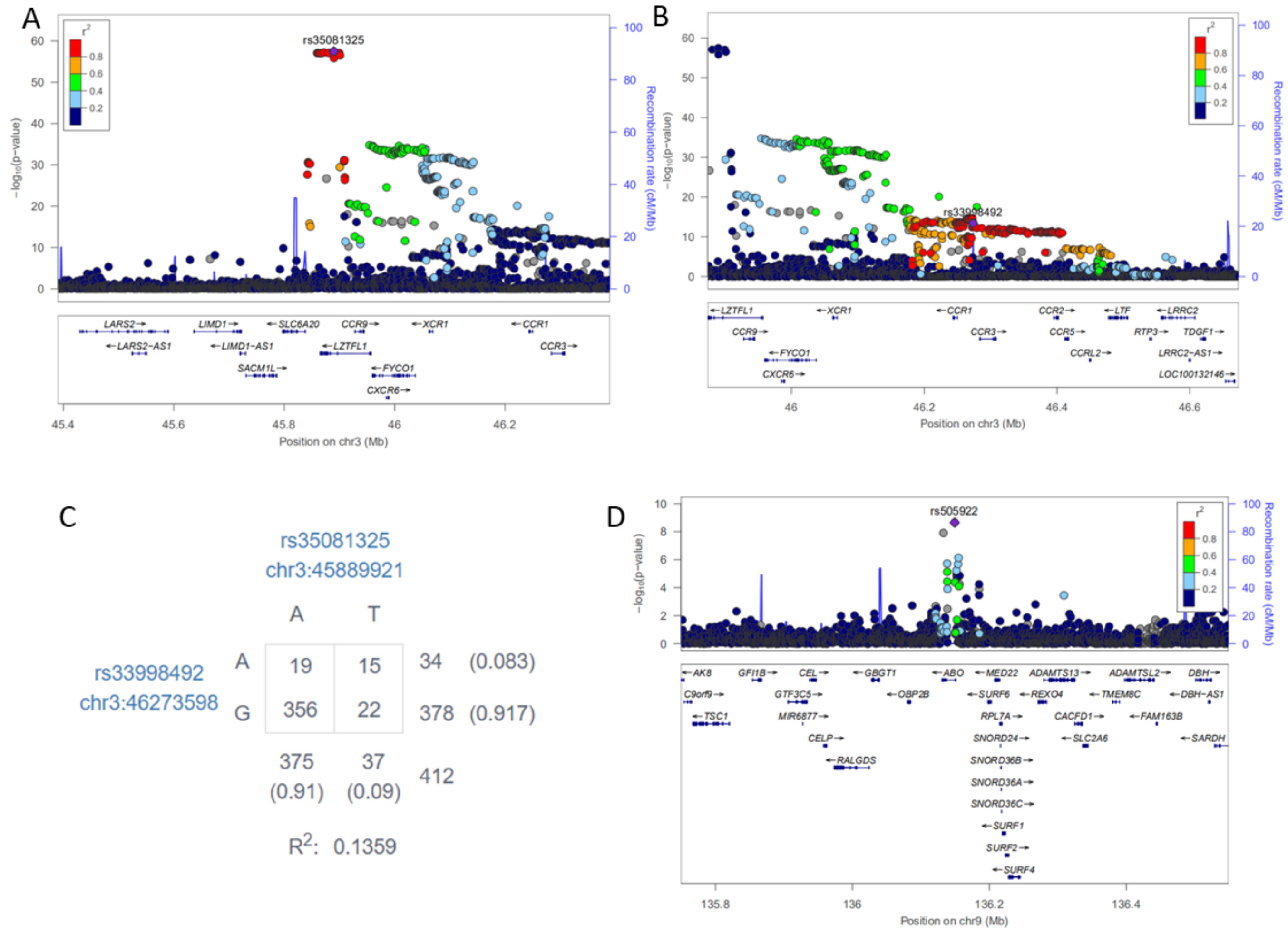


**Fig. S8. Regional association plots for severe COVID-19-associated genetic loci based on meta-GWAS summary data.** A-B) Two independent genetic association signals in the 3p21.31 loci (rs35081325, P = $3.32\times10^{-58}$, and rs33998492, P = $3.59\times10^{-14}$, respectively). The purple diamond marks the most strongly associated SNP in each locus with severe COVID-19. The color illustrates LD information with the given SNP, as shown in the color legend. C) The results of calculating the LD information between rs35081325 and rs33998492 using the LDpair Tool based on the European population (EUR+TSI). D) Regional association plot for the 9q34.2 locus associated with severe COVID-19.

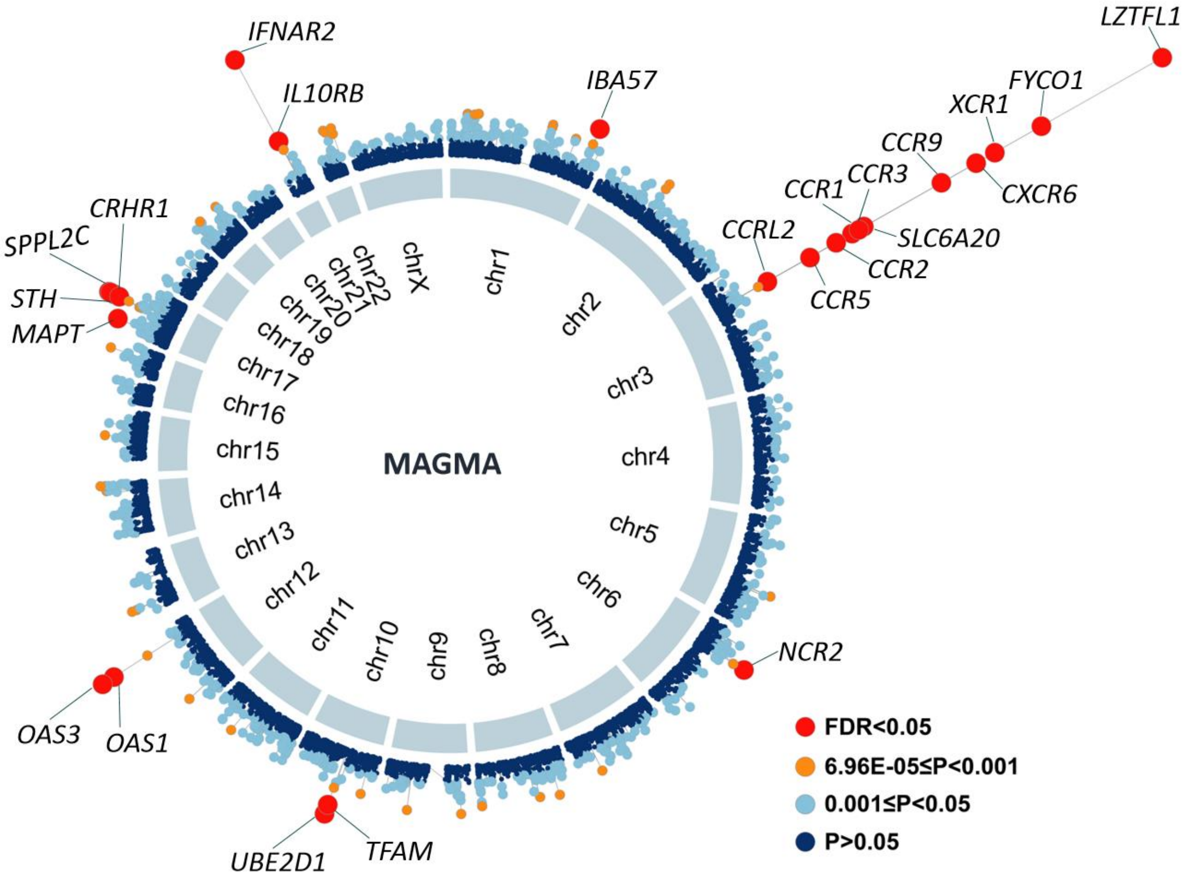


**Fig. S9. Circus plot showing the results of MAGMA-based gene-level association analysis.** A for lung tissue, and B for blood. The inner ring demonstrates the 22 autosomal chromosomes (Chr1-22) and X chromosome. In the outer ring, a circular symbol stands for a specific gene, and color marks the significant level of the gene. Red color marks genes significantly associated with severe COVID-19 with FDR < 0.05, orange color indicates genes suggestively associated with severe COVID-19 with $6.96 \times 10^{-5} \leq P < 0.001$, light blue color marks suggestive genes with $0.001 \leq P < 0.05$, and dark blue indicates genes have non-significant associations ($P > 0.05$).

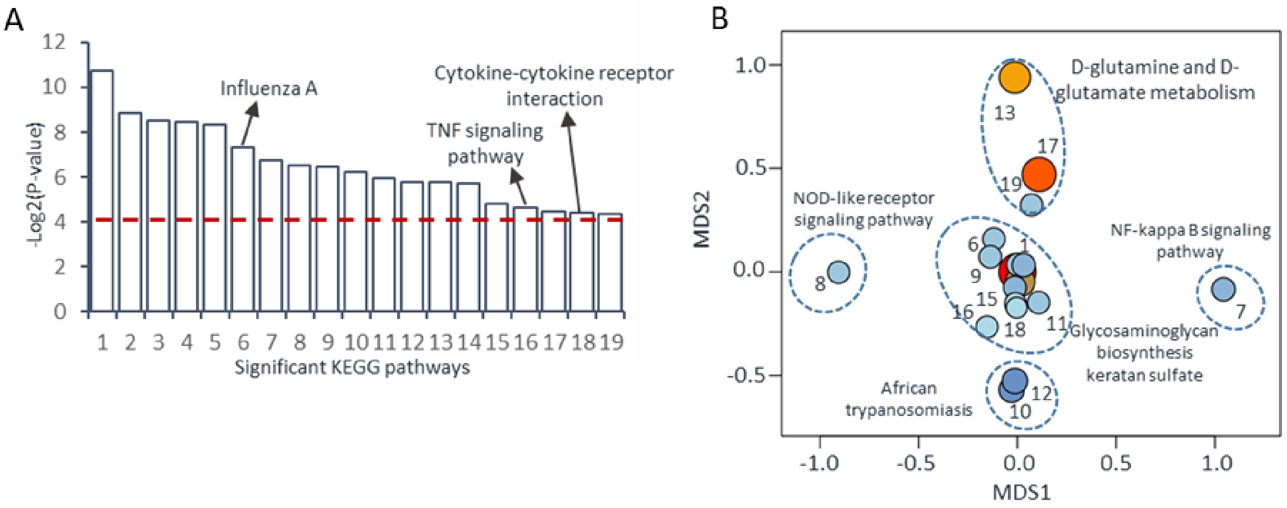


**Fig. S10. The 19 biological pathways enriched from the MAGMA-based pathway enrichment analysis.** A) Barplot showing the 19 biological pathways based on the KEGG pathway. B) Multidimensional scaling plot for clustering the 19 biological pathways based on their Jaccard distance (see the Methods). Color represents gene number in each pathway, and circular ring size indicates the Z score of enrichment for each pathway. The detailed information is shown in Additional file 2: Table S5.

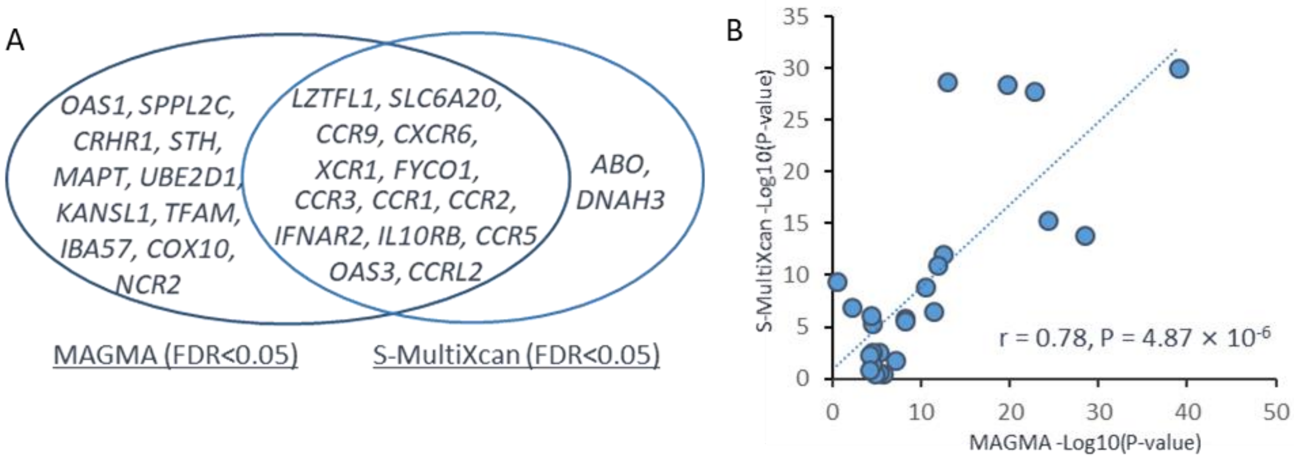


**Fig. S11. High consistence results between MAGMA and S-MultiXcan analysis.** A) Venn diagram exhibiting the overlapped significant genes between MAGMA and S-MultiXcan analysis. B) Correlation of significant risk genes identified frim MAGMA and S-MultiXcan analysis.

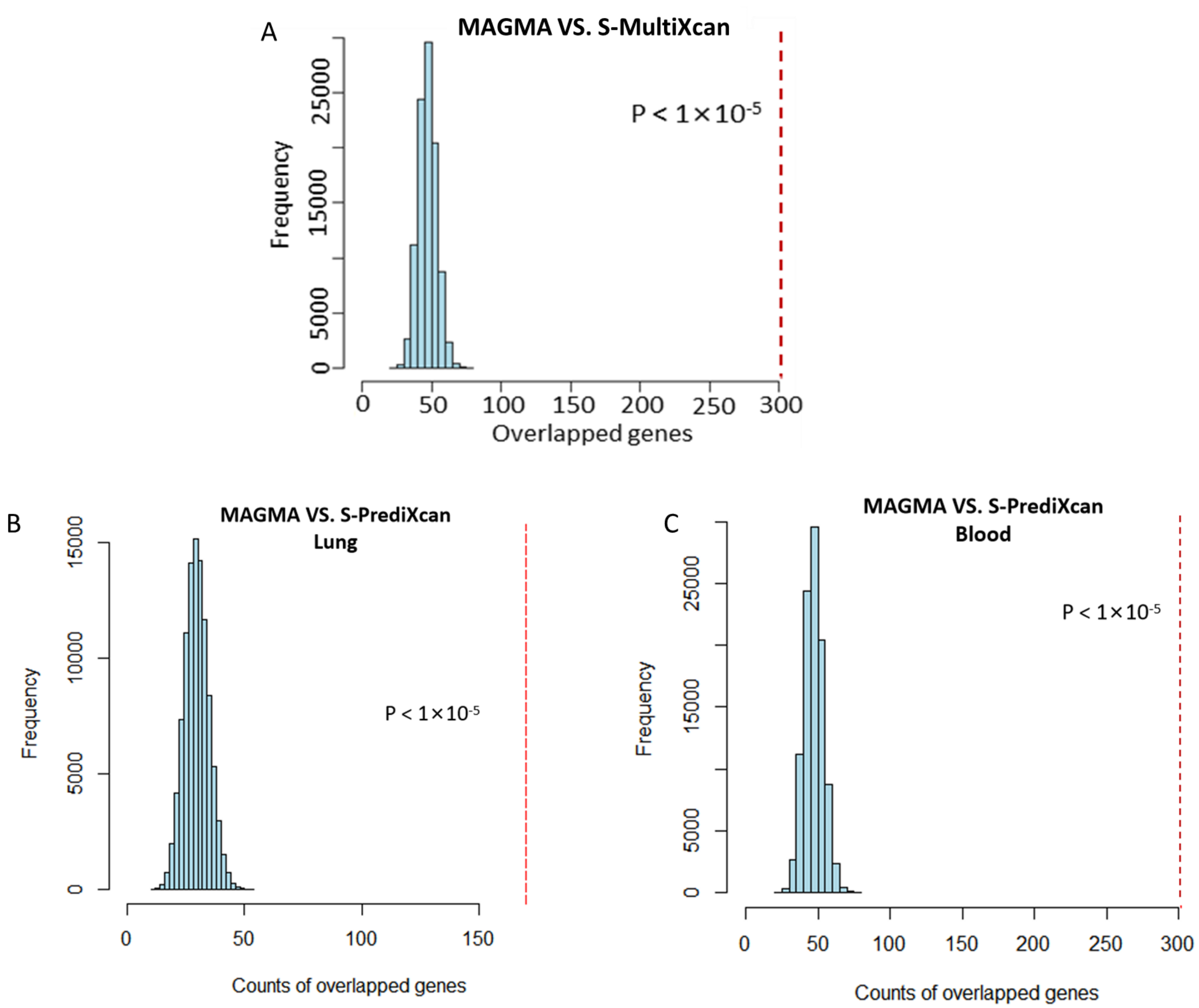


**Fig. S12. *In silico* permutation analysis of 100,000 times of random selections.** A) The comparison of the top-ranked genes from MAGMA gene-based analysis ($P < 0.05$) with that from S-MultiXcan-based analysis ($P < 0.05$). B) The comparison of the top-ranked genes from MAGMA gene-based analysis ($P < 0.05$) with that from S-PrediXcan-based analysis based on lung tissue ($P < 0.05$). C) The comparison of the top-ranked genes from MAGMA gene-based analysis ($P < 0.05$) with that from S-PrediXcan-based analysis based on blood tissue ($P < 0.05$).

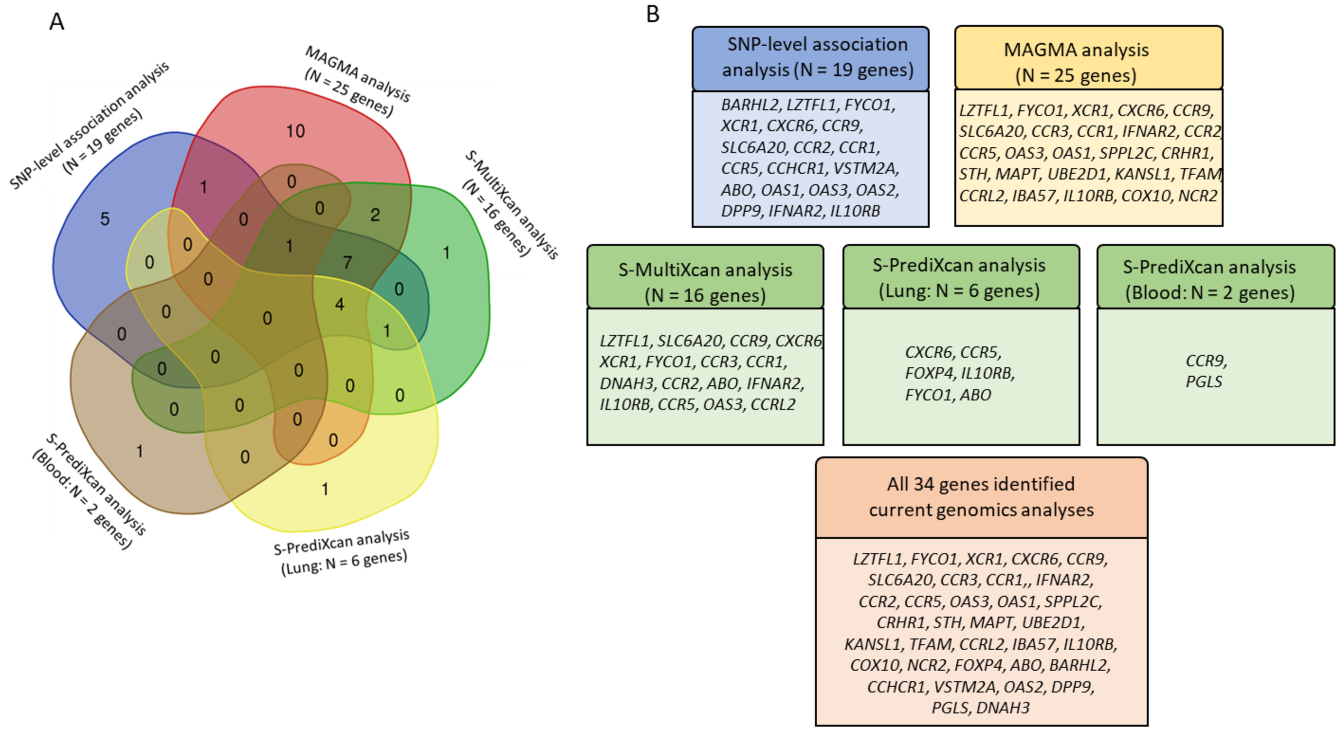


**Fig. S13. Multiple independent approaches identify genetics-relevant risk genes associated with severe COVID-19.** A) Venn diagram showing the overlapped genes among SNP-level association analysis, MAGMA gene-based analysis, S-MultiXcan analysis, and S-PrediXcan analysis based on lung and blood. B) Summary of total 34 genetically risk genes associated with severe COVID-19.

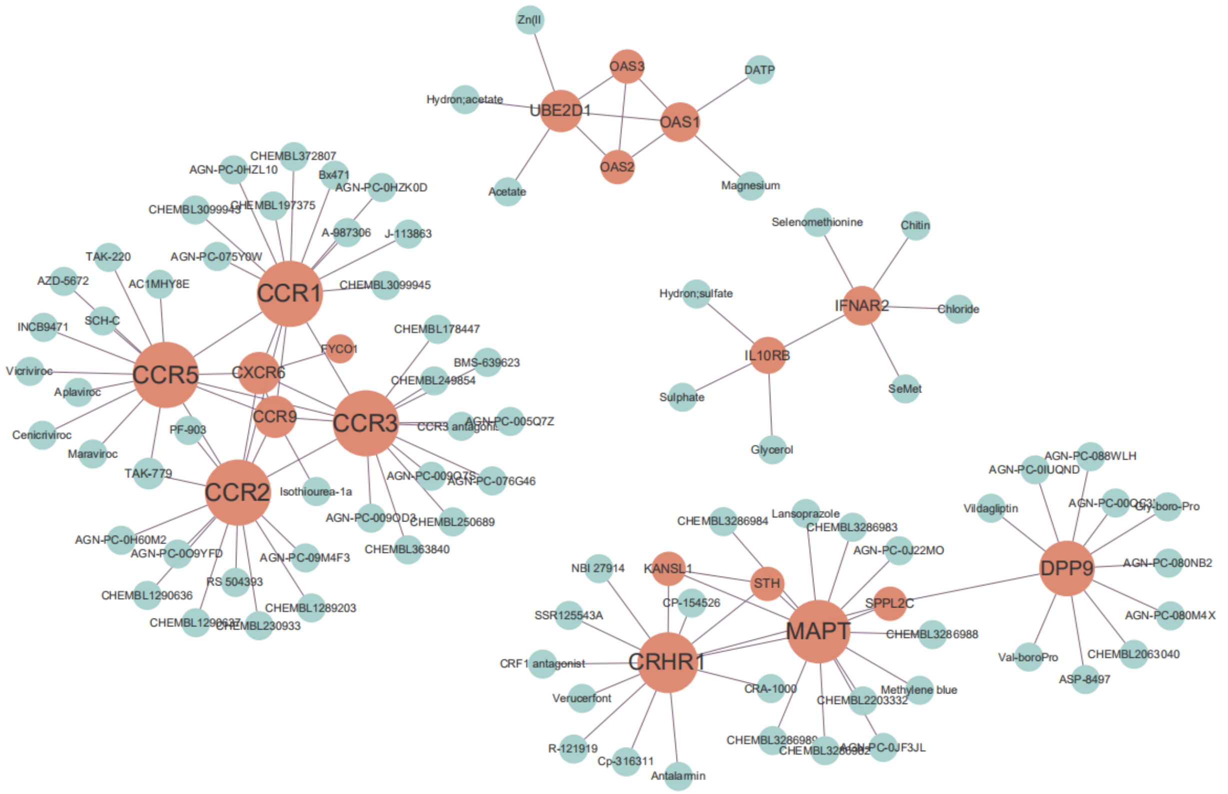


**Fig. S14. Plot of gene-drug interaction analysis for 34 risk genes.** The gene-drug information was downloaded from the STITCH database ([http://stitch.embl.de/](http://stitch.embl.de/)). The orange node represents the risk gene, and the light green color stands for the targeted drugs. For more detail information, see the method for gene-drug interaction analysis.

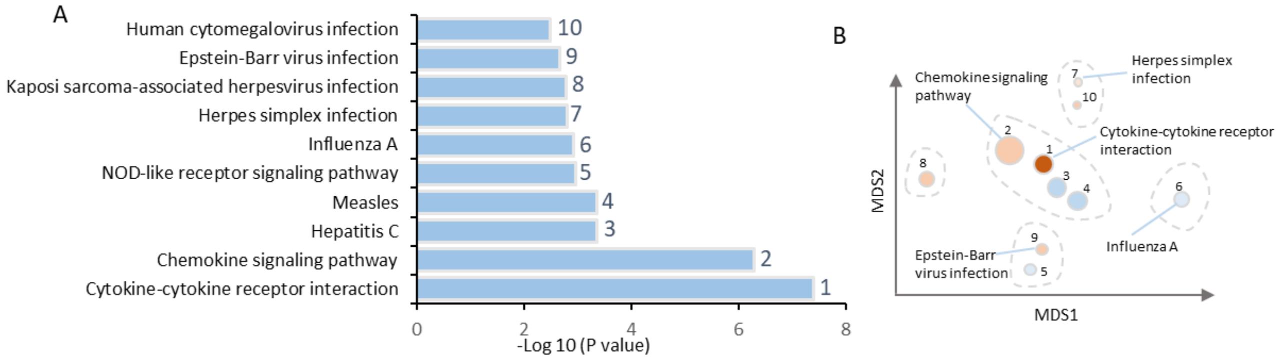


**Fig. S15. The 10 biological pathways significantly enriched by 34 risk genes based on the KEGG database.** A) Barplot showing these 10 biological pathways associated with severe COVID-19. The Arabic numeral in each bar represents the ID of each pathway ordered by the significant level (Supplemental Table S10). B) Multidimensional scaling plot for clustering these 10 pathways based on the Jaccard distance (see Methods). Color represents the significance of each pathway (red color marks the significant pathways with lowest P values).

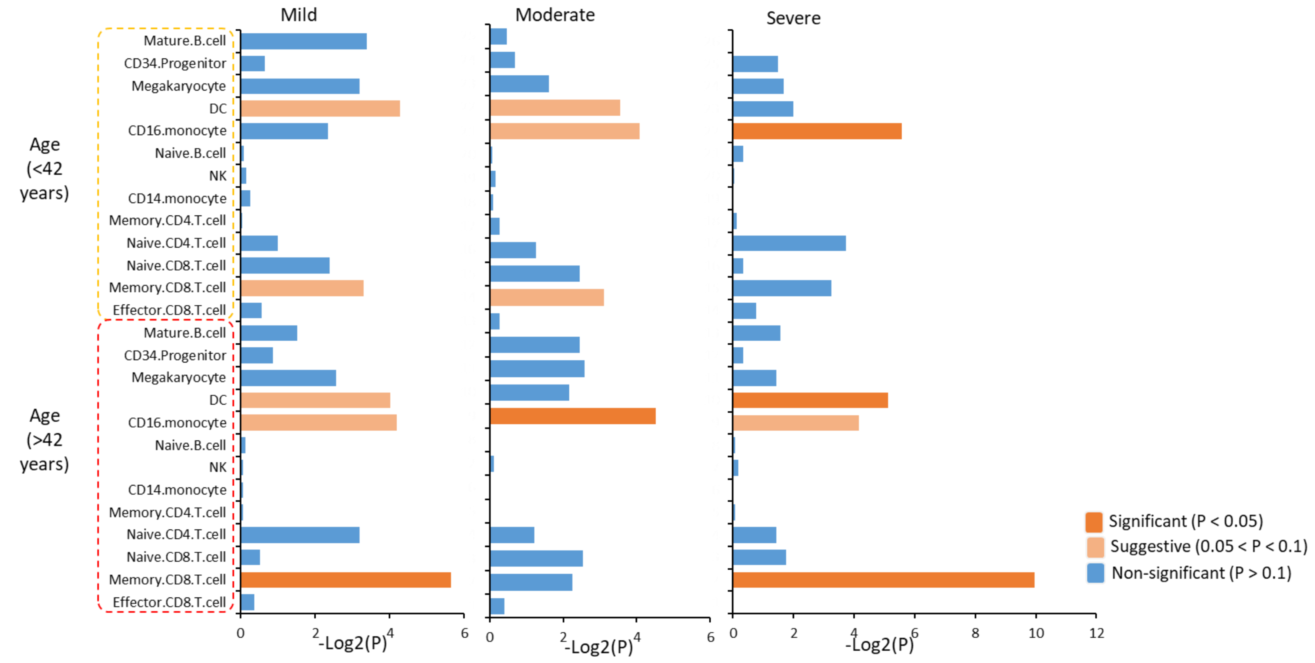


**Fig. S16. Barplots showing the results of the combination of scRNA-seq data and GWAS summary statistics based on RolyPoly among mild, moderate and severe COVID-19 patients stratified by patient's age (> median 42 years, < median 42 years).** Note: in light of there was no clinical information documented for normal controls (labeled as "NA"), thus we did not conduct the stratified integration analysis of GWAS summary data with scRNA-seq data among normal group.

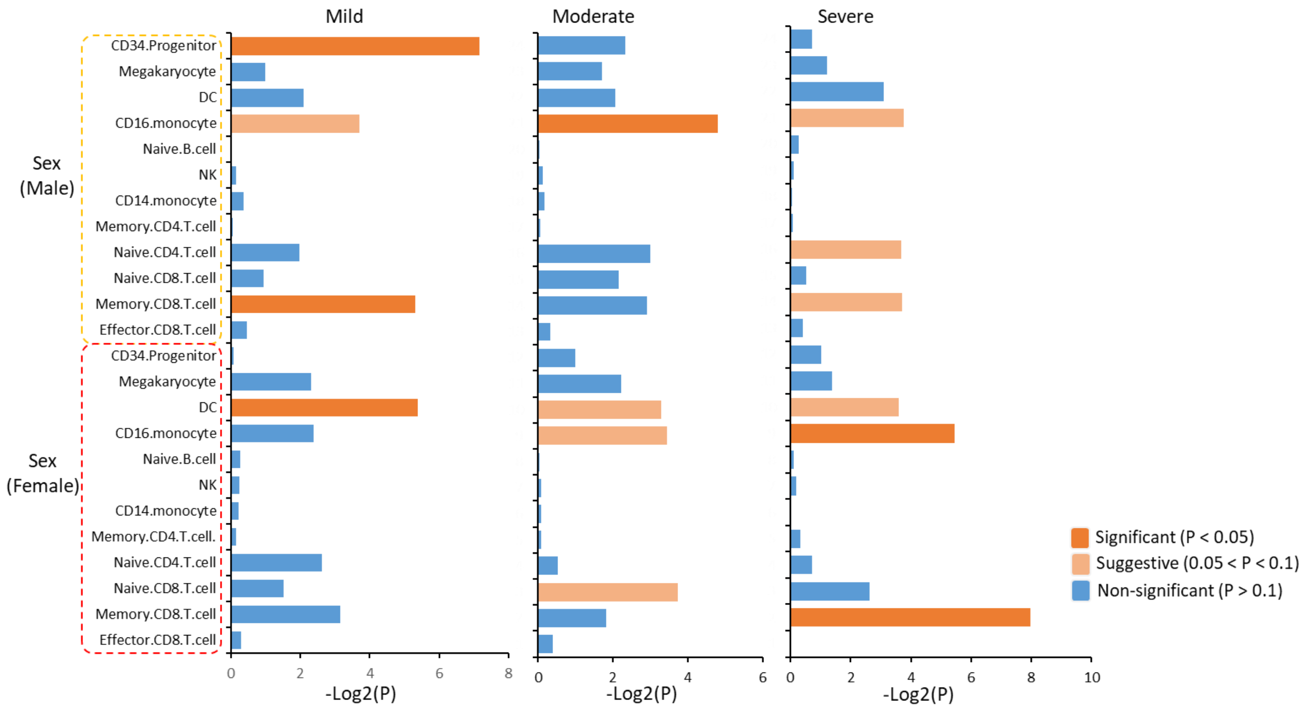


**Fig. S17. Barplots showing the results of the combination of scRNA-seq data and GWAS summary statistics based on RolyPoly among mild, moderate and severe COVID-19 patients stratified by patient's sex (Male, Female).** Note: in light of there was no clinical information documented for normal controls (labeled as "NA"), thus we did not conduct the stratified integration analysis of GWAS summary data with scRNA-seq data among normal group.

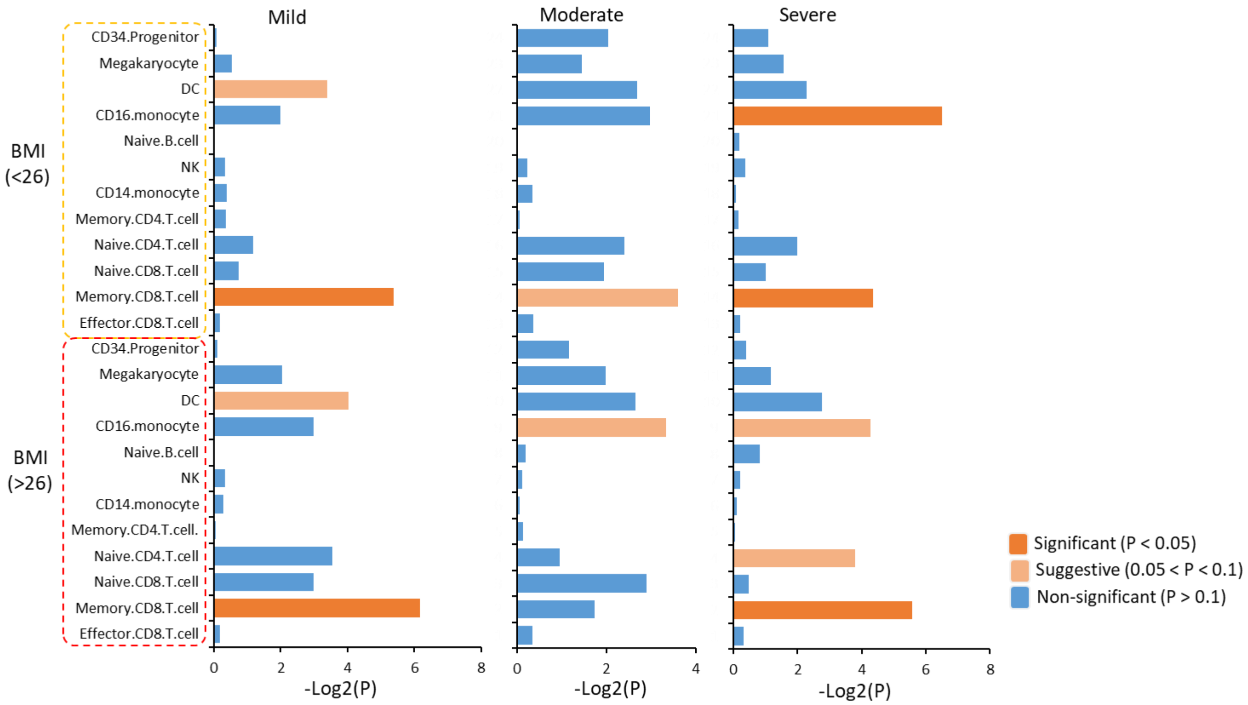


**Fig. S18. Barplots showing the results of the combination of scRNA-seq data and GWAS summary statistics based on RolyPoly among mild, moderate and severe COVID-19 patients stratified by patient's BMI (low BMI < 26, high BMI > 26).** Note: in light of there was no clinical information documented for normal controls (labeled as "NA"), thus we did not conduct the stratified integration analysis of GWAS summary data with scRNA-seq data among normal group.

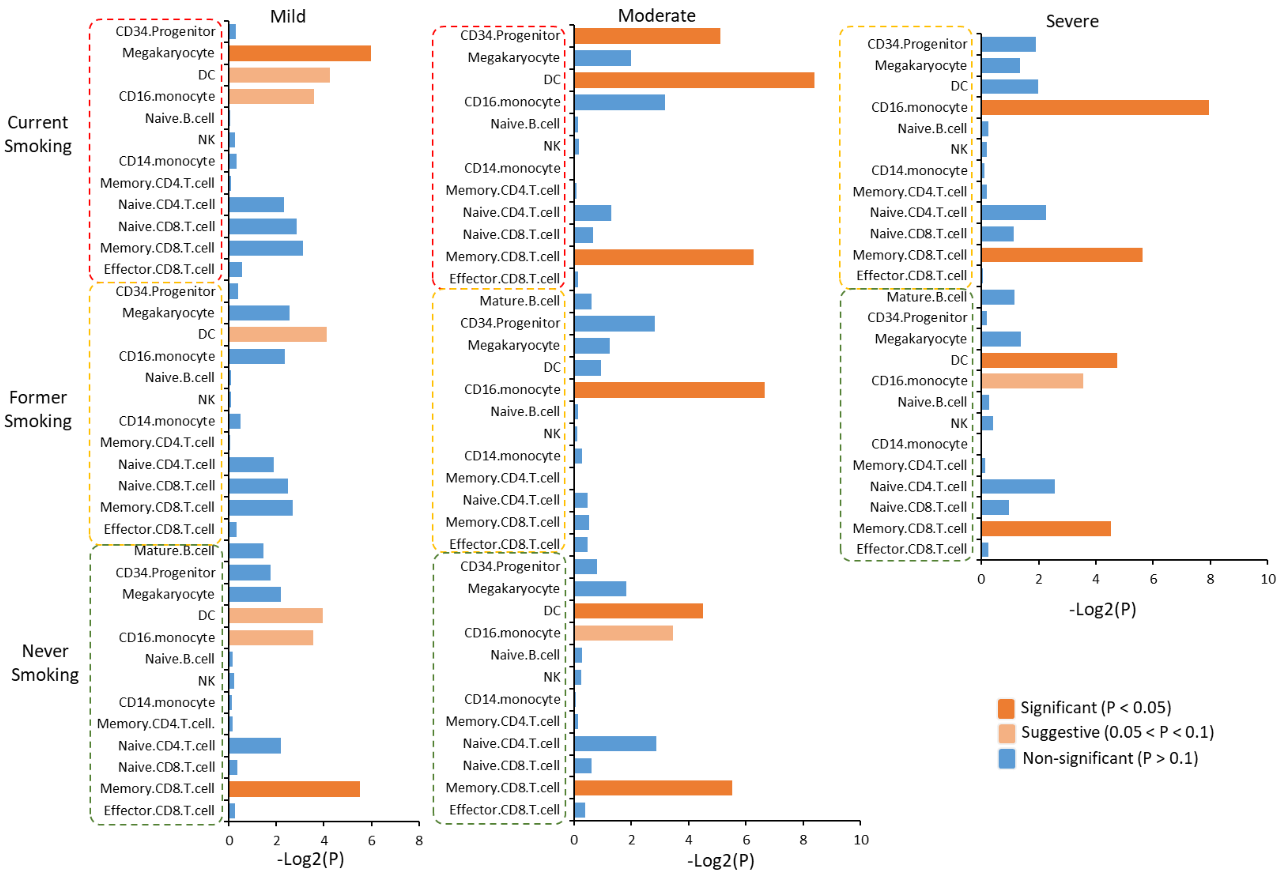


**Fig. S19. Barplots showing the results of the combination of scRNA-seq data and GWAS summary statistics based on RolyPoly among mild, moderate and severe COVID-19 patients stratified by patient's smoking status (Current smoking, former smoking, and never smoking).** Note: in light of there was no clinical information documented for normal controls (labeled as "NA"), thus we did not conduct the stratified integration analysis of GWAS summary data with scRNA-seq data among normal group.

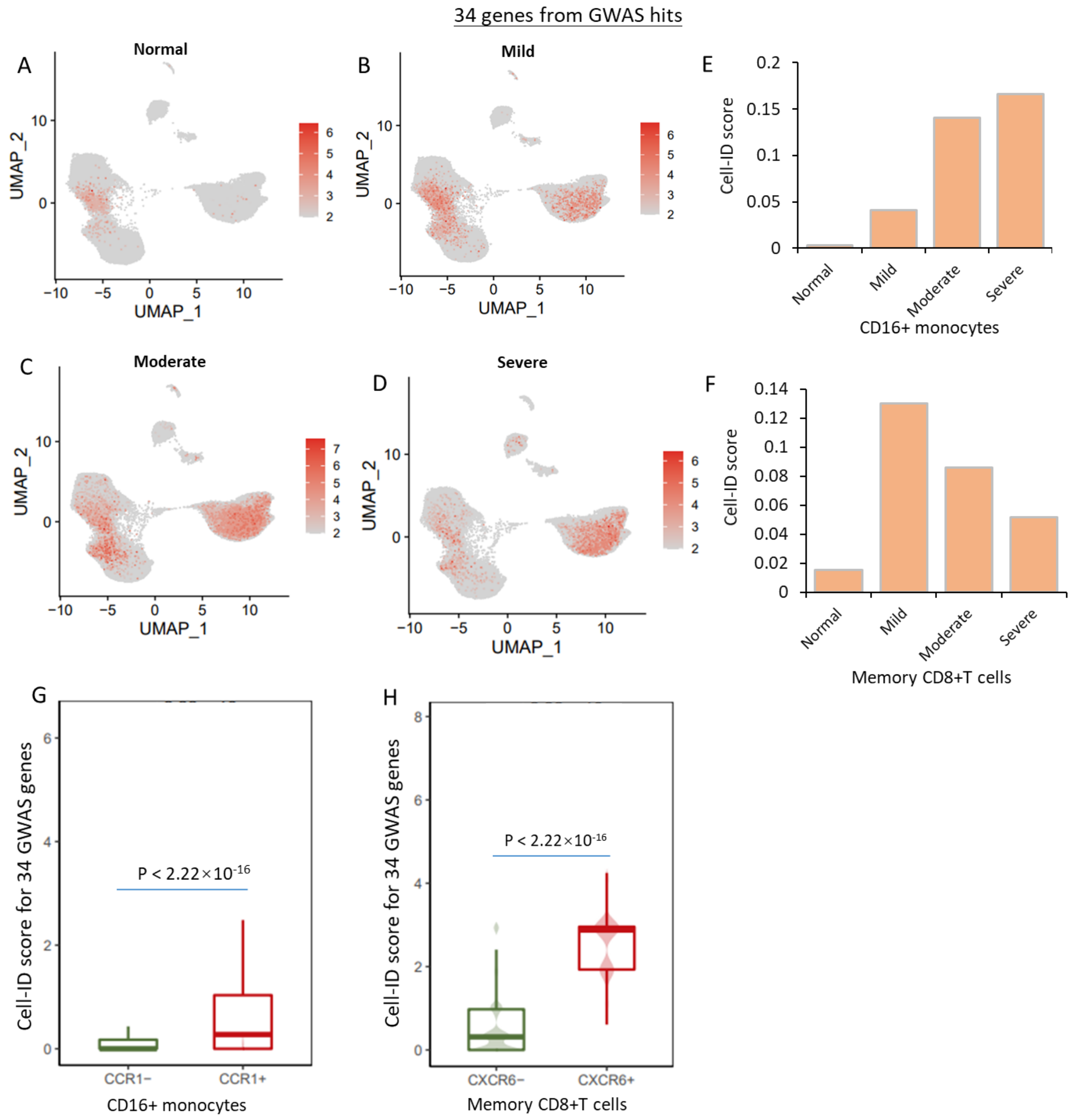


**Fig. S20. Cell-ID-based enrichment in GWAS-identified gene signatures (34 genes) of CD16+monocytes and memory CD8+ T cells using scRNA-seq dataset.** A)-D) UMAP showing the Cell-ID score of 34 GWAS hits in each individual cell among normal controls (A), mild COVID-19 patients (B), moderate COVID-19 patients (C), and severe COVID-19 patients (D). E) Barplot showing the Cell-ID score of 34 GWAS hits of CD16+ monocytes among normal controls, mild, moderate, and severe COVID-19 patients. F) Barplot showing the Cell-ID score of 34 GWAS hits of memory CD8+T cells among normal controls, mild, moderate, and severe COVID-19 patients. G) Boxplot showing the difference of Cell-ID scores of 34 GWAS hits between CCR1- and CCR1+ CD16+ monocytes. H) Boxplot showing the difference of Cell-ID scores of 34 GWAS hits between CXCR6- and CXCR6+ memory CD8+T cells.

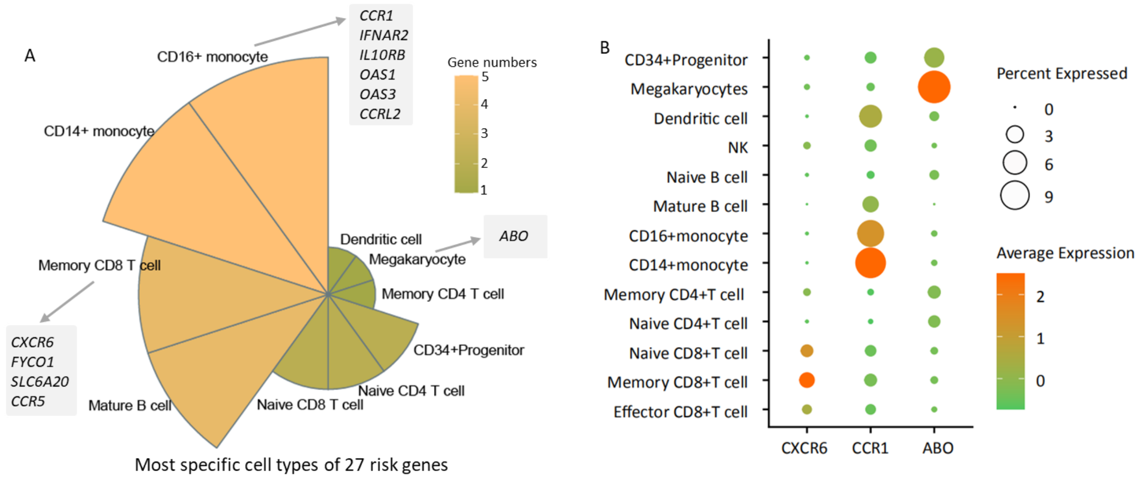


**Fig. S21. Genetics-risk genes influenced three immune cell subsets for severe COVID-19.** A) Plot showing the specific genes expressed in three identified immune cell subsets for severe COVID-19. The most specificity gene for each cell type is *CCR1* for CD16+monocytes, *CXCR6* for memory CD8+T cells, and *ABO* for megakaryocytes. B) Dot plot showing the expressed percent of three risk genes of *CXCR6*, *CCR1*, and *ABO* in each peripheral cell type in PBMCs among severe patients based on the scRNA-seq dataset #1 (E-MTAB-9357). Dot size represents fraction of cells within cell type expressing a given gene, and color intensity represents binned count-based expression amounts (log(scaled UMI +1)) among expressing cells.

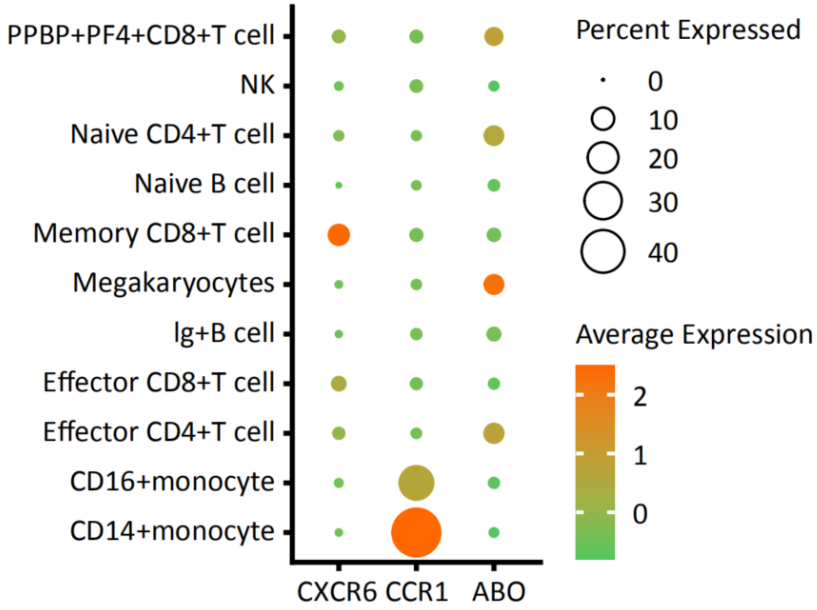


**Fig. S22. Dot plot showing the expressed percent of three risk genes of *CXCR6*, *CCR1*, and *ABO* in each peripheral cell type in PBMCs among severe patients based on two scRNA-seq dataset of #2 (GSE149689) and #3 (GSE150861)**. Dot size represents fraction of cells within cell type expressing a given gene, and color intensity represents binned count-based expression amounts (log(scaled UMI +1)) among expressing cells.

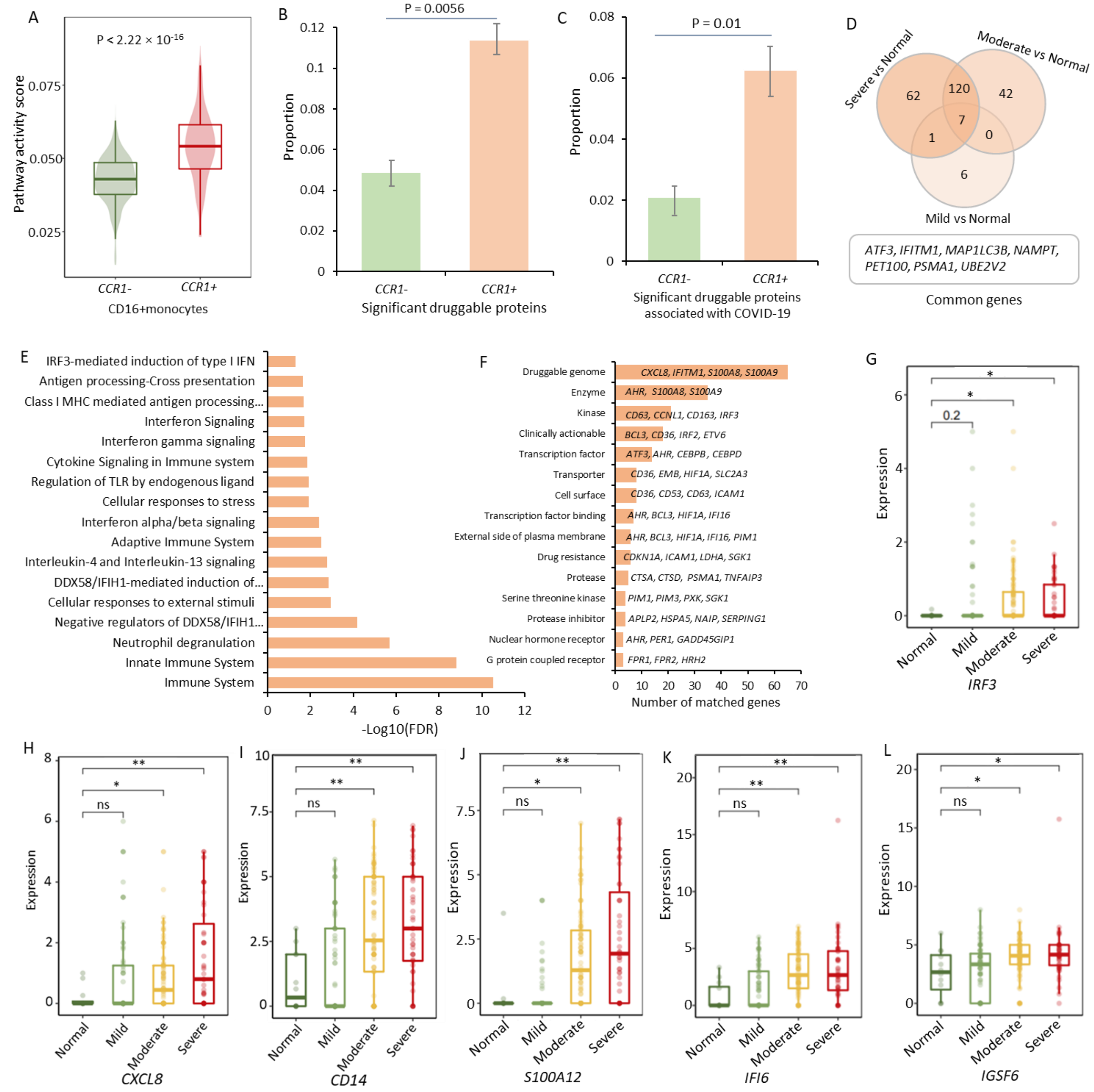


**Fig. S23. *CCR1*⁺ CD16+monocytes showing higher risk to cytokine storms among COVID-19 patients.** A) Boxplot showing the difference of pathway activity score of both cytokine-cytokine receptor interaction and chemokine signaling pathway between *CCR1*$^{+}$ and *CCR1*$^{-}$ CD16+monocytes. Two-side Wilcoxon sum-rank test was used to calculate the significance. B)-C) Barplot showing the proportion of significant druggable proteins (B) and significant druggable proteins associated with COVID-19 (C) between *CCR1*$^{+}$ and *CCR1*$^{-}$ CD16+monocytes. The hypergeometric test was applied to calculate the significance. D) Venn plot exhibiting the overlapped up-DEGs between pairwise comparisons of mild vs. normal, moderate vs. normal, and severe vs. normal. E) Functional enrichment analysis based on GO biological process for 190 up-DEGs. F) Gene-drug interaction analysis for 190 up-DEGs. G)-L) Representative up-DEGs among *CCR1*$^{+}$ CD16+monocytes showing significantly elevated expressions with increased COVID-19 severities. G) *IRF3*, H) *CXCL8*, I) *CD14*, J) *S100A12*, K) *IFI6*, and L) *IGSF6*. The cell state scores were calculated using the build-in *AddModuleScore* in Seurat.

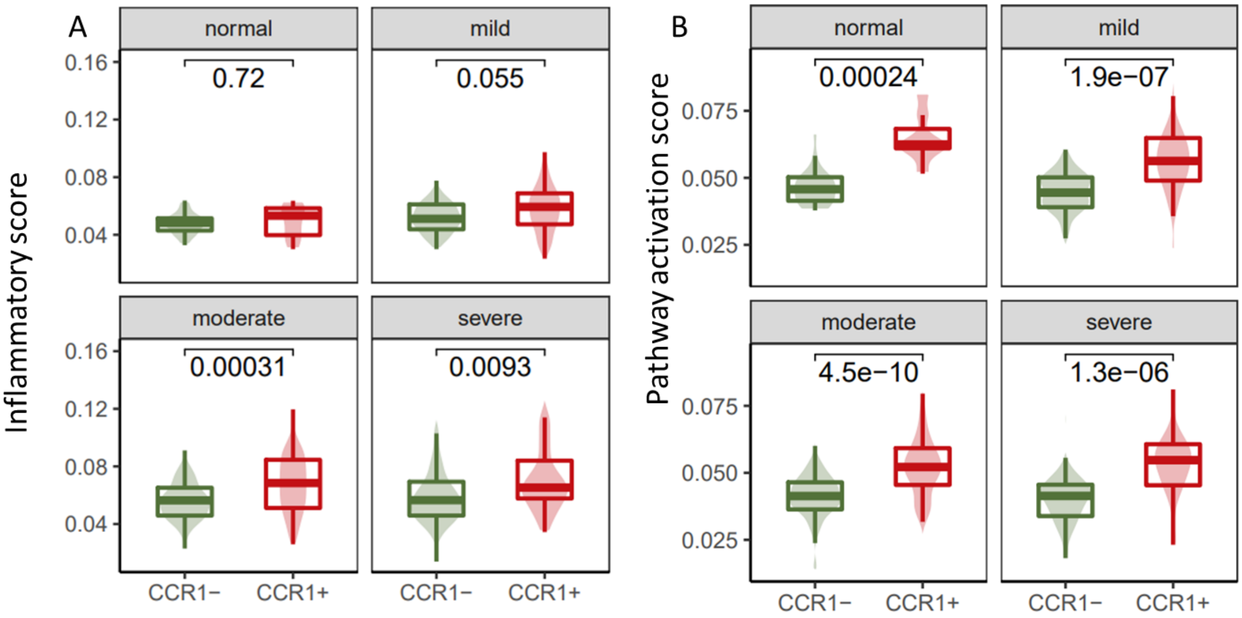


**Fig. S24. Boxplots showing the difference of inflammatory cytokine score and pathway activation score of CD16+ monocytes among normal controls, mild, moderate, and severe COVID-19 groups.** (A-B) The difference between *CCR1*$^{+}$ and *CCR1*$^{-}$ CD16+ monocytes. There were two pathways of cytokine-cytokine receptor interaction and chemokine signaling pathway used. Two-side Wilcoxon test was applied. The cell state scores were calculated using the build-in *AddModuleScore* in Seurat.

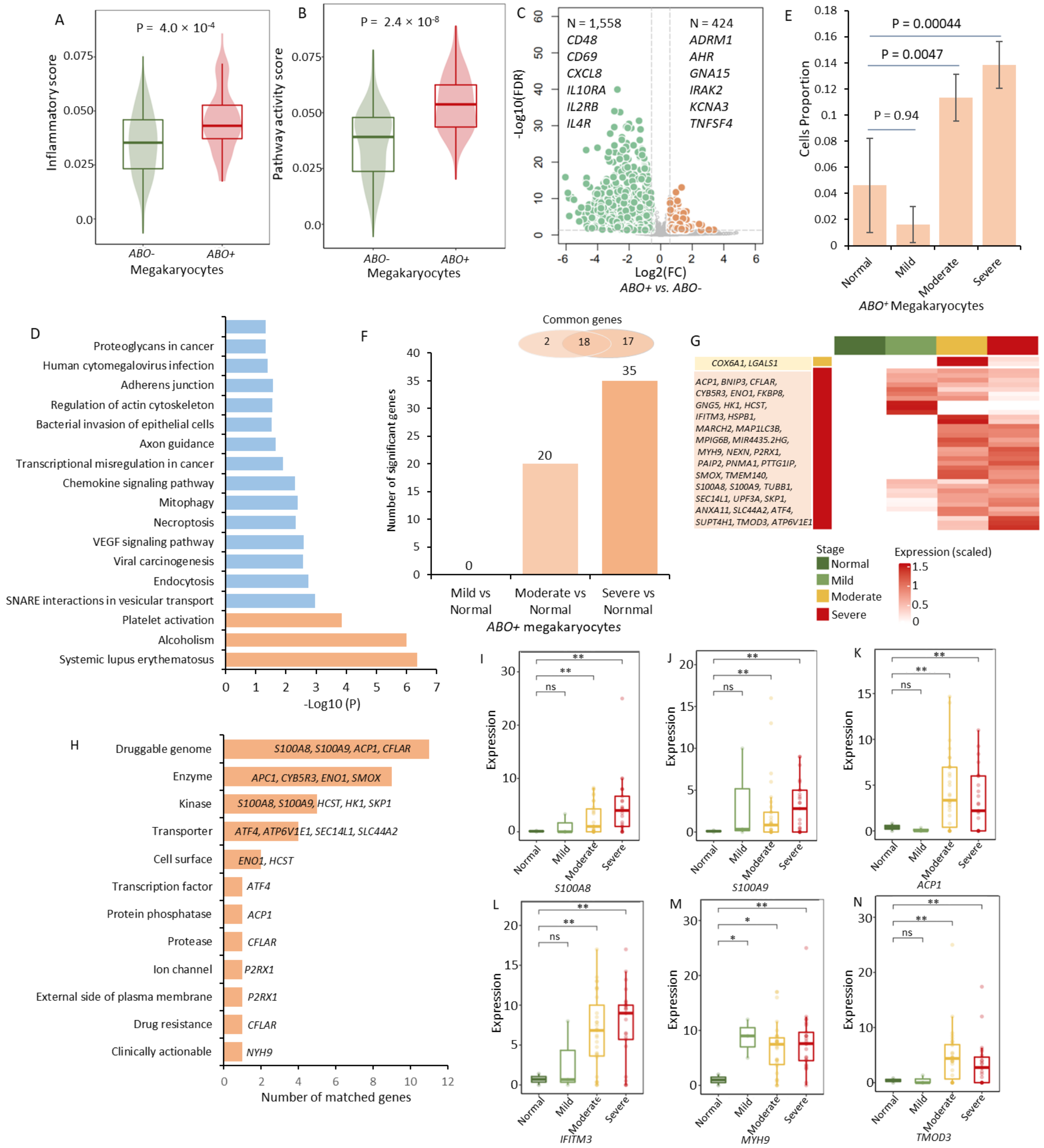


**Fig. S25.** ***ABO*$^+$ megakaryocytes contribute higher risk to cytokine storms among severe COVID-19 patients.** A)-B) Boxplot showing the difference of inflammatory cytokine score (A) and pathway active score (B) between *ABO*$^+$ and *ABO*$^-$ megakaryocytes. There were two pathways of cytokine-cytokine receptor interaction and chemokine signaling pathway used. Two-side Wilcoxon test was applied. C) Volcano plot showing differentially expressed genes between *ABO*$^+$ and *ABO*$^-$ megakaryocytes. There were 424 highly-expressed genes among *ABO*$^+$ megakaryocytes compared with *ABO*$^-$ cells. D) Pathway enrichment analysis of 424 highly-expressed genes based on the KEGG resource. E) Barplot exhibiting the proportion of *ABO*$^+$ megakaryocytes among normal controls, mild, moderate, and severe COVID-19 patients. F) Barplot showing the differentially up-DEGs from the pairwise comparisons of normal controls with different COVID-19 severities. Venn plot on the top of bar showing the overlapped up-DEGs between moderate and severe patients. G) Heatmap showing the up-DEGs from the pairwise comparisons. F) Gene-drug interaction analysis for 35 up-DEGs. I)-N) Representative up-DEGs among *ABO*$^+$ megakaryocytes showing significantly elevated expressions with increased COVID-19 severities. The cell state scores were calculated using the build-in *AddModuleScore* in Seurat.

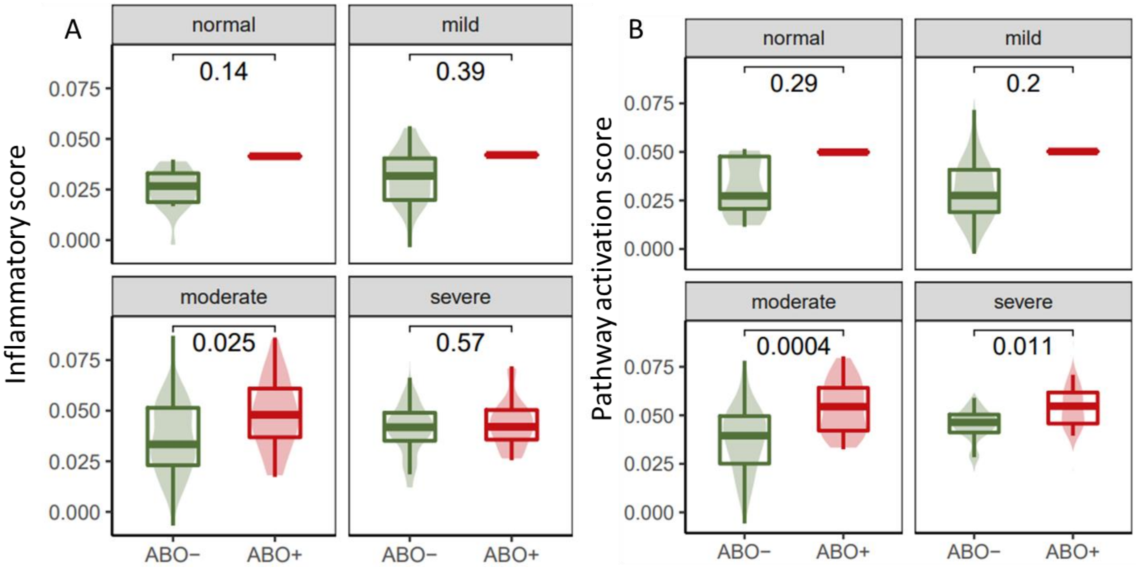


**Fig. S26. Boxplots showing the difference of inflammatory cytokine score and pathway activation score of and megakaryocytes among normal controls, mild, moderate, and severe COVID-19 groups.** (A-B) The difference between *ABO*$^{+}$ and *ABO*$^{-}$ megakaryocytes. There were two pathways of cytokine-cytokine receptor interaction and chemokine signaling pathway used. Two-side Wilcoxon test was applied. The cell state scores were calculated using the build-in *AddModuleScore* in Seurat.

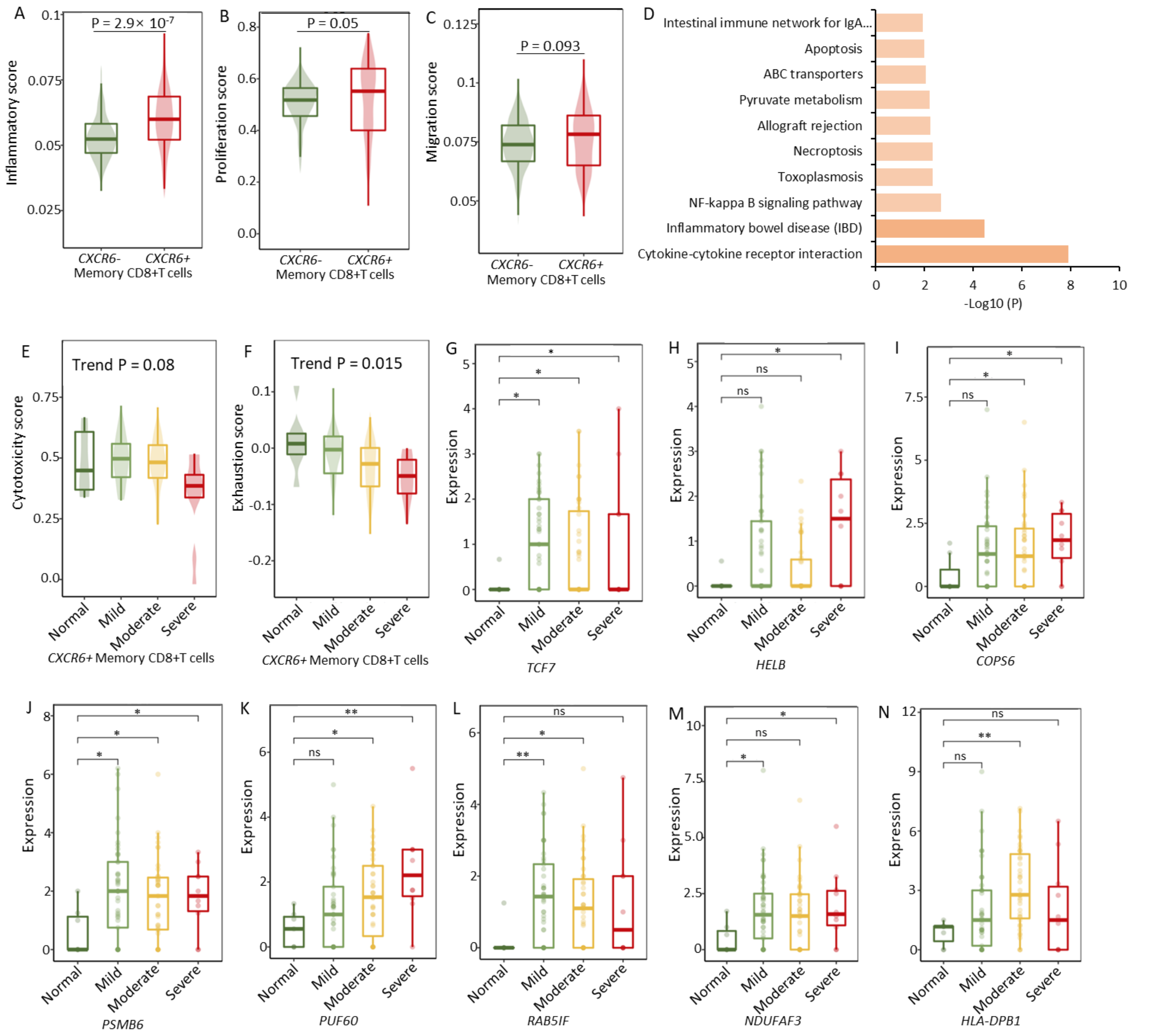


**Fig. S27. Evidence showing the multi-functionality of *CXCR6*$^{+}$ memory CD8+T cells for severe COVID-19.** A)-C) Boxplots showing the difference of inflammatory score (A), proliferation score (B), and migration score (C) between *CXCR6*$^{+}$ and *CXCR6*$^{-}$ memory CD8+T cells. Two-side Wilcoxon sum-rank test was used. D) Pathway enrichment analysis of 158 highly-expressed genes based on the KEGG resource. E)-F) Boxplots showing the cytotoxicity score (E) and exhaustion score (F) of among normal, mild, moderate, and severe groups. G)-N) Representative up-DEGs among *CXCR6*$^{+}$ memory CD8+T cells showing significantly elevated expressions with increased COVID-19 severities. G) *TCF7*, H) *HELB*, I) *COPS6*, J) *PSMB6*, K) *PUF60*, L) *RAB5IF*, M) *NDUFAF3*, and N) *HLA-DPB1*. The cell state scores were calculated using the build-in *AddModuleScore* in Seurat.

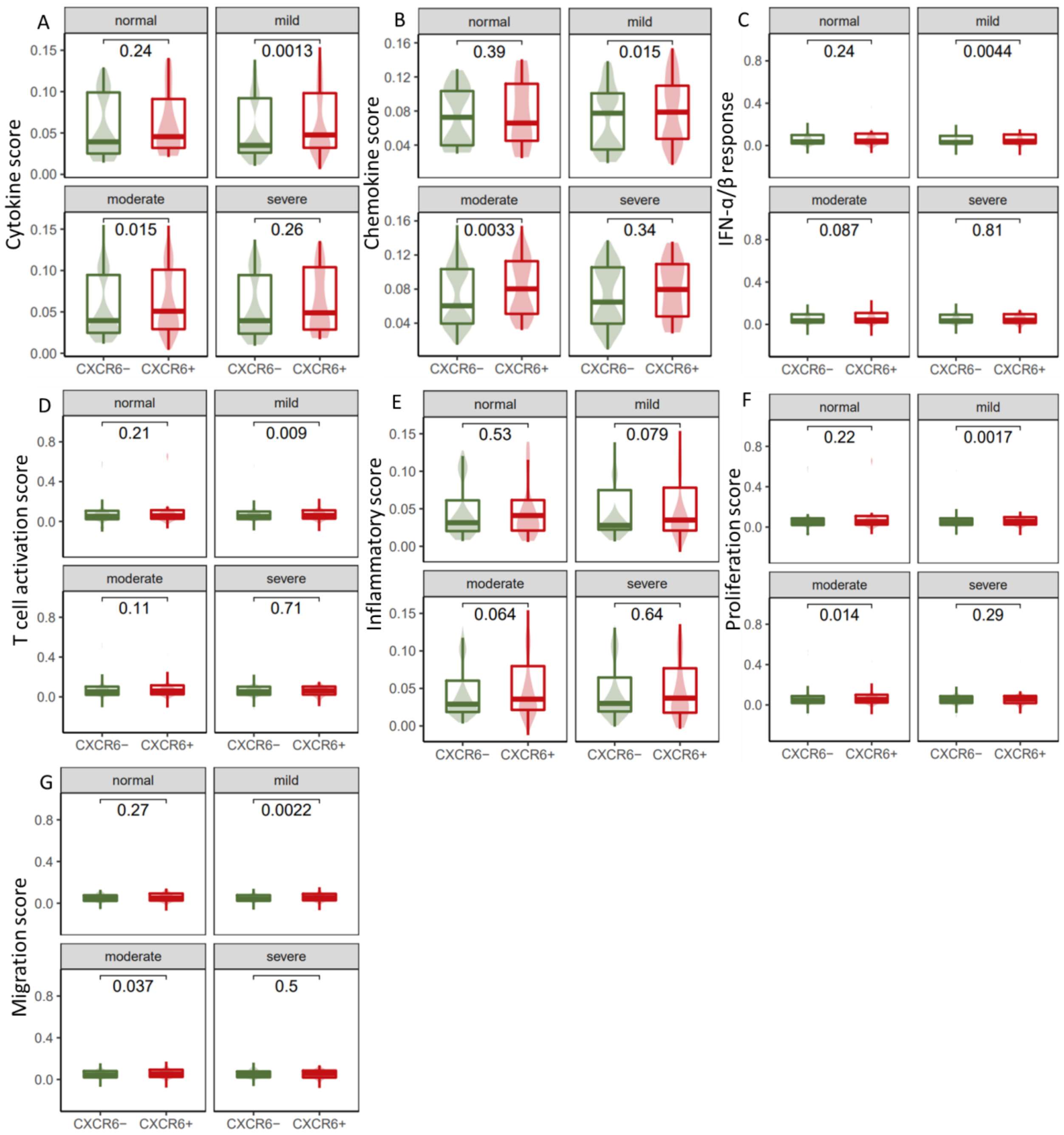


**Fig. S28. Boxplots showing the difference of cytokine score (A), chemokine score (B), IFN-α/β response (C), T cell activation score (D), Inflammatory score (E), proliferation score (F), and migration score (G) between *CXCR6*$^{+}$ and *CXCR6*$^{-}$ memory CD8+T cells among normal controls, mild, moderate, and severe COVID-19 patients**. Two-side Wilcoxon sum-rank test was used. The cell state scores were calculated using the build-in *AddModuleScore* in Seurat.

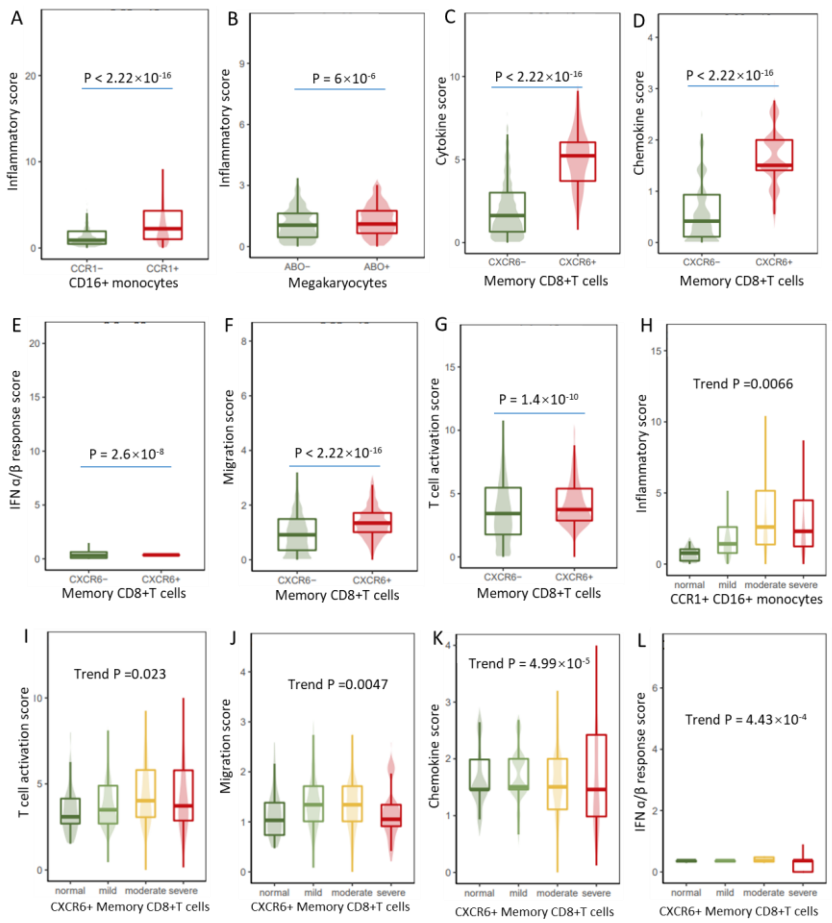


**Fig. S29. Cell-ID-based enrichment in functional terms of CD16+monocytes, megakaryocyte, and memory CD8+ T cells using scRNA-seq dataset.** A) Boxplot showing the difference of Cell-ID scores of inflammatory cytokine score between *CCR1*$^{-}$ and *CCR1*$^{+}$ CD16+ monocytes. B) Boxplot showing the difference of Cell-ID scores of inflammatory cytokine score between *ABO*$^{-}$ and *ABO*$^{+}$ megakaryocytes. C)-G) Boxplot showing the difference of Cell-ID scores of cytokine score (C), chemokine score (D), IFN-α/β response score (E), migration score (F), and T cell activation score (G) between *CXCR6*$^{-}$ and *CXCR6*$^{+}$ memory CD8+T cells. H) Boxplots showing the inflammatory cytokine score of *CCR1*$^{+}$ CD16+ monocytes among normal, mild, moderate, and severe groups. I)-L) Boxplots showing the T cell activation score (I), migration score (J), chemokine score (K), and IFN-α/β response score (L) of *CXCR6*$^{+}$ memory CD8+T cells among normal, mild, moderate, and severe groups.

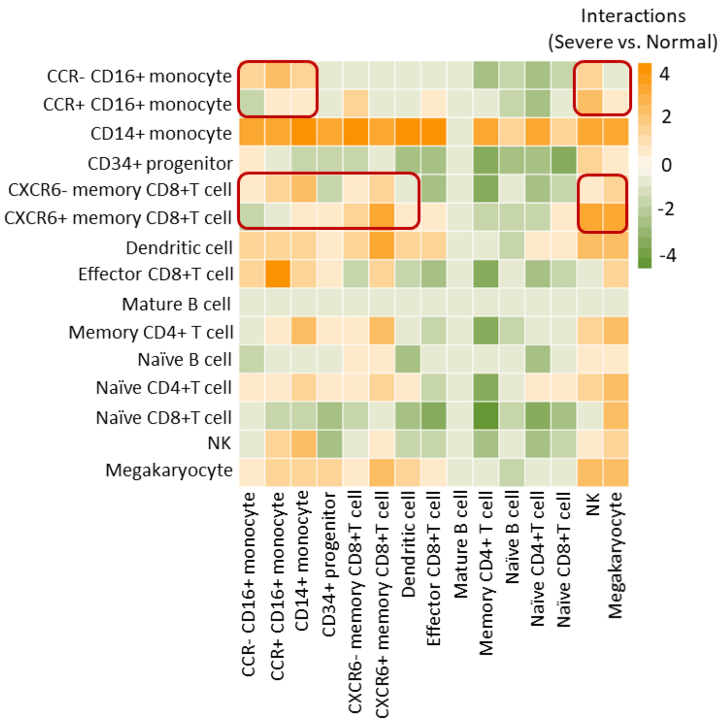


**Fig. S30. Differences in the number of predicated cell-to-cell interactions in PBMCs, comparing severe COVID-19 patients with normal controls.** Color legend represents the differential number of cellular interactions. Red rectangle marks the elevated interactions of both *CCR1*$^{+}$ CD16+monocytes and *CXCR6*$^{+}$ memory CD8+T cells with other immune cells in PBMCs.

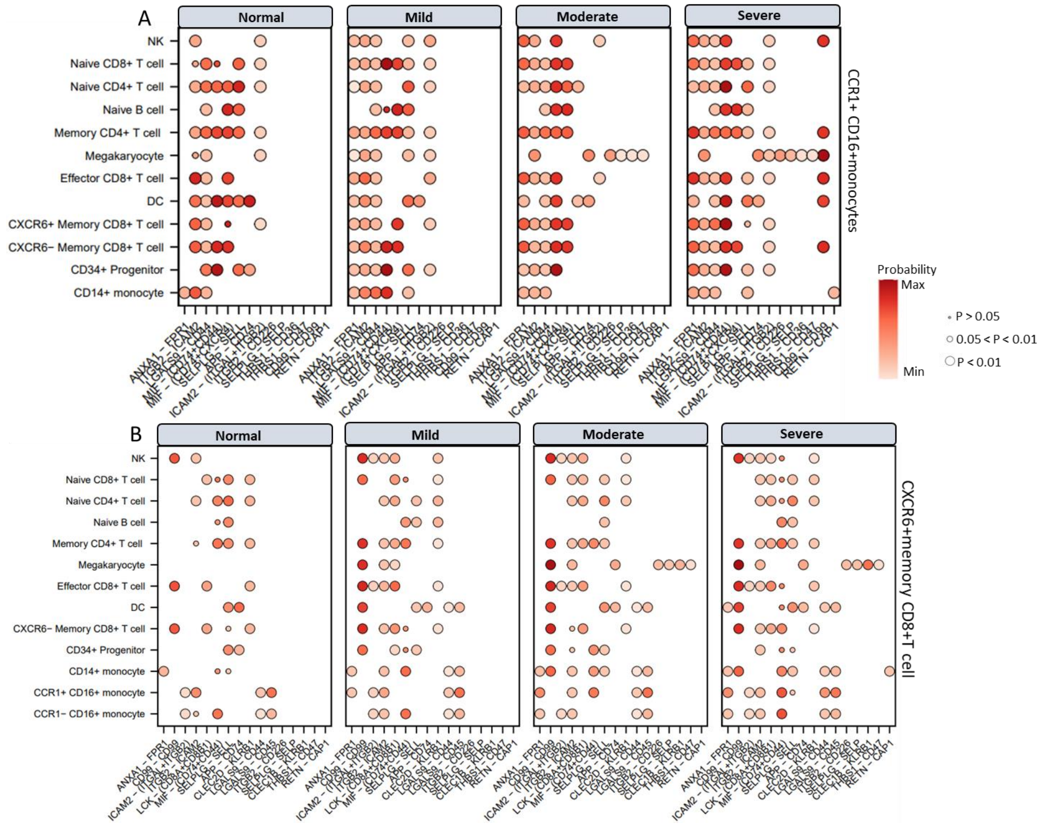


**Fig. S31. Predicted cellular interaction of both *CCR1*$^{+}$ CD16+ monocytes and *CXCR6*$^{+}$ memory CD8+T cells with other immune cells in PBMCs.** (A) Predicted cellular interactions of *CCR1*$^{+}$ CD16+ monocytes with other immune cells in PBMCs, comparing severe COVID-19 with mild, moderate COVID-19, and normal controls. (B) Predicted cellular interactions of *CXCR6*$^{+}$ memory CD8+T cells with other immune cells in PBMCs, comparing severe COVID-19 with mild, moderate COVID-19, and normal controls.

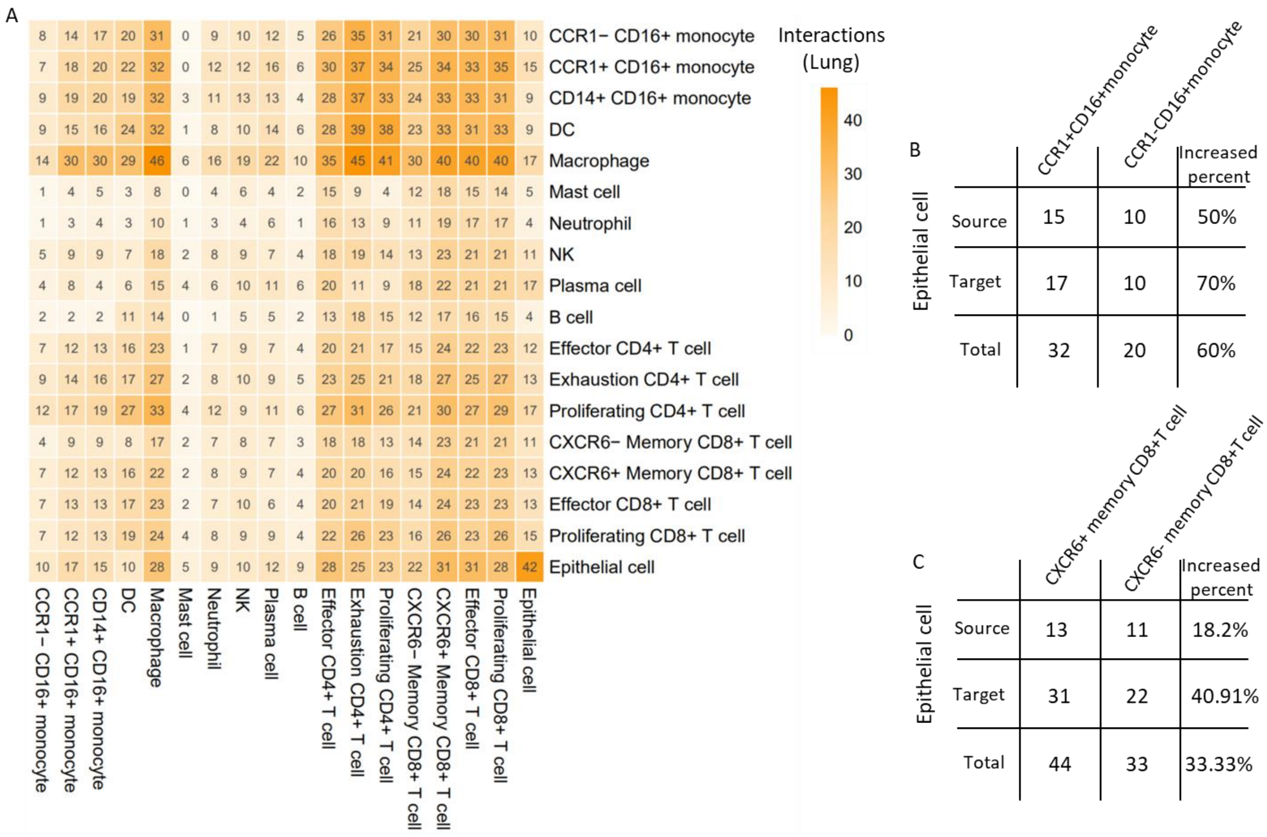


**Fig. S32. Prediction of cell-to-cell interactions of cells in BALFs.** A) The number of predicated cellular interactions of cells in BALFs. Color legend represents the number of predicted cellular interactions. The Arabic numerals in the heatmap represent the specific number of cellular interactions between pairwise cell types in BALFs. B) The increased percent of cellular interactions of *CCR1*$^{+}$ CD16+monocytes with epithelial cells compared with *CCR1*$^{-}$ CD16+monocytes. C) The increased percent of cellular interactions of *CXCR6*$^{+}$ memory CD8+T cells with epithelial cells compared with *CXCR6*$^{-}$ memory CD8+T cells.
